# Removal of unwanted variation in pseudobulk analysis of single-cell RNA sequencing data and the leveraging of pseudoreplicates

**DOI:** 10.1093/nargab/lqaf179

**Published:** 2025-12-08

**Authors:** Sofía Prieto León, Ewoud De Troyer, Helena Geys, Koen Van den Berge, Olivier Thas

**Affiliations:** Data Science Institute and I-BioStat, Hasselt University, 3500 Hasselt, Belgium; Statistics and Decision Sciences, Johnson & Johnson Innovative Medicine, 2340 Beerse, Belgium; Data Science Institute and I-BioStat, Hasselt University, 3500 Hasselt, Belgium; Statistics and Decision Sciences, Johnson & Johnson Innovative Medicine, 2340 Beerse, Belgium; Statistics and Decision Sciences, Johnson & Johnson Innovative Medicine, 2340 Beerse, Belgium; Data Science Institute and I-BioStat, Hasselt University, 3500 Hasselt, Belgium; Department of Mathematics, Computer Science and Statistics, Ghent University, 9000 Ghent, Belgium; National Institute for Applied Statistics Research Australia (NIASRA), University of Wollongong, 2522 Wollongong, New South Wales, Australia

## Abstract

Removing unwanted variation (RUV) is key for accurate biological interpretation in high-throughput sequencing studies. However, no standardized approach exists for pseudobulked single-cell RNA-sequencing (scRNA-seq) data. Improper implementation of RUV methods may remove biological information, jeopardizing power and false positive control in differential expression analysis. We evaluate the impact of three implementation strategies (‘trails’) in three RUV methods (RUV2, RUVIII, RUV4) using simulated and real biological signals in pseudobulked scRNA-seq data. Effects of technical noise under confounding and model misspecification conditions are also considered. Additionally, we introduce a novel strategy, RUVIII PBPS, to remove unwanted variation in pseudobulk differential expression analyses with insufficient technical replicates or negative control genes. Our analysis demonstrates that removing unwanted variation per cell type with RUV2 or RUVIII extracts factors associated with technical noise and controls the false discovery rate (FDR), even in the presence of confounding. RUVIII PBPS successfully controls the FDR when other standard RUV methods cannot be used due to missing technical replicates, dependence between the factor of interest and the sources of unwanted variation, and lack of plausible negative control genes.

## Introduction

External factors, for instance reagent batches, timing, variation across sequencing plates, and variation across experiment locations, often introduce technical noise in high-dimensional biological data. This technical noise is often referred to as batch effects, and can lead to erroneous biological conclusions [1] and loss of power for differential expression detection [2]. Nevertheless, information about the batch effects is not always available. In that context, RUV (remove unwanted variation) methods were introduced to estimate and correct for technical noise and other variation sources not wanted in the data; they are particularly used in the context of gene expression analysis [3]. RUV methods estimate a set of latent variables that are associated with the unwanted variation. These latent variables are also called factors of unwanted variation or unwanted factors.

The first RUV methods were developed for microarray and bulk RNA-seq experiments and used a linear regression model like the one presented in Equation (1) for describing the $N \times G$ log_2_-transformed gene expression matrix $\log _2(\boldsymbol{\mathbf { Y}})$, with $N$ the number of samples (rows) and $G$ the number of genes (columns). This matrix is modelled as a linear combination of the $N\times Q$ design matrix $\boldsymbol{\mathbf { X}}$ and the $N \times K$ unwanted factor matrix $\boldsymbol{\mathbf { W}}$, with $Q$ the number of factors of interest and $K$ the number of unknown unwanted factors. In particular,


(1) \begin{eqnarray*}
\log _2(\boldsymbol{\mathbf { Y}}) = \boldsymbol{\mathbf { W}} \boldsymbol{\mathbf { \alpha }} + \boldsymbol{\mathbf { X}} \boldsymbol{\mathbf { \beta }} + \epsilon ,
\end{eqnarray*}

where the parameters $\boldsymbol{\mathbf { \beta }}$ of dimension $Q\times G$ and $\boldsymbol{\mathbf { \alpha }}$ of dimension $K\times G$ denote the effect parameter of the factors of interest and unwanted factors on the response, respectively, and $\epsilon$ is the error term. The $N$ samples and $G$ genes are assumed to be independent.

**Figure 1.**
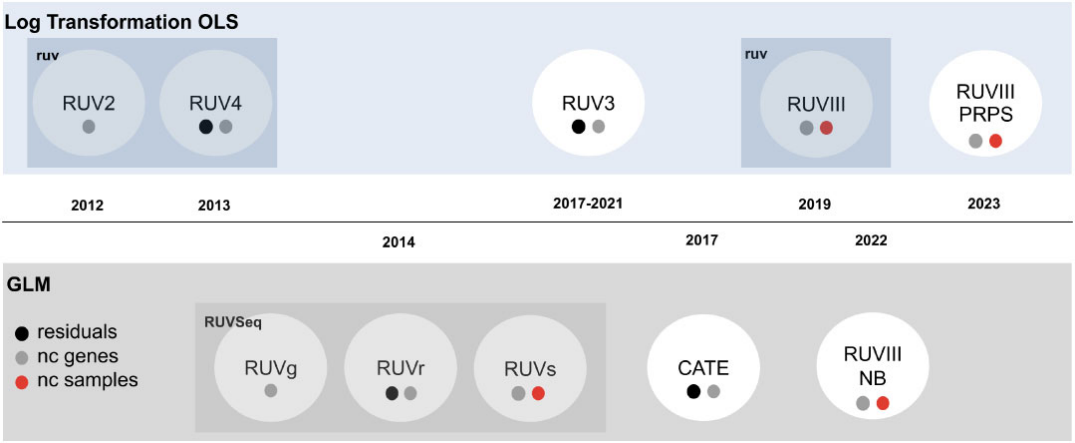
RUV methods, with the filled circles below each method denoting their classification by the approach used to estimate the unwanted hidden factors: residuals, negative control genes (nc genes), and negative control samples (nc samples). The top row lists methods that rely on ordinary least squares (OLS) applied to the log_2_-transformed data, while the bottom row are methods using a generalized linear model (GLM) applied to counts (as in RNA sequencing). The methods included in the two R packages *ruv* and *RUVSeq* are highlighted using shaded boxes.

In general, RUV methods estimate $\boldsymbol{\mathbf { W}}$ using exploratory factor analysis. To perform a differential expression analysis (DEA), the $\boldsymbol{\mathbf { W}}$ matrix can be added as a covariate. Additionally, it is possible to compute a normalized matrix $\log _2(\boldsymbol{\mathbf { Y}}^{*})$ by regressing out the linear effect of $\boldsymbol{\mathbf { W}}$ from the log_2_-expression matrix $\log _2(\boldsymbol{\mathbf { Y}})$. Performing the DEA with the normalized matrix is not advised because of the possible removal of biological information [4, 5]. The variants of the method differ in the way they estimate $\boldsymbol{\mathbf { W}}$, and the model applied in DEA. An overview of the main RUV methods is given in Fig. 1.

The numbers in some methods’ names represent the number of steps taken to estimate $\boldsymbol{\mathbf { W}}$ and $\boldsymbol{\mathbf { \beta }}$. This is the case for RUV2 [3], the first RUV method, which assumes the existence of a known gene set that is not influenced by the biological condition of interest, but captures some of the unwanted variation in the data, referred to as negative control genes, and represented as $\mathcal {G}$. It performs factor analysis on the $\log _2$ expression matrix from the negative control genes, $\log _2(\boldsymbol{\mathbf { Y}})_{\mathcal {G}}$. The RUV4 method [4] uses a four-step estimation procedure and assumes independence between the unwanted factors and the factor of interest; it claims to improve the quality of $\boldsymbol{\mathbf { W}}$ estimation by including both the negative control genes and the residuals of the linear model $\log _2(\boldsymbol{\mathbf { Y}})=\boldsymbol{\mathbf { X}}\boldsymbol{\mathbf { \beta }}+\epsilon$. The estimation procedures of RUV2 and RUV4 are unified in the RUV3 method [6], providing a mathematical equivalence between them. The RUVIII method [7] (different from RUV3) assumes the existence of technical replicates from the same sample that capture the unwanted variation, called negative control samples, and combines the use of negative control genes and negative control samples for estimating $\boldsymbol{\mathbf { W}}$. Finally, RUVIII-PSPR [8] proposes an alternative to RUVIII when a proper set of negative control samples is missing, by creating pseudo-samples using the average log-expression from samples with similar biology and exposed to similar sources of unwanted variation.

Other RUV methods apply similar ideas using a negative binomial model: e.g. the RUVg, RUVr, and RUVs methods [5]; the CATE method [9]; and the single-cell extension of RUVIII, RUVIIINB [10].

The RUV methods were not the first to use factor analysis for estimating systematic unwanted variation. Previously, work on surrogate variable analysis [11] had already proposed estimating additional variables that explain the unwanted variation. However, RUV methods use negative controls, as opposed to residuals. Other batch correction methods such as ComBat [12] and ComBat-seq [2] use an empirical Bayes framework to adjust for technical effects, rely on available information, and have a poor performance in unbalanced designs [13].

**Figure 2.**
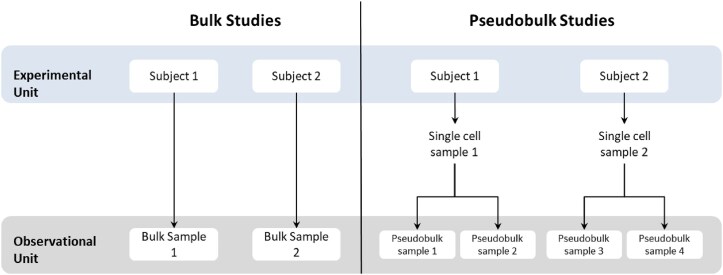
Hierarchical structure of samples from bulk and pseudobulk studies. In bulk studies with no technical replicates, each experimental unit has only one observational unit. In pseudobulk studies with, for instance, two cell types, there are two observational units (pseudobulk samples) for each experimental unit (single-cell sample); therefore, within-individual correlation is present only in pseudobulk samples.

A $C \times G$ single-cell RNA sequencing matrix $\boldsymbol{\mathbf { Y}}$ consists of gene expression measurements from $G$ genes in $C$ cells, collected from $N$ samples (also referred to in the literature as assays) that are extracted from $J$ subjects. Thus, the cells are observational units and the subjects typically are experimental units (see Fig. 2). Furthermore, if there is only one sample from each subject, then $N=J$; on the contrary, if there are technical replicates or repeated measures of the same subject, then $N>J$. Additionally, irrespective of the subject they come from, the cells are classified into $T$ different cell types.

When one is interested in assessing differential expression between conditions, for each gene in each cell type, the current practice is to ‘pseudobulk’ the gene expression matrix, i.e. aggregate the single-cell RNA sequencing gene expression matrix by summing the counts of cells from same cell type and sample into $S$ pseudobulk samples [14]. Note that if every sample from a single-cell RNA sequencing experiment (from now on, a single-cell sample) observes cells from all cell types, then $S=N\times T$. The pseudobulk $S \times G$ matrix $\boldsymbol{\mathbf { Y}}_p$ is used to perform differential expression analyses using the pseudobulk samples from a particular subpopulation (cell type) at a time [15].

Pseudobulk studies differ from bulk studies due to, among other things, the correlation between cells from the same sample and, consequently, the correlation between pseudobulk samples originating from the same single-cell sample. As illustrated in Fig. 2, in a pseudobulk study, as in single-cell studies, one experimental unit has multiple observational units, and these observational units are expected to be correlated. This correlation violates the assumption of independent samples in regression models [16, 17], complicating the adaptation of bulk RUV methods to pseudobulk studies.

Another challenge for implementing most of the RUV strategies in the pseudobulk setting is the identification of true negative control genes. While a few methods have been proposed to find stably expressed genes [18, 19], there is no consensus about a general strategy. Nevertheless, using the wrong set of negative control genes can induce low true positive rates (TPR) and inflated false discovery rates (FDR) in differential expression analysis, as displayed in Appendix F. Under uncertainty about the true negative control genes, setting all genes as negative controls in methods like RUVIII, which also use negative control samples, still yields good performance [4, 5]; however, many studies do not have the technical replicates required to use the method. The pseudoreplicates pseudosamples (PRPS) technique has been previously introduced in bulk studies to implement RUVIII [7] without technical replicates and is further discussed in Appendix B. Contrary to the estimation of the unwanted factors, the use of PRPS in pseudobulk studies does not need major considerations, but the method requires many samples.

In Section 2.1, we introduce the pseudobulk method, and in Section 2.2, we compare three strategies (trails) for using the RUV methods of the *ruv* R package [20] in the pseudobulk context. Next, in Section 2.3 we define pseudobulk pseudosamples (PBPS), an alternative to PRPS. In Section 3.1, we evaluate the performance of the RUV trails and the PBPS method using real datasets with simulated differential expression. In Section 3.2, we apply the methods in the lupus case study and evaluate the use of different sets of negative control genes. Finally, we discuss our findings in Section 4.

## Materials and methods

### The pseudobulk method

The pseudobulk methods usually sum the rows (cells) of the count matrix $\boldsymbol{\mathbf { Y}}$ by single-cell samples $n=1,..., N$ and cell types $t=1,..., T$. Each unique combination of single-cell sample and cell type constitutes a sample $s=1,..., S$ in the pseudobulk context. This aggregation yields the pseudobulk count matrix, denoted as $\boldsymbol{\mathbf { Y}}_p$, with the same $G$ columns as the original count matrix $\boldsymbol{\mathbf { Y}}$, and $S$ rows, where $S = NT$ if each cell type is present in each single-cell sample. Furthermore, $S_t$ is the number of pseudobulk samples corresponding to the cell type $t$. After pseudobulking, one typically assesses differential expression for each cell type separately and applies common differential expression methods designed for bulk RNA-Sequencing (e.g. edgeR) [21, 22]. This strategy removes within-individual correlation, i.e. the correlation between pseudobulk samples from the same single-cell sample [23].

### Pseudobulk RUV trails

RUV methods applied to microarray and bulk studies estimate the unwanted factors on the entire dataset. However, pseudobulk studies often have within-individual correlation. There are no clear guidelines that account for the sample’s correlation when applying the RUV methods on pseudobulk datasets. Therefore, we explore three approaches for implementing the RUV algorithms in pseudobulk datasets; these are referred to as ‘trails’. Note that, to avoid logarithms of zero, a pseudo-count of 1 is added to each value of $\boldsymbol{\mathbf { Y}}_p$, but for simplicity this adjustment is omitted from all equations.

#### Trail 1: all cells (T1)

Trail 1 is a straightforward application, where we treat each sample as an independent observation for the estimation of $\boldsymbol{\mathbf { W}}$. We apply the RUV algorithm on the entire matrix $\boldsymbol{\mathbf { Y}}_p$ as if it were a count matrix of a bulk study. The factors of unwanted variation are calculated conditional on the variation that can be explained by the factor of interest, the cell type, and their interaction. The model becomes


(2) \begin{eqnarray*}
E(\log _2(\boldsymbol{\mathbf { Y}}_p)|\boldsymbol{\mathbf { W}},\boldsymbol{\mathbf { V}})&=\boldsymbol{\mathbf { W}} \boldsymbol{\mathbf { \alpha }} + \boldsymbol{\mathbf { V}}\boldsymbol{\mathbf { \gamma }},
\end{eqnarray*}

where $\boldsymbol{\mathbf { V}}_{S \times Q}$ is a matrix with dummy regressors coding for the $Q$ combinations of cell types and levels of the factors of interest (this includes the interactions between cell types and the factor of interest), $\boldsymbol{\mathbf { W}}_{S \times K}$ is the matrix with $K$ hidden factors for the $S$ samples, and and $ \boldsymbol{\mathbf { \gamma }}_{Q \times G}$ are the model parameters. We use the entire count matrix for estimating $\boldsymbol{\mathbf { W}}$, aiming at leveraging as much information as possible. However, the method incorrectly assumes independence between samples.

#### Trail 2: per cell type (T2)

In Trail 2 we propose to use the cell types $t=1,...,T$ for partitioning the matrix $\boldsymbol{\mathbf { Y}}_p$ into $T$ pseudobulk count matrices $\boldsymbol{\mathbf { Y}}_t$ with data from $S_t$ samples for cell type $t$, and proceed to estimate $\boldsymbol{\mathbf { W}}_t$ using only the samples from cell type $t$. This results in the $T$ models


(3) \begin{eqnarray*}
E(\log _2(\boldsymbol{\mathbf { Y}}_t)|\boldsymbol{\mathbf { W}}_t,\boldsymbol{\mathbf { X}})=\boldsymbol{\mathbf { W}}_t \boldsymbol{\mathbf { \alpha }}_t + \boldsymbol{\mathbf { X \beta }}_t.
\end{eqnarray*}

By only using information from the cell type $t$, all samples are independent. However, information from other cell types is ignored.

#### Trail 3: per sample (T3)

We also considered a scenario where the hidden factors are identical across pseudobulk samples from the same single-cell sample, regardless of the cell type. This means that within a sample, for each gene the counts are summed over all cells, whatever the cell type. This assumes that the hidden factors are the same for each cell type, i.e. that the technical noise does not interact with the cell type. This results in the model


(4) \begin{eqnarray*}
E(\log _2(\boldsymbol{\mathbf { Y}}_N)|\boldsymbol{\mathbf { W}}_N,\boldsymbol{\mathbf { X}})&=\boldsymbol{\mathbf { W}}_N \boldsymbol{\mathbf { \alpha }} + \boldsymbol{\mathbf { X \beta }},
\end{eqnarray*}

where $\boldsymbol{\mathbf { Y}}_N$ is the pseudobulk counts matrix with $N$ single-cell samples (rows) and $G$ genes, $\boldsymbol{\mathbf { X}}_{N\times Q}$ contains the $Q$ factors of interest, and $\boldsymbol{\mathbf { W}}_{N\times K}$ is the matrix with $K$ hidden factors.

#### Differential expression analysis

For Trail 1, once $\boldsymbol{\mathbf { W}}$ has been estimated, we use the cell types $t=1,...,T$ to partition the matrices $\boldsymbol{\mathbf { Y}}_p$ and $\boldsymbol{\mathbf { W}}$, respectively, into $T$ pseudobulk count matrices $\boldsymbol{\mathbf { Y}}_t$ and $T$ hidden factors matrices $\boldsymbol{\mathbf { W}}_t$. Each $\boldsymbol{\mathbf { Y}}_t$ matrix has $S_t$ samples from the cell type $t$ (rows) and $G$ columns, each $\boldsymbol{\mathbf { W}}_t$ matrix has $S_t$ rows and $K$ columns. Next we use the model in Equation (5) to identify the differentially expressed genes (DEG) for each cell type $t$. Since one model is fitted per cell type, the interactions are no longer used, and instead of $\boldsymbol{\mathbf { V}}$, we have the matrix $\boldsymbol{\mathbf { X}}$, with $Q$ factors of interest (columns). The model of interest is given by


(5) \begin{eqnarray*}
E(\log _2(\boldsymbol{\mathbf { Y}}_t)|\boldsymbol{\mathbf { W}}_t,\boldsymbol{\mathbf { X}})&=\boldsymbol{\mathbf { W}}_t \boldsymbol{\mathbf { \alpha }}_t + \boldsymbol{\mathbf { X \beta }}_t .
\end{eqnarray*}

For Trail 2, we follow the same approach with the exception that the $\boldsymbol{\mathbf { W}}_t$ matrices do not need to be partitioned from a bigger $\boldsymbol{\mathbf { W}}$ matrix. For Trail 3, we perform the differential expression analysis over $Y_p$, and use the same matrix $\boldsymbol{\mathbf { W}}_N$ for each cell type.

### Addressing the lack of technical replicates in pseudobulk RUV with PBPS

In the absence of (sufficient) technical replicates and plausible negative control genes, factors of unwanted variation cannot be properly estimated. This problem has been tackled by introducing PRPS [8], which are artificial samples generated by taking averages of samples with similar characteristics, accounting for biological and known technical variables; see Appendix B for details. The PRPS approach could also be adopted to estimate the unwanted factors $\boldsymbol{\mathbf { W}}$ by combining RUVIII and any of the trails introduced in Section 2.2. However, in its current form, PRPS still requires a large number of samples. Inspired by PRPS, we suggest an alternative strategy for the removal of unwanted variation by including artificial samples called PBPS.

Let $\mathcal {C}$ represent the set of all cells in the dataset. We propose to create PBPS using the following algorithm:

1. Identify the biological covariates, i.e. the covariates that are associated with variation we are interested in (e.g. the cell type and the factor of interest). We will denote each level of (the combination of) such covariate(s) by $b \in \lbrace 1, \ldots , B\rbrace$. Similarly, identify the known sources of unwanted variation, i.e. the covariates associated with the batch effects (e.g. the batch identifiers), and denote the levels by $l \in \lbrace 1, \ldots , L\rbrace$.
2. Group cells from single-cell samples that share the same values for the biological covariates. Each group will be called a biological subgroup $b$. These sets are denoted as $\mathcal {C}_b$.
3. Within each biological subgroup $b$, compute the average number of cells in single-cell samples, denoted as $\bar{c}_b$.
4. Within each biological subgroup $b$, group cells that share the same value for the known sources of unwanted variation. Denote this set as $\mathcal {C}_{bl}$.
5. Draw $I$ random samples (cells) with replacement of size $\bar{c}_b$ from every set $\mathcal {C}_{bl}$.
6. Pseudobulk the cells from every random sample $i$, with $i=1,...,I$, to obtain the PBPS $s^{\prime }_{bli}$.

**Figure 3.**
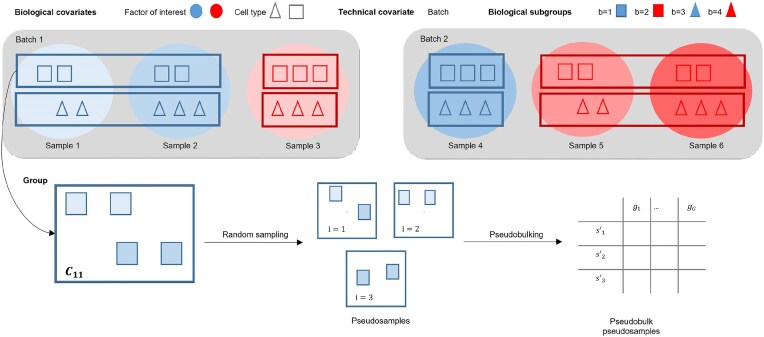
Schematic overview of the process to generate PBPS with six single-cell samples, two biological variables, and one known source of unwanted variation. The first biological covariate, factor of interest, has two levels, and the second biological covariate, cell type, has two levels; therefore $B=2\times 2=4$. There are two batches, therefore $L=2$. The first biological subgroup $\mathcal {C}_1$ is composed by the squared blue cells; it has seven cells from three homogeneous single-cell samples, therefore $\bar{c}_1=2.3$ is the average number of cells per single-cell sample within the set $\mathcal {C}_1$. Of those 7 cells, four were processed in batch 1, and form the set $\mathcal {C}_{11}$: Two in light blue from sample 1, and two in a darker shade from sample 2. We randomly sampled two cells, $I=3$ times; some pseudosamples will have cells from different samples, while others will have cells from only one sample. The cells are then pseudobulked and added to the $\boldsymbol{\mathbf { Y^{\prime }}}_p$ matrix.

At the end, we create a new count matrix $\boldsymbol{\mathbf { Y^{\prime }}}_p$ of size $(S + S^{\prime })\times G$ containing pseudobulk counts from the $S$ original samples and the $S^{\prime }$ new pseudosamples; note that the number of pseudosamples $S^{\prime }$ is determined by the number of random samples $I$ and the number of $\mathcal {C}_{bl}$ sets. The process is further described in Fig. 3.

We make three assumptions in the method. First, we assume that at least one technical variable (source of unwanted variation) is known. Second, we assume that cells with similar biology (same cell type and from homogeneous single-cell samples) and exposed to the same unwanted variation are exchangeable. This means that most PBPS generated by re-sampling and pseudobulking these cells within a group will accurately represent that group. Third, we assume that differences between PBPS from cells with similar biology but exposed to different technical settings are mainly due to unwanted variation. Using this approach, we generate replicates within and across sets, and we are better able to preserve the single-cell information as compared to the PRPS method. PBPS also overcomes the restriction imposed on the PRPS method of needing >3 samples per group, because we can generate multiple pseudosamples using only one single-cell sample per group. When combining RUVIII with PBPS (RUVIII PBPS), all genes can be selected as negative control genes. Using PBPS or PRPS instead of including the known sources of unwanted variation in the model might be preferred when additional unknown sources of unwanted variation are believed to be present in the model.

### Data

The lupus dataset [24] was downloaded from the Chan Zuckerberg CELLxGENE Discover platform [25]. The samples were sequenced in four phases, called processing cohorts. A control subset and a case study subset were created from the data. The control subset has 37 healthy subjects from European and Asian ethnicities between 24 and 28 years old. The case study subset has 85 samples of controls and SLE patients from European and Asian ethnicities between 29 and 34 years old. The samples of both groups were selected to limit the effect of other variables in the gene expression, such as age and gender. Only 8 out of the 11 cell types identified in the dataset were selected for the analyses: CD4^+^ and CD8^+^ T cells (T4 and T8), CD14^+^ classical and CD16^+^ nonclassical monocytes (cM and ncM), conventional and plasmacytoid dendritic cells (cDC and pDC), natural killer cells (NK), and B cells (B). Further detail about the data and the preprocessing is presented in Appendix C.

## Results

We performed an exploratory analysis of the control and case study subsets after upper quartile normalization. To assess unwanted variation in the normalized matrices $\log _2(\boldsymbol{\mathbf { Y}})^{*}$, we used principal component analysis (PCA) plots, averaged silhouette width coefficients (ASW), and relative log-expression (RLE) plots. The metrics are further explained in Appendix E. When the known sources of unwanted variation are not confounded with the factors of interest or other biological covariates, an effective batch correction method should yield low ASW when clustering by known sources of unwanted variation and boxplots with similar dispersions and centred at zero in the RLE plots.

As further explained in Sections 3.1 and 3.2, both the control and the case study subset display a strong processing cohort effect across cell types. Thus, the processing cohort is a known source of unwanted variation and will be used to evaluate how well the combination of trails and RUV methods detect and remove its signal. We also explore the changes in sensitivity and FDR when conducting DEA with and without the unwanted factors from the RUV methods. This paper focuses on the pseudobulk samples from the most abundant cell type in the dataset: CD4 T-cells; however, the effect of the processing cohorts on the gene expression is present in all cell types (Supplementary Figs S2 and S7). Results for every cell type and every RUV method from the *ruv* package [20] are also available in the GitHub repository.

### Unwanted variation in the control subset

**Figure 4.**
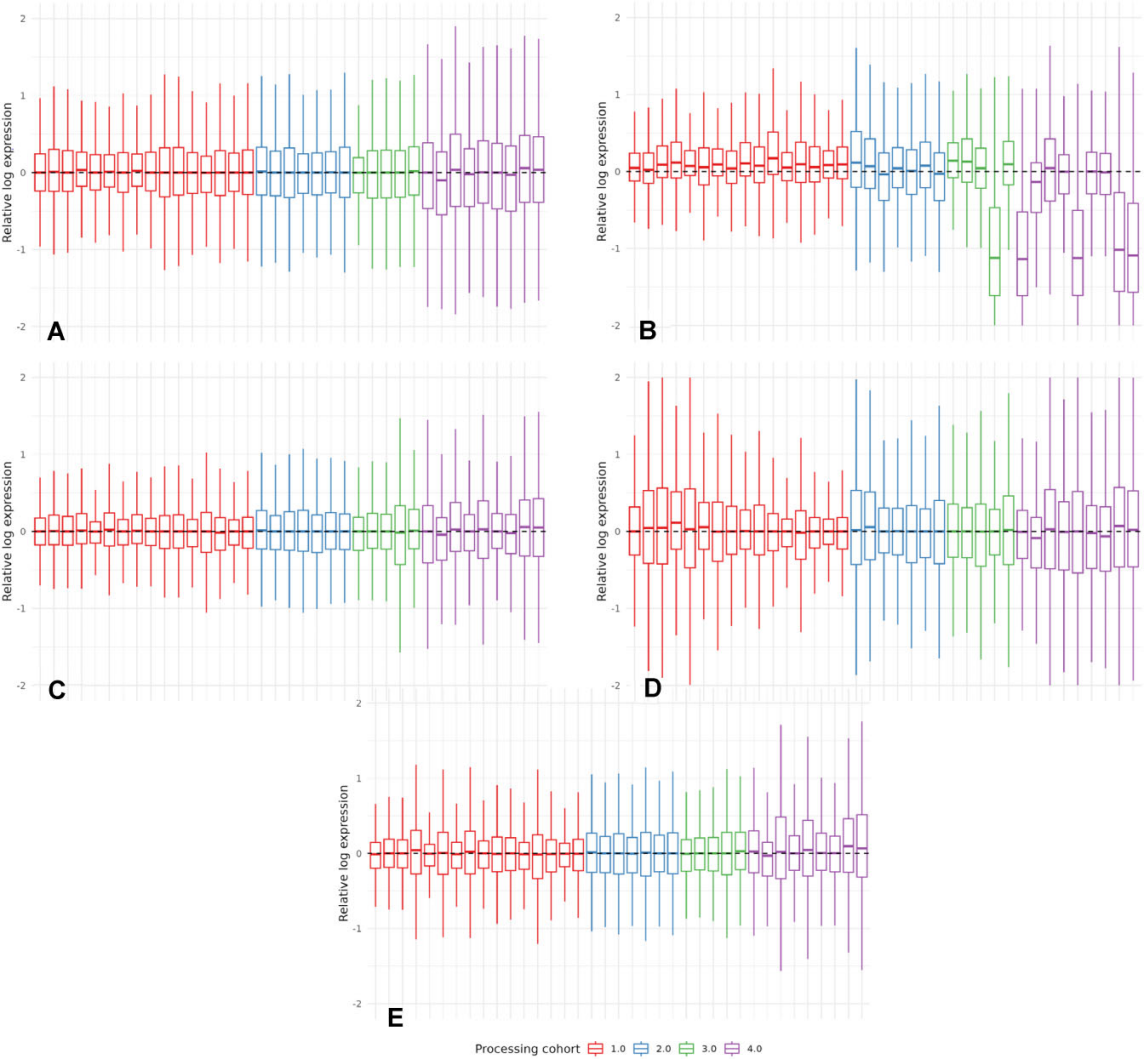
RLE plots of pseudobulk samples from CD4^+^ T cells in the control subset. Boxplots organized by processing cohort. (**A**) After UQ normalization. (**B**) After UQ and RUV2 normalization following Trail 1. (**C**) After UQ and RUV2 normalization following Trail 2. (**D**) After UQ and RUV2 normalization following Trail 3. (**E**) After UQ normalization and processing cohort effect subtraction.

Unwanted variation associated with the processing cohorts is revealed by all metrics applied to the control subset. For instance, the RLE plot in Fig. 4A has notably higher dispersion in processing cohort 4 compared to others. Similarly, the PCA plot reveals clustering by processing cohort (Supplementary Fig. S1A), with an ASW of 0.41 using two principal components (PCs) and 0.33 using 10 PCs. To remove these and possibly other unwanted effects, we evaluate the performance of three RUV methods: RUV2 [3], RUVIII [7], and RUV4 [4], which are all available in the R package *ruv* [20]. Each method is applied using the three trails introduced in Section 2.2 and five ($K=5$) unwanted factors. We compared their performance against the naive UQ model with no information about the unwanted variation and the UQ Batch model, where the known source of unwanted variation is included as a fixed effect. Both models are further described in Appendix D.

#### Removal of unwanted variation in normalized count matrices of pseudobulk studies

All trails managed to eliminate unwanted variation to some extent, as observed in PCA plots of the normalized matrices (Supplementary Fig. S1A), with processing cohort ASW scores below 0.16 across all RUV methods. However, T1 exhibited a strong ethnicity effect, captured in the first principal component (Supplementary Fig. S1B), shifting both the location and magnitude of the RLE distributions for Asian ethnicity samples (Fig. 4B).

T3, where counts are aggregated by single-cell sample, achieved uniform RLE boxplots (Supplementary Fig. S12), but this did not hold in the pseudobulk normalized matrix $\boldsymbol{\mathbf { Y}}^{*}_p$, where counts are aggregated by single-cell sample and cell type. For instance, in the RLE plot for CD4^+^ T cells (Fig. 4D), T3 increased dispersion differences between samples of the same cell type, even within the same processing cohort, suggesting it may introduce unwanted variation. In contrast, T2 (Fig. 4C) and the UQ Batch model (Fig. 4E) yield uniform boxplots, with low dispersion and medians centred around zero.

The results were consistent for other cell types and can be observed in the Supplementary Figs S3–S6 and S8–S11.

To further assess the performance, we computed ASW scores using the first 10 PCs from 100 datasets, each simulating differential expression in $10\%$ of genes between two groups (mock treatments) within the control subset (see Appendix E.2.1 for details). Results (Supplementary Fig. S13) show that the UQ Batch model and T2 combined with RUV2 or RUV4 best reduce processing cohort effects (processing cohort ASW below 0). Conversely, T3 lowers ASW scores for the mock treatment (below 0.6 for all RUV methods), indicating potential removal of biological variability. Overall, T2 and the UQ Batch model provide the most effective normalization.

#### Power and control of the FDR in differential expression analyses of pseudobulk data

We analysed three different scenarios to assess sensitivity and FDR control in differential expression analysis. In the first scenario, we generated 100 datasets by simulating differential expression between two groups (mock treatment A and mock treatment B) in the control subset. The treatments are assigned using a balanced design with respect to the processing cohort. Along with the UQ and RUV models, we use the UQ Batch model without the interaction between ethnicity and the mock treatment to test for differential expression and compute the TPR and FDR. In the second scenario, we follow the same procedure but introduce a confounding effect between the mock treatments and the processing cohorts. In the third scenario, we keep the confounding effect and add an interaction term between ethnicity and mock treatment to the differential expression model to better account for possible differential expression between ethnicities. Further detail about the simulation process is explained in Appendix E.2.1.

**Figure 5.**
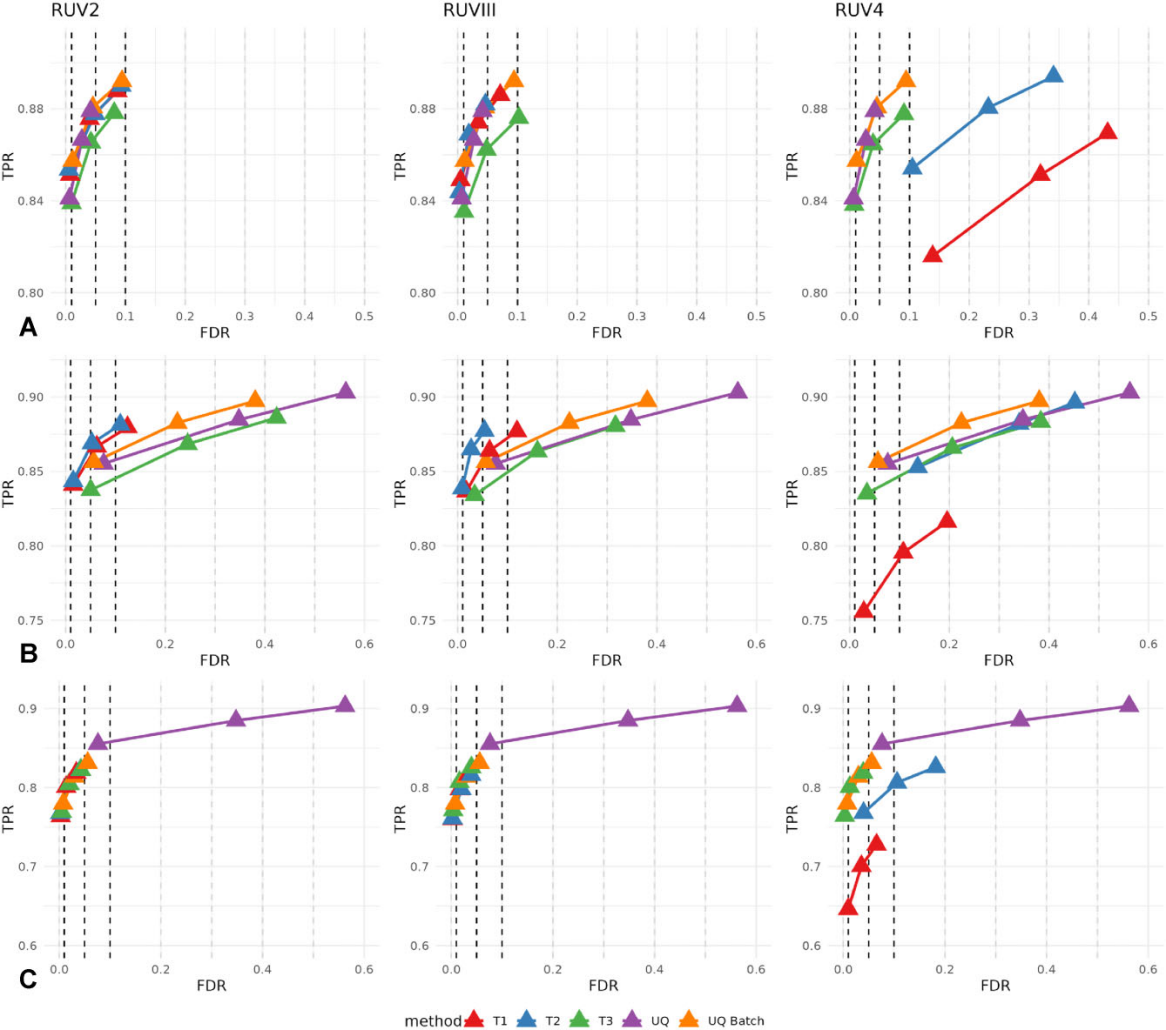
FDR versus TPR at three nominal levels of the FDR ($1\%$, $5\%$, and $10\%$) for combinations of the three RUV methods with either the three trails or with the baseline or gold standard model. Differential expression was simulated 100 times between two mock treatments across CD4^+^ T cell pseudobulk samples from the control subset following three scenarios. (**A**) Individuals assigned using a balanced design. (**B**) Mock treatments confounded with processing cohorts. (**C**) Mock treatments confounded with processing cohorts, an interaction term between ethnicity and mock treatment is included in the model for DEA.

As observed in Fig. 5A, including technical noise information, either through known covariates (UQ Batch) or through latent variables (RUV models), generally improves the TPR, except for T3, where the sensitivity decreases. Particularly in the RUV2 model, the T1 and T2 trails achieved a performance similar to the UQ Batch model, without explicit information about the processing cohorts. In contrast, the same trails in the RUV4 model inflated the FDR. The impact of the confounding factor between processing cohorts and mock treatments is evident in Fig. 5B, where the UQ model exhibits inflated FDR. Including processing cohort information (UQ Batch) does not fully correct the inflated FDR. RUV4 also fails to correct the FDR and reduces the TPR when using T1. In the presence of confounding effects, effective FDR control is achieved using either T1 or T2 with the RUV2 or RUVIII models, even when the DEA model is misspecified. UQ Batch, however, is only effective with a correctly specified model (Fig. 5C). Note that the control subset was selected to minimize the influence of other variables such as age and sex. Nevertheless, in practice, the RUV2 and RUVIII models are expected to outperform UQ Batch when multiple sources of unwanted variation are present. Overall, T2 was the best-performing trail.

#### Leveraging PBPS to improve RUVIII performance

The number of technical replicates in the control subset is unbalanced across processing cohorts. As observed in Supplementary Fig. S14A, processing cohorts 2 and 3 have the larger numbers, while there are no technical replicates in processing cohort 4. This situation limits RUVIII, because not all unwanted variation is captured by the differences between the technical replicates. For instance, in Supplementary Fig. S15, the silhouette width of samples processed in processing cohort 4 is higher, with four out of the nine samples having an SW larger than 0.5 and a total processing cohort ASW of 0.17.

To overcome design limitations when using the RUVIII method, we generated PBPS using the single-cell RNA sequencing dataset from the control subset, three biological covariates (mock treatment, ethnicity, and cell type), and one known source of unwanted variation (processing cohort). We compare the results of the following models: the RUVIII standard implementation (RUVIII), the RUVIII method with PBPS (RUVIII PBPS), the RUVIII method with explicit processing cohort information (RUVIII Batch), and the UQ Batch model. For all models using RUVIII, we followed trail 2.

Compared to the normalized matrix (see Supplementary Fig. S16), we observed that the three alternatives to the standard RUVIII method decreased the processing cohort ASW using 2 PCs from 0.17 to 0.06 (RUVIII PBPS), $-0.16$ (RUVIII Batch), and $-0.07$ (UQ Batch). In the control subset, the estimated unwanted factors show a moderate association with the processing cohort ($R^2 = 0.55$). However, if this association were stronger in practice (e.g. $R^2$ above 0.7 [26]), the model could produce unstable estimates. In such cases, removing these effects during normalization, as done in RUVIII Batch, might unintentionally introduce additional noise linked to those variables.

**Figure 6.**
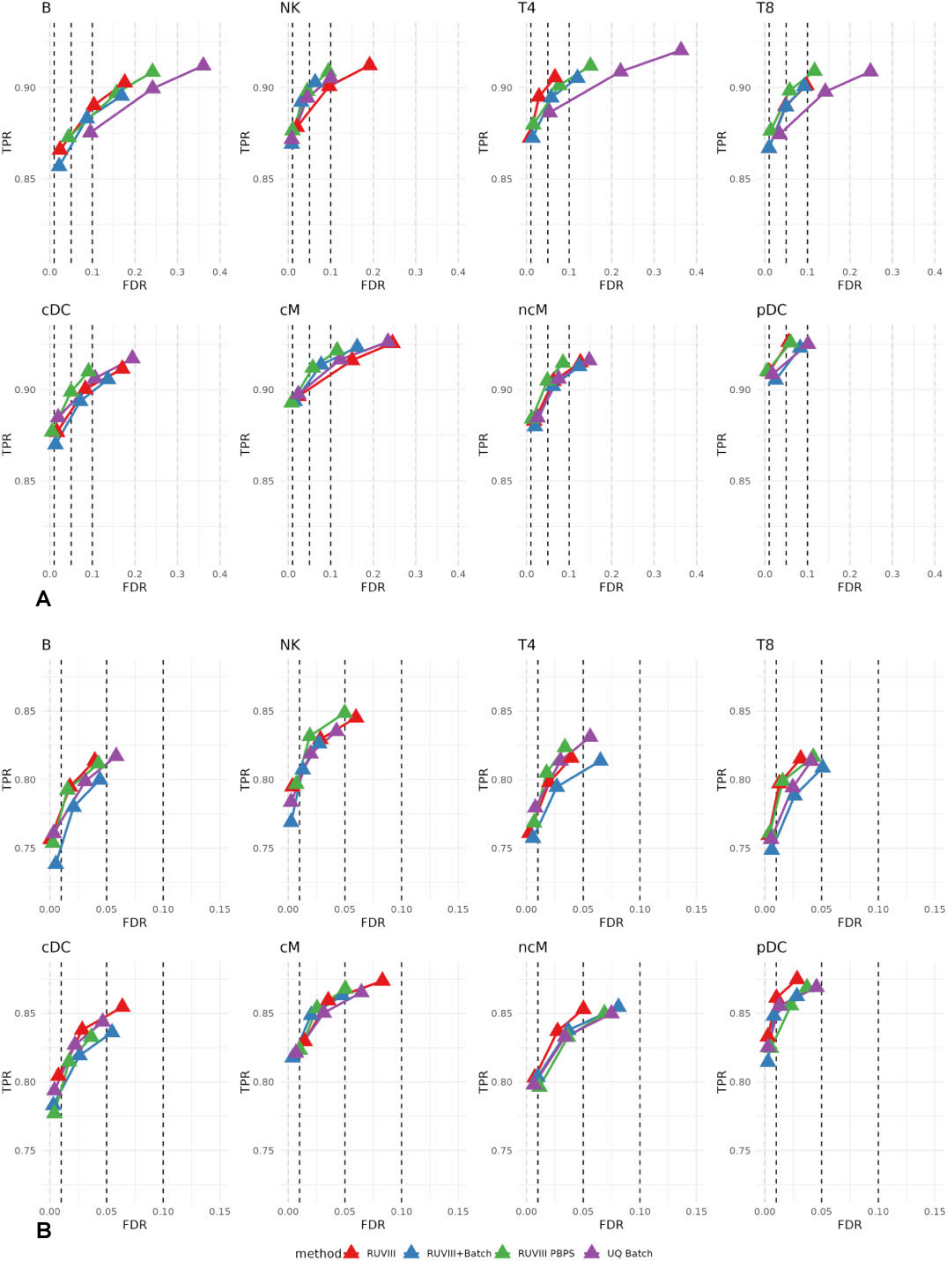
FDR versus TPR at nominal FDR levels of $1\%$, $5\%$, and $10\%$ for the RUVIII, RUVIII PBPS, RUVIII Batch, and UQ Batch models. Differential expression was simulated per cell type 100 times between two mock treatments across pseudobulk samples from the control subset following two scenarios. (**A**) Mock treatments confounded with processing cohorts. (**B**) Mock treatments confounded with processing cohorts, with an interaction term between ethnicity and mock treatment included in the DEA model.

Regarding the control of the FDR in differential expression analyses, we analysed the second and third scenarios from Section 3.1.2 for all cell types (see Fig. 6). The first scenario was not included since RUV methods provided no added value to perform DEA in the control subset when the mock treatment groups were balanced across processing cohorts. When no interaction effect is considered, as observed in Fig. 6A, the inclusion of the hidden unwanted factors computed using RUVIII PBPS sustains or increases the high TPR of the standard RUVIII method for most of the cell types. Compared with the UQ Batch model, it keeps the true FDR values closer to the nominal levels. On the other hand, as observed in Fig. 6B, adding the interaction effect between ethnicity and mock treatment brings the true FDR values of the UQ Batch model closer to the nominal levels, and, in general, similar TPR and FDR values are achieved with any model.

### Case study

The case study subset, further detailed in Appendix C, consists of two subject groups based on disease status: systemic lupus erythematosus (SLE) patients and healthy controls. The primary objective is to identify DEG between these conditions. We will use 100 genes from the interferon-stimulated genes (ISG) signature [27] that were picked up in previous lupus studies [24, 28] as a proxy for sensitivity.

**Figure 7.**
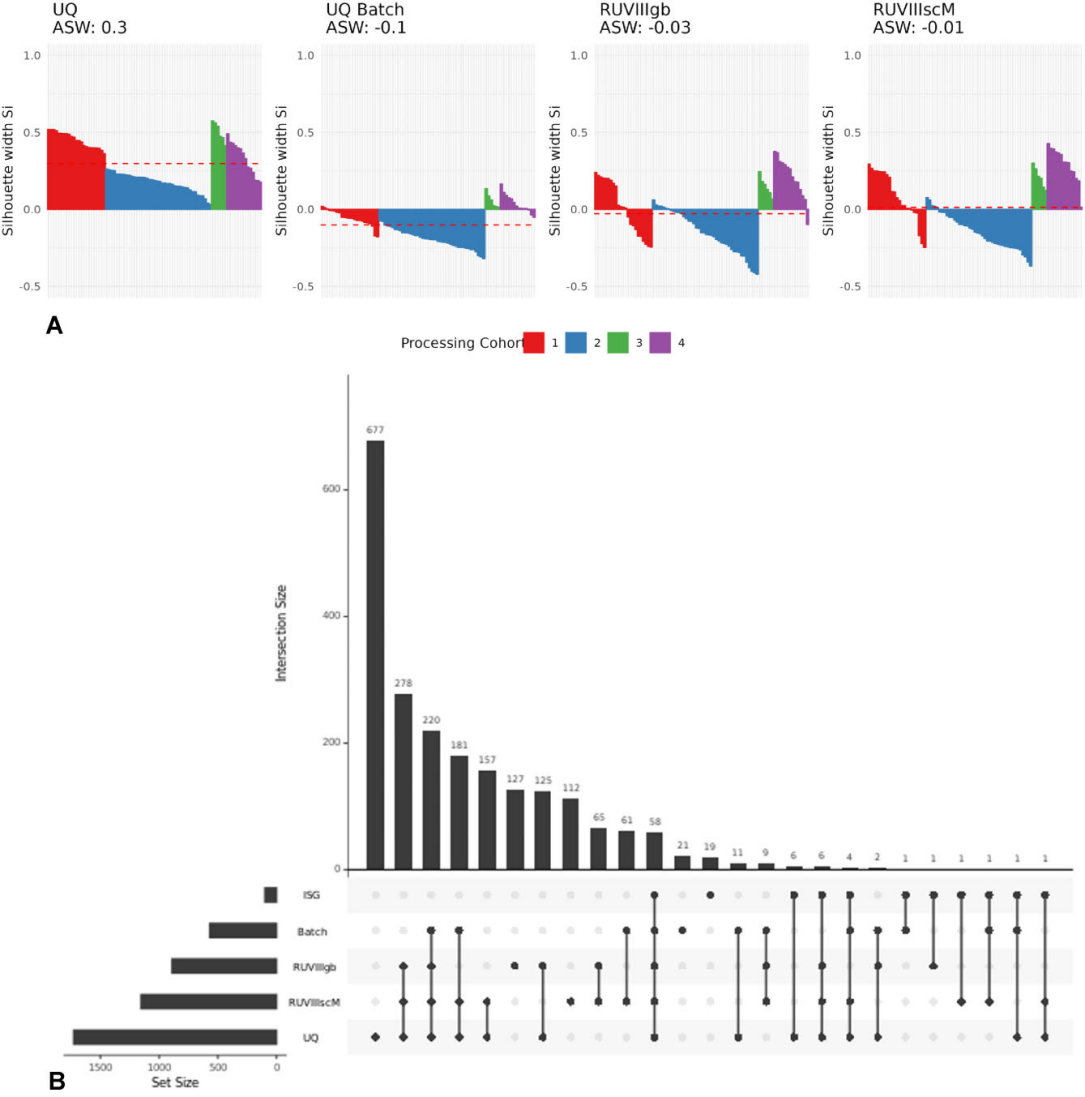
**A**) Processing cohort silhouette width scores of CD4^+^ T samples in the lupus case study subset using the UQ Batch, RUVIIIgb, and RUVIIIscM methods; the dashed lines indicate the ASW. (**B**) UpSet plot of DEGs in CD4^+^ T samples from the lupus case study subset, comparing the UQ, UQ Batch, RUVIIIgb, and RUVIIIscM models with the ISG signature.

The exploratory data analysis revealed a batch effect of the processing cohort. Moreover, processing cohorts 1 and 3 only contain healthy subjects, and cohort 3 only has European subjects (Supplementary Fig. S14B). Additionally, samples from processing cohorts 2 and 4 also exhibit positive processing cohort silhouette width coefficients (Fig. 7A), indicating clustering effects even in cohorts with samples from both conditions.

A naive DEA, ignoring the unwanted variation in the data (UQ model from Appendix D), detected a large number of DEGs. Specifically, in the CD4^+^ T samples, we observed 1727 DEGs detected at a $1\%$ FDR; however, 1012 of these genes were also differentially expressed across the processing cohorts in the control subset. Moreover, a gene set enrichment analysis (GSEA) with all DEGs identified 35 enriched sets, a small number considering the 246 enriched sets obtained with the 100 genes from the ISG signature. Worse results are achieved in other cell types; for instance, the classical monocytes had 3883 DEGs but zero enriched sets. These results, along with simulations in Section 3.1.2, suggest an inflated FDR, a possible product of the confounding effect between the processing cohorts and the disease status.

#### Discordance between RUVIII results when using different sets of negative control genes

In the analyses presented in Section 3.1, we ensured that differential expression was not simulated for the set of genes designated as negative controls, aiming to use true negative controls. However, identifying genes with truly constant expression across samples remains challenging.

We selected two sets of negative control genes, introduced in Appendix C.2, and applied the RUVIII method with $K=3$ unwanted factors to create the RUVIIIgb and the RUVIIIscM models. Additionally, we fitted the UQ Batch model. We included the interaction term between ethnicity and disease status in the UQ Batch and RUVIII models.

The ASW plots in Fig. 7A show that the RUVIII methods failed to remove the batch effect in processing cohort 4. On the other hand, the Batch UQ model resulted in negative silhouette width (SW) coefficients in processing cohort 1, which is mainly composed of healthy Europeans, where clustering patterns are expected.

To assess the relationship between the unwanted factors estimated by the RUVIII models and the processing cohorts, we performed a Kruskal–Wallis test. In the RUVIIIscM model, all unwanted factors across all cell types were significantly associated with processing cohorts (Supplementary Table S1). In the RUVIIIgb model, only the third unwanted factor in T cells (T4 and T8) and classical monocytes (cM) did not show a significant association ($\alpha =0.05$) (Supplementary Table S2).

We performed a DEA and compared the DEGs from each model at the nominal $1\%$ FDR level with the 100 genes from the ISG signature [27]. Six genes from the signature were not detected by any method in any cell type: TMEM140, IFNGR2, FITM1, GADD45B, IFNAR1, and IFNAR2. The UQ batch model detected 82 genes, the RUVIIIsc model detected 86 genes, the RUVIIIgb model detected the same 86 genes plus two more, and the UQ model detected 92 genes (Supplementary Table S3).

From the UpSet plots we see that most of the signature genes identified by all methods are in the monocyte samples (cM = 77, ncM = 50) and the T samples (T4 = 58, T8 = 45). The RUVIII methods identified different sets of DEGs. For instance, in Fig. 7B, we observed 127 genes exclusively detected by RUVIIIgb and 112 genes exclusively detected by RUVIIIscM. Large differences in DEG between RUVIIIgb and RUVIIIscM were present across all cell types (Supplementary Figs S17–S22). RUVIIIscM detected more DEG in samples from B, natural killers, T, and dendritic cells, while RUVIIIbg detected more DEG in samples from classical and non-classical monocyte cells.

Finally, we performed a GSEA with the DEGs of every method ($1\%$ FDR level) and a GSEA with the ISG signature. We focused the analyses on the cell types with the most DEGs from the ISG signature: the T cells and the monocytes. Despite detecting the highest number of DEGs, the UQ model had no enriched gene sets in classical monocyte samples. The RUVIIIscM model had the highest overlap with the ISG signature gene enrichment sets in monocyte samples (cM and ncM), while RUVIIIgb model had a higher overlap in T samples (Supplementary Figs S23–S26). We also looked into the sets identified by only one RUVIII method: T4 had 19, T8 had 30, cM had 21, and ncM had 63 unique sets.

#### RUVIII PBPS performance

We compared the UQ and UQ Batch models to an RUVIII model with $K=3$ unwanted factors, using no negative control genes and additional negative control samples generated with PBPS (RUVIII PBPS). We defined groups of cells using the combination of levels from the known source of unwanted variation: processing cohort and the biological covariates cell type, disease status, and ethnicity. Ten PBPS were created per group, resulting in a total of 880 PBPS.

**Figure 8.**
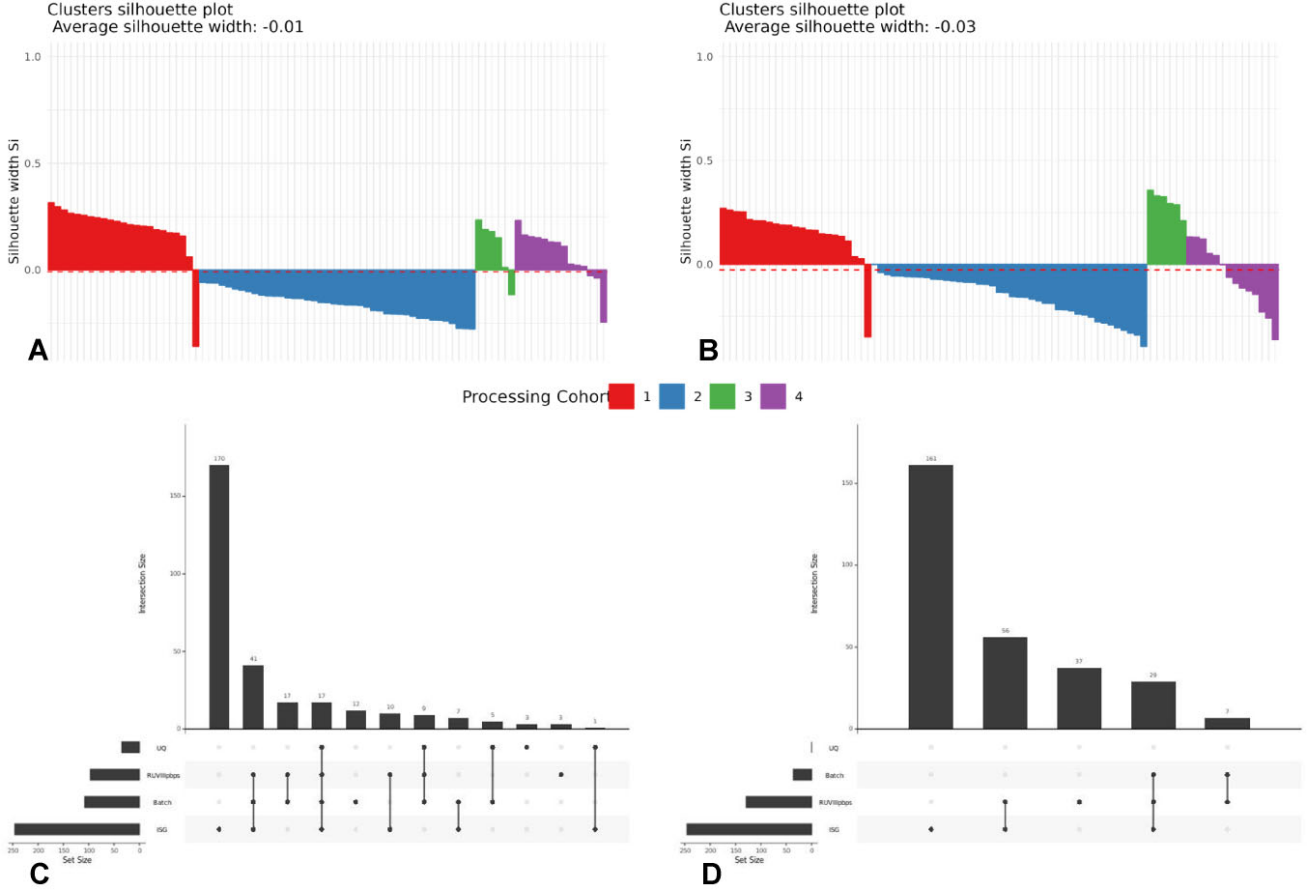
**A B**) Processing cohort silhouette width scores for CD4^+^ T and classical monocyte samples in the lupus case study subset using the RUVIII PBPS model; the dashed lines indicate the ASW. (**C, D**) UpSet plots of enriched gene sets in CD4^+^ T and classical monocyte samples, comparing UQ, UQ Batch, and RUVIII PBPS models with the ISG signature.

The ASW plots in Fig. 8A and B show that the RUVIII PBPS method removed unwanted variation in processing cohort 2 and decreased the SW coefficients in cohort 4. The positive silhouette coefficients in cohorts 1 and 3 are expected since these samples are all from healthy European subjects, i.e. have similar biology. In contrast, the only sample from an Asian subject has a negative silhouette value. This pattern is consistent across cell types (Supplementary Figs S27–S33).

The Kruskal–Wallis test confirmed a significant association ($\alpha =0.05$) between the unwanted factors from the RUVIII PBPS model and the processing cohorts for all cell types (Supplementary Table S4).

We repeated the DEA from Section 3.2.1. On average, the RUVIII PBPS model detected fewer DEGs than the UQ Batch model, but the overlap with the ISG signature genes remained similar: PBPS identified 82 signature genes in at least one cell type, of which 81 were also detected by the UQ Batch model (Supplementary Table S5).

Figure 8C and D show that in the GSEA, the RUVIII PBPS model identified more enriched gene sets in common with the ISG signature than the UQ or UQ Batch models. This pattern was consistent across all cell types except for natural killer cells, where the UQ Batch model had three additional enriched sets in common with the ISG signature (Supplementary Figs S34 and S35). Overall, the RUVIII PBPS model removed unwanted variation while preserving biological signal, showing strong overlap with the ISG signature. It detected fewer DEGs but matched or exceeded the UQ models in enriched gene sets, confirming its robustness.

## Discussion

The benchmarking measures used in the paper (PCA plots, silhouette width coefficients, and RLE plots) identified the principal technical effect in the dataset: the processing cohort. These methods are widely used to recognize technical effects in batch correction studies [2, 3, 5, 7, 10, 11, 29]. We emphasize the importance of routinely incorporating these tools in pipelines to detect and correct unwanted variation prior to downstream analyses.

For pseudobulk data from single-cell RNA sequencing, we recommend using RUV methods following Trail 2, i.e. estimating factors of unwanted variation for each cell type separately. Using all pseudobulk samples as in Trail 1 introduces differences in the relative log expression of samples with different biological information. Additionally, although cells from the same single-cell sample are exposed to similar technical effects, pseudobulking the data without considering cell types, as in Trail 3, reduces the strength of the biological signal in gene expression measurements. This occurs when using any of the evaluated RUV methods (RUV2, RUVIII, and RUV4).

Among the RUV methods available in the *ruv* R package, RUV4 is not recommended under the current settings due to its low TPR and inflated FDR. The method has usually been used with a higher number of hidden unwanted factors [4], but that configuration also reduces the degrees of freedom in the differential expression model. Correcting for systematic noise with known sources of unwanted variation is sufficient when no confounding effects are present, as in Fig. 5A. However, in practice, the sources of unwanted variation and/or the presence of confounding factors are unknown, as in Fig. 5B and C. Therefore, the use of RUV methods is generally recommended.

In Section 3.2.1 we tested two RUVIII models using different sets of negative control genes [18, 30] to estimate the unwanted factors. These models identified different DEGs, suggesting that at least one set was incorrectly specified for human PBMC samples. The effects of negative control gene misspecification were further explored in Appendix F. It can decrease the TPR and inflate the FDR, especially in the absence of negative control samples.

We proposed the creation of PBPS to remove unwanted variation in datasets that lack negative control samples. Generating PBPS requires fewer original samples than PRPS [8], and it can be used in RUVIII models using all genes as negative control genes.

The simulation results in Section 3.2.2 show that in misspecified models, RUVIII PBPS controls the FDR better than models that directly include the information about known sources of unwanted variation (UQ Batch and RUVIII Batch) or RUVIII models that only use the available negative control samples. In correctly specified models, RUVIII PBPS performs similarly to UQ Batch and RUVIII Batch. The unwanted factors produced by RUVIII had a moderate correlation with the known source of unwanted variation. However, multicollinearity problems might arise when said correlation is higher; therefore, the use of RUVIII Batch models is discouraged. Additionally, in the case study, the RUVIII PBPS model identified more enriched gene sets related to the reference signature [27] than the other models while detecting fewer DEGs in most cell types. However, the RUVIII PBPS method needs biological and technical information, whereas other RUV methods do not.

The number of PBPS used was arbitrary but could be optimized by analyzing the variance within groups defined by the biological covariates and known sources of unwanted variation. Furthermore, the RUVIII PBPS $\boldsymbol{\mathbf { W}}$ factors could also be used as a gold standard to test negative control gene sets in a dataset. For instance, by projecting the unwanted factors generated by each set onto the subspace of the unwanted factors of RUVIII PBPS with no negative control genes and using a measurement such as Hoteling’s trace or Wilk’s lambda to assess the association between the two sets.

## Funding

No external funding.

## Supplementary Material

lqaf179_Supplemental_File

## Data Availability

Processed data files are available at DOI:10.5281/zenodo.15341158. All code used for simulations and data analysis is available at https://doi.org/10.6084/m9.figshare.29425226.v1 and https://github.com/esprietol/ruv-pseudobulk. The R package to create PBPS is available at https://github.com/esprietol/ruvPBPS and is currently under preparation for submission to Bioconductor.
