# Supplementary material for: Removal of unwanted variation in pseudobulk analysis of single-cell RNA sequencing data and the leveraging of pseudoreplicates
Source: NAR Genom Bioinform. 2025 Dec 8;7(4):lqaf179. doi: 10.1093/nargab/lqaf179 (PMC12684399; doi:10.1093/nargab/lqaf179)
Supplement: lqaf179_Supplemental_File [file lqaf179_supplemental_file.pdf]

Removal of unwanted variation in pseudobulk analysis of single-cell RNA sequencing data and the leveraging of pseudoreplicates

Sofía Prieto León[1], Ewoud De Troyer[2], Helena Geys[1,2], Koen Van den Berge[2] and Olivier Thas[1,3,4]
[1] Data Science Institute and I-BioStat, Hasselt University, Hasselt, 3500, Belgium; [2] Statistics and Decision Sciences, Johnson & Johnson Innovative Medicine, Beerse, 2340, Belgium; [3] Department of Mathematics, Computer Science and Statistics, Ghent University, Ghent, 9000, Belgium; [4] National Institute for Applied Statistics Research Australia (NIASRA), University of Wollongong, Wollongong, New South Wales, 2522, Australia

October 11, 2025

Appendix

A RUV methodologies

[4] assumed that the log2-expression matrix $\log_2(\mathbf{Y})_{N\times G}$ could be analysed with a linear regression model by a set of factors of interest, $\mathbf{X}_{N\times Q}$, and a set of unwanted factors $\mathbf{W}_{N\times K}$, often unknown. N is the number of samples, G the number of genes, Q the number of factors of interest, and K the number of unwanted hidden factors. The model can be written as

$$\log_2(\mathbf{Y}) = \mathbf{W}\boldsymbol{\alpha} + \mathbf{X}\boldsymbol{\beta} + \boldsymbol{\epsilon} \tag{1}$$

In which the parameters $\boldsymbol{\beta}_{Q\times G}$ and $\boldsymbol{\alpha}_{K\times G}$ describe the biological and technical effects, and $\boldsymbol{\epsilon}$ is the model's errors matrix.

To perform a RUV normalisation, we focus on the estimation of the unwanted factors $\mathbf{W}$, and the Differential Expression Analysis (DEA). The latter is often performed using the model in the equation (1). We will further discuss the unwanted factors estimation under different methods.

A.1 RUV2

One way of estimating the unwanted factors is by assuming the existence of a subset of genes with non-differential expression across samples (low variance), called negative control genes. The estimation method of $\mathbf{W}$ presented by [4] uses the linear regression model.

To denote the information related to the negative control genes, we use the sub-index $\mathcal{G}$; for instance, $Y_{\mathcal{G}}$ is the matrix with counts only from the negative control genes.

The assumption of no differential expression implies that the effect of the factor of interest $\mathbf{X}$ over $\log_2(\mathbf{Y}_{\mathcal{G}})$ is zero ($\boldsymbol{\beta}_{\mathcal{G}} = 0$). Then, the model in the equation (1) applied to only the negative control genes becomes

$$\log_2(\mathbf{Y}_{\mathcal{G}}) = \mathbf{W}\boldsymbol{\alpha}_{\mathcal{G}} + \mathbf{X}\boldsymbol{\beta}_{\mathcal{G}} + \boldsymbol{\epsilon}_{\mathcal{G}} = \mathbf{W}\boldsymbol{\alpha}_{\mathcal{G}} + \boldsymbol{\epsilon}_{\mathcal{G}} \tag{2}$$

Next, we proceed to approximate the product of the unwanted hidden factors $\mathbf{W}$ and the parameters $\boldsymbol{\alpha}_{\mathcal{G}}$ using the centred log counts matrix; in other words, $\log_2(\mathbf{Y}_{\mathcal{G}})^* \approx \widehat{\mathbf{W}\boldsymbol{\alpha}_{\mathcal{G}}}$. To find separate estimates of $\mathbf{W}$ and $\boldsymbol{\alpha}_{\mathcal{G}}$, we apply an Exploratory Factor Analysis (EFA), for instance, via Truncated Singular Value Decomposition (TSVD), using the first K singular values.

$$\widehat{\mathbf{W}\boldsymbol{\alpha}_{\mathcal{G}}} = \mathbf{U}\boldsymbol{\lambda}_K\mathbf{V}^T \tag{3}$$

Where $\mathbf{U}\boldsymbol{\lambda}_K$ is the estimate of $\mathbf{W}$, and V^T is the estimate of $\boldsymbol{\alpha}_{\mathcal{G}}$

Despite it is not advised to work with the normalised matrix $\tilde{\mathbf{Y}}$, for diagnostic purposes the matrix is computed as follows:

$$\widehat{\boldsymbol{\alpha}} = (\widehat{\mathbf{W}}^T\widehat{\mathbf{W}})^{-1}\widehat{\mathbf{W}}^T \log_2(\mathbf{Y}) \tag{4}$$

$$\widehat{\log_2(\mathbf{Y})} = \log_2(\mathbf{Y}) - \widehat{\mathbf{W}\boldsymbol{\alpha}} \tag{5}$$

A.2 RUV4

Inspired by Surrogate Variable Analysis (SVA)[7] and RUV2, RUV4 [3] uses the linear regression model and projects the log2-expressions matrix $\log_2(\mathbf{Y})$ on the column space's orthogonal complement ($\mathbf{R}_X$) of the factors of interest to remove the biological effects before estimating the unwanted factors.

Let $P_X = \mathbf{X}(\mathbf{X}^T\mathbf{X})^{-1}\mathbf{X}^T$ be the projection matrix of $\mathbf{X}$, and $R_X = (\mathbf{I} - P_X)$ the residual operator, such that $R_X \log_2(\mathbf{Y})$ is the residual matrix of the model without unwanted hidden factors $\log_2(\mathbf{Y}) = \mathbf{X}\boldsymbol{\beta} + \boldsymbol{\epsilon}$. Then, if we applied the residual operator in the model from equation 1, we obtain

$$R_X \log_2(\mathbf{Y}) = R_X\mathbf{X}\boldsymbol{\beta} + R_X\mathbf{W}\boldsymbol{\alpha} + R_x\boldsymbol{\epsilon} = \mathbf{W}_0\boldsymbol{\alpha} + R_X\boldsymbol{\epsilon} \tag{6}$$

With $\mathbf{W}_0 = R_X\mathbf{W}$. In the next step of the algorithm, we approximate $\mathbf{W}_0\boldsymbol{\alpha}$ using $R_X \log_2(\mathbf{Y})$, and find the individual estimate of $\mathbf{W}_0$ via EFA, particularly TSVD

$$R_X \log_2(\mathbf{Y}) \approx \mathbf{U}\boldsymbol{\lambda}_k\mathbf{V}^T \tag{7}$$

$$\widehat{\mathbf{W}_0} = \mathbf{U}\boldsymbol{\lambda}_k \tag{8}$$

However, RUV4 is interested in the unwanted factors $\mathbf{W}$, not $\mathbf{W}_0 = R_XW$. Therefore, once $\mathbf{W}_0$ is estimated, we use the negative control genes to find an estimate for $\mathbf{W}$. To do so, first we introduce the partial regression coefficients of A on B, as $b_{AB} \equiv (B^TB)^{-1}B^TA$ and the partial regression coefficients of A on B adjusted by C as $b_{AB.C} \equiv (B^TR_CB)^{-1}B^TR_CA$. The first set of coefficients corresponds to the estimates of the model $A = Bb + \epsilon$; and the second to the model $R_CA = R_CBb + R_C\epsilon$.

Then, we rewrite $\mathbf{W}_0$

$$\mathbf{W}_0 = R_X\mathbf{W} = \mathbf{W} - \mathbf{X}(\mathbf{X}^T\mathbf{X})^{-1}\mathbf{X}^T\mathbf{W} = \mathbf{W} - \mathbf{X}b_{WX} \tag{9}$$

From the previous expression, we define $\mathbf{W} = \mathbf{W}_0 + \mathbf{X}b_{WX}$.

To find b_{WX} we make use of the identity

$$b_{\log_2(Y)_{\mathcal{G}}X} = b_{\log_2(Y)_{\mathcal{G}}X.W} + b_{WX}b_{\log_2(Y)_{\mathcal{G}}W.X} \tag{10}$$

Note that $b_{\log_2(Y)_{\mathcal{G}}X.W} \approx \boldsymbol{\beta}_{\mathcal{G}} = 0$, and $b_{\log_2(Y)_{\mathcal{G}}W.X} \approx \boldsymbol{\alpha}_{\mathcal{G}}$. Therefore, solving the Equation (10) we obtain

$$b_{WX} \approx b_{\log_2(Y)_{\mathcal{G}}X}\widehat{\boldsymbol{\alpha}}_{\mathcal{G}}^T(\widehat{\boldsymbol{\alpha}}_{\mathcal{G}}\widehat{\boldsymbol{\alpha}}_{\mathcal{G}}^T)^{-1} \tag{11}$$

and, replacing b_{WX} in Equation (9)

$$\widehat{\mathbf{W}} = \widehat{\mathbf{W}}_0 + \mathbf{X}b_{\log_2(Y)_{\mathcal{G}}X}\widehat{\boldsymbol{\alpha}}_{\mathcal{G}}^T(\widehat{\boldsymbol{\alpha}}_{\mathcal{G}}\widehat{\boldsymbol{\alpha}}_{\mathcal{G}}^T)^{-1} \tag{12}$$

A.3 RUVIII

RUVIII [12] combines the use of technical replicates and negative control genes. The method relies on the assumption of no correlation between the unwanted factors and the variables of interest, the existence of a negative control gene set and technical replicates.

Let us first introduce $\mathbf{M}_{N\times J}$ as a mapping matrix, with N the number of samples and J the number of subjects. The element (n, j) of $\mathbf{M}$ is one if the nth sample is a technical replicate of the jth subject and zero otherwise.

Similar to RUV4, RUVIII uses a residual operator $R_M = I - \mathbf{M}(\mathbf{M}^T\mathbf{M})^{-1}\mathbf{M}^T$ and applies it to the model from equation (1)

$$R_M \log_2(\mathbf{Y}) = R_M\mathbf{X}\boldsymbol{\beta} + R_M\mathbf{W}\boldsymbol{\alpha} + R_M\boldsymbol{\epsilon} = \mathbf{W}_0\boldsymbol{\alpha} + R_M\boldsymbol{\epsilon} \tag{13}$$

Here, $R_{M_{N\times N}}$ is the residual operator of $\mathbf{M}$. $\mathbf{X}_{N\times Q} = \mathbf{M}\boldsymbol{X}$ is the matrix with biological variables at a sample level, while $\boldsymbol{X}_{J\times Q}$ is the matrix with biological variables at a single-cell sample level. Note that $R_M\mathbf{X} = \mathbf{M}\boldsymbol{X} - \mathbf{M}(\mathbf{M}^T\mathbf{M})^{-1}\mathbf{M}^T\mathbf{M}\boldsymbol{X} = 0$.

Then, analogous to RUV4, we approximate $\mathbf{W}_0\boldsymbol{\alpha}$ with $R_M \log_2(\mathbf{Y})$ and find the estimate of $\boldsymbol{\alpha}$ via TSVD

$$R_M \log_2(\mathbf{Y}) = \mathbf{U}\boldsymbol{\lambda}_k\mathbf{V}^T \tag{14}$$

$$\widehat{\boldsymbol{\alpha}} = \mathbf{V}^T \tag{15}$$

To estimate $\mathbf{W}$, the authors proceed to use the information of exclusively the control genes

$$\widehat{\mathbf{W}} = \log_2(\mathbf{Y})_{\mathcal{G}}\widehat{\boldsymbol{\alpha}}_{\mathcal{G}}^T(\widehat{\boldsymbol{\alpha}}_{\mathcal{G}}\widehat{\boldsymbol{\alpha}}_{\mathcal{G}}^T)^{-1} \tag{16}$$

And finally, the matrix $\log_2(\mathbf{Y})$ is adjusted:

$$\widehat{\log_2(\mathbf{Y})} = \log_2(\mathbf{Y}) - \widehat{\mathbf{W}\boldsymbol{\alpha}} \tag{17}$$

The RUVIII method is designed to normalise the data, and not to perform a DEA. The $\mathbf{W}$ factors are not orthogonal, and it doesn't require information about covariates or the factor(s) of interest. Additionally, to capture the unwanted variation, the technical replicates must be distributed across the batches or other types of unwanted sources of effects we intend to remove.

B The concept of pseudoreplicates pseudosamples (PRPS)

RUVIII pseudoreplicates pseudosamples (PRPS) is strategy to implement the RUVIII method in the absence of (sufficient) technical replicates [13] .

To use PRPS, we must first identify the major biological signals present in the data and then create biological subgroups with homogeneous samples concerning said information. In the datasets presented in Appendix C.1.1, ignoring the cell type information, the biological populations are Europeans with mock treatment A, Europeans with mock treatment B, and Asians with mock treatment B. Then, we must have information about the possible sources of unwanted variation and have the samples within each biological subgroup distributed across it. The samples from the same sub-group and with similar conditions regarding the unwanted variation are defined as groups. In the dataset from this paper, the main sources of unwanted variation are the processing cohorts; one group is, for instance, samples from Europeans with mock treatment A, analysed in processing cohort 1.

The procedure to create the PRPS is the following:

1. Define groups of at least 3 roughly homogeneous samples concerning unwanted variation and biology.

2. In each group, average the gene expression of each gene.

3. The averages of groups from the same biological subgroups are considered pseudoreplicates of one pseudosample.

In particular, assume we have a count matrix $\mathbf{Y}$ with G genes and N pseudobulk samples distributed across L batches and B biological subgroups. We will use Y_n to refer to the nth row of $\mathbf{Y}$, which contains the counts of the G genes from sample n, and Y'_{bl} to refer to vector with the averages of the G genes that form the lth pseudo-replicate, from batch l ,in the biological subgroup b.

Each element of Y'_{bl} is computed as:

$$y'_{blg} = \frac{\sum_{n=1}^{N} Y_n I_{bl}}{\sum_{n=1}^{N} I_{bl}} \tag{18}$$

where $I_{bl} = 1$ if n belongs to the batch l and the biological subgroup b and 0 otherwise.

At the end, a new count matrix Y' containing both the samples y_n and the pseudoreplicates y'_{bl} is created, of size $(N + N') \times G$, where N' is the number of pseudoreplicates that we created. When all possible groups have at least three assays, then $N' = BL$.

C Datasets

C.1 The Lupus dataset

The Lupus dataset [14] was downloaded from the Chan Zuckerberg CELL by GENE Discover (CZ CELLxGENE Discover) platform [15] using the publication title. It contains 355 single-cell RNA sequencing (scRNA-seq) samples with 1,263,676 peripheral blood mononuclear cells (PBMCs). The samples belong to 162 systemic lupus erythematosus (SLE) cases in the California Lupus Epidemiological Study (CLUES) cohort and 95 healthy controls, 49 from the UCSF Rheumatology Clinic and 46 from the Immune Variation Project (ImmVar). The samples were sequenced in 4 phases, referred to as processing cohorts. Amongst others, the age, sex, and ethnicity of each subject were collected. We assume that the replicates included in the study are technical replicates, though it is not specified in the original paper. Using Louvain clustering [18], the authors identified 23 clusters, which were assigned to 11 cell types. Additionally, we removed 2836 cells with more than 15% of mitochondrial DNA. The final scRNA-Seq count matrix has a total of 1260840 rows (cells) and 32738 columns (genes).

C.1.1 Control subset

To evaluate the methods and remove other possible confounding factors, such as age and sex, we have selected a homogeneous subset of 37 scRNA-Seq control samples from the two most frequent ethnicities in the dataset. 24 Caucasian and 5 Asian females between 24 and 28 years old (Supplementary Figure S14a). The samples collected in the UCSF Rheumatology Clinic were processed in the first 3 cohorts, except for the sample labelled *control* that was processed with the ImmVar samples in the fourth cohort; note that we have 25 subjects with only one single-cell sample and 4 subjects, all of them Caucasians, with at least 2 single-cell samples distributed over different Processing Cohorts. Three cell types (proliferating T and NK cells, progenitor cells, and plasmablasts) with less than 500 cells in total were removed from the scRNA-Seq dataset. Leaving 131862 cells from 8 cell types: CD4+ and CD8+ T cells (T4 and T8), CD14+ classical and CD16+ nonclassical monocytes (cM and ncM), conventional and plasmacytoid dendritic cells (cDC and pDC), natural killer cells (NK), and B cells (B). We pseudobulked the data into 296 pseudobulk samples. Genes with a count lower than 10 in five or more pseudobulk samples were also removed from the analysis. The final pseudobulk matrix has a total of 296 rows (pseudobulk samples) and 12034 columns (genes).

C.1.2 Case study subset

We selected a second subsample of 85 single-cell samples from 19 female SLE patients with no record of increased disease activity (as opposed to patients with registered flares) and 32 female healthy controls (Supplementary Figure S14b). In the group of healthy controls, there are 11 individuals with 2 replicates and

5 with 3 replicates. In the group of SLE patients, there is one patient with two replicates. The ages of the subjects vary between 30 and 34 years old. There is a confounding effect between the processing cohorts and the disease status. The cells from cell types not included in the simulation subset were removed from the analysis, leaving 14009 cells. The same criteria as in section C.1.1 were used to filter the genes. The final pseudobulk matrix has 680 rows (pseudobulk samples) and 12105 columns (genes).

C.2 Sets of negative control genes

We identified from the literature two sets of negative control genes: The first set has 118 cytosolic ribosome genes [2], and the second set has 1076 genes[8], included in the *scMerge* R package [9]. We compute the gene's standardised variances using the function FindVariableFeatures in the *Seurat* R package and select the genes with a variance lower than 1.8. The final sets of negative control genes have 105 and 102 genes, respectively. RUVIIIgb uses the cytosolic ribosome genes as negative control genes and RUVIIIscM uses the negative control genes from the *scMerge* R package. The negative control gene sets are visualised in the PCA plots from Figures F1 and F2 and clearly summarise the unwanted variation introduced by the processing cohorts.

D Baseline and gold standard models for the Pseudobulk RUV trails and the RUVIII-PBPS procedure

For the controls and case study datasets we first apply an upper-quartile normalisation and proceed to implement the RUV methods following the different trails proposed in Section **??**, for comparison purposes, we also define the following baseline model:

$$E(\log_2(\mathbf{Y}_t)|\mathbf{X}) = \mathbf{X}\boldsymbol{\beta}, \tag{19}$$

where $\log_2(\mathbf{Y}_t)$ is the $\log_2$ normalised count matrix from samples with a particular cell type t, $\mathbf{X}$ is the design matrix with the first column being a vector of ones and the second column containing the factor of interest (mock treatment or disease status); and $\boldsymbol{\beta}$, of dimension $2 \times G$ is the parameter matrix that includes the intercept and the parameter associated with the factor of interest.

In the original paper from the Lupus dataset[14], ethnicity was considered an important biological covariate, additionally, the exploratory analysis of the data identified a strong batch effect related to the processing cohorts. Therefore in our gold standard models, we also include the variables ethnicity and processing cohort. First, we only fit an intercept for each variable,

$$E(\log_2(\mathbf{Y}_t)|\mathbf{X}, \mathbf{Z}, \mathbf{U}) = \mathbf{X}\boldsymbol{\beta} + \mathbf{Z}\boldsymbol{\nu} + \mathbf{U}\boldsymbol{\alpha}, \tag{20}$$

where $\mathbf{Z}$ of dimension $S_t \times 1$ and $\mathbf{U}$ od dimension $S_t \times 3$ are the matrices that represent the ethnicity and processing cohort information with dummy variables, and $\boldsymbol{\nu}$ and $\boldsymbol{\alpha}$, are the parameters associated to the biological and batch covariates, respectively. Next, we include an interaction between the factor of interest and ethnicity,

$$E(\log_2(\mathbf{Y}_t)|\mathbf{X}, \mathbf{Z}, \mathbf{U}) = \mathbf{X}\boldsymbol{\beta} + \mathbf{Z}^{*}\boldsymbol{\nu}^{*} + \mathbf{U}\boldsymbol{\alpha}, \tag{21}$$

where $\mathbf{Z}^*$ is a matrix of dimension $S_t \times 2$ containing one dummy variable representing the ethnicity covariate and another dummy variable representing the interaction between a specific level of the factor of interest and a specific level of ethnicity. Similarly, ν^* is a $2 \times G$ matrix containing the parameters associated with ethnicity and the interaction term.

We will refer to the model in Equation (19) as the UQ model, because of the upper-quartile normalisation. We will refer to the models in Equations (20) and (21) as UQ Batch models, because they have information about the processing cohorts. Whether the interaction term between ethnicity and the factor of interest is used or not depends on the context and will be specified in the text.

Furthermore, the term “normalised counts matrix” will refer to both the matrix obtained through a naive approach, such as applying upper-quartile normalisation (as in the UQ model), and the matrix obtained by regressing out the unwanted factors: either known (as in the UQ Batch models), or unknown (as in the RUV models).

E Metrics

E.1 Evaluating the removal of unwanted variation in the counts matrix

The following methods were used to assess in a qualitative and quantitative manner how well each model removes the unwanted variation (processing cohort effects) while preserving the biological information (disease status) in the data.

E.1.1 PCA

For a given cell type t, a principal component analysis (PCA) is applied to the $\log_2$-transformed and normalised pseudobulk count matrices Y_t. The first two dimensions are plotted and coloured either according to the factors of interest or to the variables related to the unwanted factors (such as processing cohorts).

E.1.2 RLE plots

The Relative Log Expression (RLE) plots have the purpose of revealing sample heterogeneity [5]. In general, for a $N \times G$ log2-transformed gene expression matrix $\log_2(\mathbf{Y})$, the RLE plot is constructed as follows:

- Center the log expression of each column of $\log_2(\mathbf{Y})$ by subtracting the gene-specific median $\log_2(\tilde{y_g})$
- For each row n, generate a boxplot of all the deviations for that sample.

When applying the RLE plots to a pseudobulk count matrix $\mathbf{Y}_p$, for S samples and G genes, the genes' log expression will change from one cell type to another, therefore the plots are created for each cell type separately.

Assuming the log expression of most genes is not affected by biological variables, the boxplots are expected to show only random variability. Therefore, RLE plots from homogeneous boxplots will indicate the successful removal of unwanted variation.

E.1.3 Average Silhoutte Width (ASW)

A silhouette width quantifies how well a data point lies within its cluster [17]. It requires a distance matrix and cluster labels. Values close to one indicate a good match of a data point to its current cluster, and lower values indicate a poor match. The average silhouette width (ASW) is often used to compare clustering results.

We compute the Euclidean distances between samples using the first two principal components and use one of the biological or known sources of unwanted variation as clustering labels for calculating the silhouette widths of each sample. Subsequently, the values are averaged to obtain the ASW.

Since we observed that the variation associated with covariates can be captured with higher order principal components, we also compute the ASW using the first ten principal components. When evaluating the first 10 principal components, we rely on the Manhattan distance as it was previously observed to perform better in high dimensional matrices [1].

Higher ASW values indicate that the covariate is associated with the variation in the data, whereas low values indicate low or no signal in the data. A good RUV method keeps or increases the ASW of biological covariates and decreases the ASW of known sources of unwanted variation.

E.2 Evaluating the impact of unwanted factors in differential expression analysis

E.2.1 In silico Differential Gene Expression

We randomly assigned a mock treatment to the subjects in the simulation subset introduced in Section C.1. The mock treatment has two possible values, "A" and "B". To simulate differential gene expression (DGE), we randomly select 10% of the genes, and, in pairs, swap the gene expression of all the samples from the mock treatment "A" for each pair of genes. This simulation strategy has been previously proposed [11], and is implemented in the *swapper* R package [10]. This procedure creates a differential gene expression signal of different magnitudes between the treatment groups and attempts to retain the correlation structure of the original data as much as possible, as well as the unwanted variation that we intend to remove.

We switch a different set of genes per cell type and assign the mock treatment with equal and unequal probabilities to subjects from different processing cohorts. When the mock treatment is assigned with equal probabilities, we refer to it as a balanced design, and in the other case, we refer to it as an unbalanced design. Note that the unbalanced design generates a confounding effect between the processing cohort and the mock treatment. Specifically, we introduce an artificial confounding effect between the processing cohorts and the mock treatment by increasing the probability of receiving the mock treatment "A" to 90% in the processing cohort 4, while maintaining the probability at 50% for the other three processing cohorts. For both designs, we generate 100 simulated datasets. By labelling the swapped genes as differentially expressed, we can compute the true positive and false discovery rates.

E.2.2 True Positive Rate and False Discovery Rate

For each simulated dataset, we assess DE for each cell type separately using limma-voom [6]. A detailed description of the pipeline can be found in Appendix G. Later we gather the 100 sets of G p-values and compute the FDR and the TPR at a nominal FDR of $1\%, 5\%$ and 10%. A good combination of trail and model will result in a high TPR with an empirical FDR close to, or lower than, the nominal values.

F Consequences of misspecified negative control genes

To illustrate the consequences of misspecifying the set of negative control genes, we have performed a different simulation. Here, we first simulate the differential expression and later select the genes with a low standardised variance as negative controls. Note that some of the genes selected as negative control genes are differentially expressed. We replicate the simulation 100 times to compute the FDR and the TPR.

In Figure F3 We observe that in the pseudobulk samples from CD4+ T cells, misspecifying the set of negative control genes results in reduced detection power in the RUV2 model (RUV2) and an elevated false discovery rate (FDR) in the RUVIII model (RUVIII). Alternatively, we can opt not to specify any negative control genes in the RUVIII method and rely exclusively on the negative control samples (RUVIII noncg). This approach demonstrates superior performance, maintaining a high true positive rate (TPR) while reducing the FDR. However, the results might be improved by including more technical replicates, particularly between the processing cohort 4 and other processing cohorts.

G DEA pipeline for pseudo bulk studies

We propose the following process using the limma-voom method to perform differential expression analysis in pseudobulk counts.

1. Preprocessing: Filter low-quality cells and genes with low counts.

2. Aggregation: Create the pseudo bulk matrix from a single cell dataset.

3. Transform: We compute the normalisation factors using an Upper Quantile normalisation and return the normalised log2-transformed count per million matrix (logcpm).

4. Remove unwanted variation: We use an RUV method (RUV2, RUV4, or RUVIII) to compute the W factors.

5. Design: Create the design matrix with the factors of interest and the $\mathbf{W}$ factors.

6. Estimate weights: We use the pseudobulk count matrix and the normalisation factors as input to estimate the weights from the voom method [6], the output is a logcpm transformed matrix and the precision weights.

7. Estimate: We estimate the model parameters $\boldsymbol{\alpha}$ and $\boldsymbol{\beta}$ in Equation **??** using the empirical Bayes pipeline from limma with the precision weights [16].

8. Test: We compute the moderated t-statistic and test for differential expression.

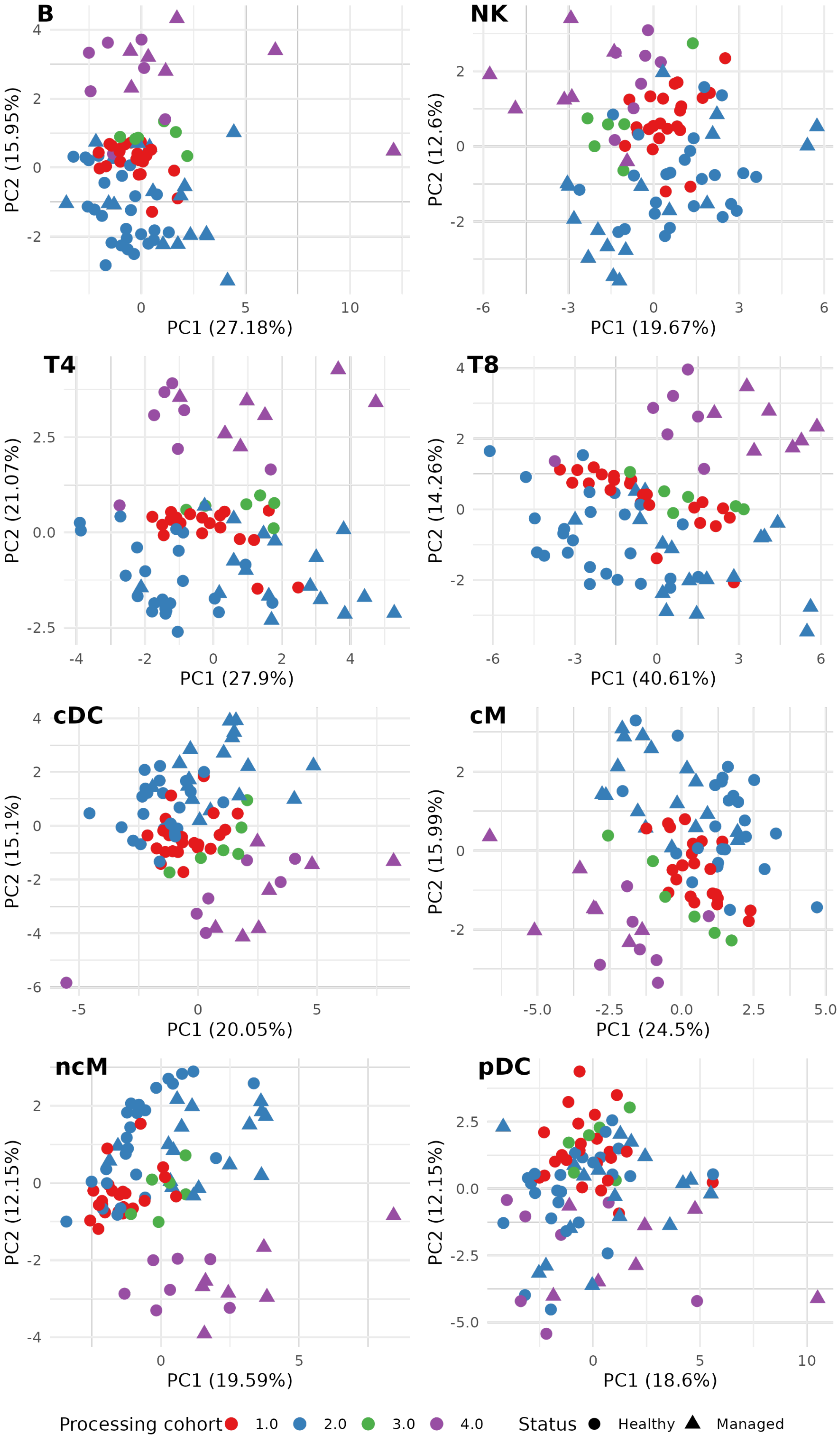

Figure F1: PCA plots after UQ normalisation of the pseudobulk samples from the case study subset and the genes from the cytosolic ribosome set with a standardised variance lower than 1.8, organised by cell type.

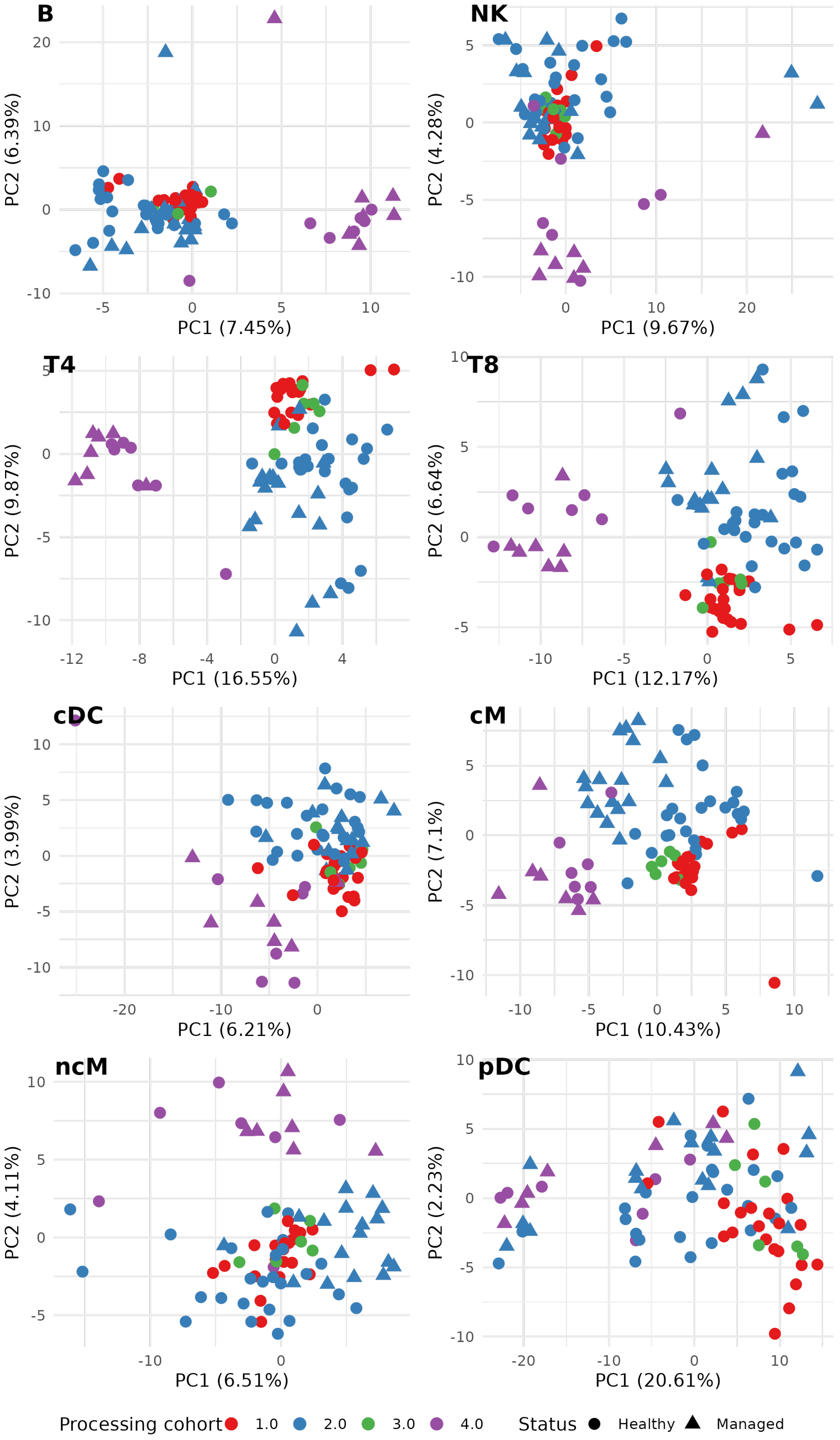

Figure F2: PCA plots after UQ normalisation of the pseudobulk samples from the case study subset and the genes included in the scMerge R package with a standardised variance lower than 1.8, organised by cell type.

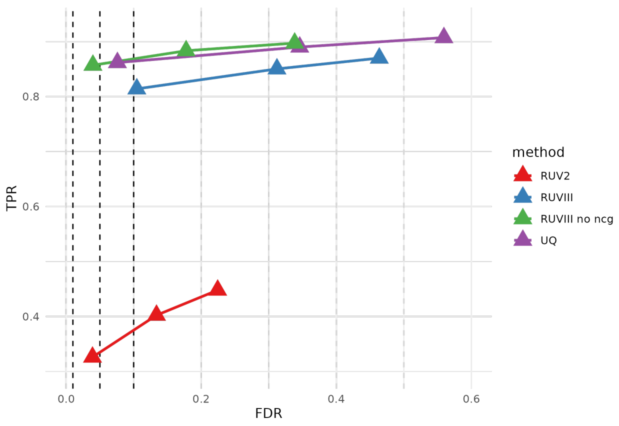

Figure F3: FDR vs TPR when the negative control genes (ncg) are misspecified (as in RUV2 and RUVIII) or not used (as in RUVIII no ncg and UQ).

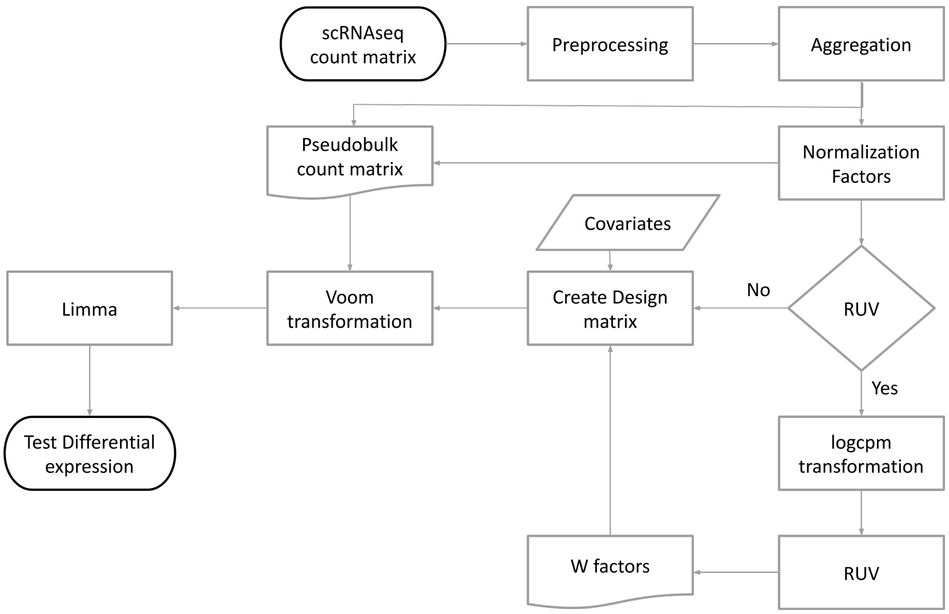

Figure F4: DEA pipeline

References

[1] Charu C Aggarwal, Alexander Hinneburg, and Daniel A Keim. "On the surprising behavior of distance metrics in high dimensional space". In: *Database theory—ICDT 2001: 8th international conference London, UK, January 4–6, 2001 proceedings 8*. Springer. 2001, pp. 420–434.

[2] Julie M Deeke and Johann A Gagnon-Bartsch. "Stably expressed genes in single-cell RNA sequencing". In: *Journal of Bioinformatics and Computational Biology* 18.01 (2020), p. 2040004.

[3] Johann A Gagnon-Bartsch, Laurent Jacob, and Terence P Speed. "Removing unwanted variation from high dimensional data with negative controls". In: *Berkeley: Tech Reports from Dep Stat Univ California* (2013), pp. 1–112.

[4] Johann A Gagnon-Bartsch and Terence P Speed. "Using control genes to correct for unwanted variation in microarray data". In: *Biostatistics* 13.3 (2012), pp. 539–552.

[5] Luke C Gandolfo and Terence P Speed. "RLE plots: Visualizing unwanted variation in high dimensional data". In: *PloS one* 13.2 (2018), e0191629.

[6] Charity W Law et al. "voom: Precision weights unlock linear model analysis tools for RNA-seq read counts". In: *Genome biology* 15 (2014), pp. 1–17.

[7] Jeffrey T Leek and John D Storey. "Capturing heterogeneity in gene expression studies by surrogate variable analysis". In: *PLoS genetics* 3.9 (2007), e161.

[8] Yingxin Lin et al. "Evaluating stably expressed genes in single cells". In: *GigaScience* 8.9 (2019), giz106.

[9] Yingxin Lin et al. "scMerge leverages factor analysis, stable expression, and pseudoreplication to merge multiple single-cell RNA-seq datasets". In: *Proceedings of the National Academy of Sciences* 116.20 (2019), pp. 9775–9784.

[10] Milan Malfait. *Simulate DE Signal By Feature Swapping — milanmlft.github.io*. `milanmlft.github.io/swapper/`. [Accessed 30-07-2024]. 2022.

[11] Milan Malfait et al. "Strategies for addressing pseudoreplication in multi-patient scRNA-seq data". In: *bioRxiv* (2024), pp. 2024–06.

[12] Ramyar Molania et al. "A new normalization for Nanostring nCounter gene expression data". In: *Nucleic Acids Research* 47.12 (2019), pp. 6073–6083.

[13] Ramyar Molania et al. "Removing unwanted variation from large-scale RNA sequencing data with PRPS". In: *Nature Biotechnology* 41.1 (2023), pp. 82–95.

[14] Richard K Perez et al. "Single-cell RNA-seq reveals cell type–specific molecular and genetic associations to lupus". In: *Science* 376.6589 (2022), eabf1970.

[15] CZI Cell Science Program et al. "CZ CELLxGENE Discover: a single-cell data platform for scalable exploration, analysis and modeling of aggregated data". In: *Nucleic Acids Research* 53.D1 (Nov. 2024), pp. D886–D900. ISSN: 1362-4962. DOI: `10.1093/nar/gkae1142`. eprint: `https://academic.oup.com/nar/article-pdf/53/D1/D886/60882786/gkae1142.pdf`. URL: `https://doi.org/10.1093/nar/gkae1142`.

[16] Matthew E Ritchie et al. "limma powers differential expression analyses for RNA-sequencing and microarray studies". In: *Nucleic acids research* 43.7 (2015), e47–e47.

[17] Peter J Rousseeuw. "Silhouettes: a graphical aid to the interpretation and validation of cluster analysis". In: *Journal of computational and applied mathematics* 20 (1987), pp. 53–65.

[18] Vincent A Traag, Ludo Waltman, and Nees Jan Van Eck. "From Louvain to Leiden: guaranteeing well-connected communities". In: *Scientific reports* 9.1 (2019), pp. 1–12.

H Supplementary tables

	W1	W2	W3
B	8.9E-12	3.5E-09	6.5E-05
NK	7.3E-08	5.9E-09	2.4E-03
T4	2.3E-12	7.6E-05	1.6E-02
T8	1.0E-10	7.3E-10	2.5E-04
cDC	3.8E-08	3.8E-10	2.8E-08
cM	1.9E-12	5.0E-07	4.1E-10
ncM	1.9E-08	1.9E-08	2.3E-07
pDC	6.2E-08	8.7E-04	4.1E-04

Table S1: Kruskal-Wallis p-values when testing the association between the unwanted factors of RUVIIIscM in the case study and the processing cohort

	W1	W2	W3
B	5.9E-05	1.6E-09	1.2E-02
NK	8.9E-04	7.0E-08	2.1E-03
T4	5.5E-12	5.6E-05	7.3E-01
T8	3.0E-08	6.6E-07	1.0E-01
cDC	1.6E-05	2.6E-08	3.5E-04
cM	1.1E-11	5.4E-06	2.7E-01
ncM	8.0E-08	3.1E-09	2.2E-06
pDC	1.8E-07	2.3E-07	2.3E-03

Table S2: Kruskal-Wallis p-values when testing the association between the unwanted factors of RUVIIIgb in the case study and the processing cohort

	UQ	Batch	RUVIIIgb	RUVIIIscM
RBCK1	5	0	0	0
TMEM140	0	0	0	0
AIF1	2	0	1	0
IFNGR1	0	0	1	1
IFNGR2	0	0	0	0
CCL3	2	1	0	0
CCL5	2	0	0	0
CCL4	1	0	1	1
FITM1	0	0	0	0
SOCS1	2	0	1	1
GADD45B	0	0	0	0
UNC93B1	1	0	1	0
IFNGR1.1	0	0	1	1
IRF8	2	0	0	0
PARP10	1	0	1	1
IFNAR2	0	0	0	0
IFNAR1	0	0	0	0
IP6K2	2	0	0	0
REC8	2	0	0	0

Table S3: ISGs not detected by at least one model (UQ, Batch, RUVIIIgb, RUVIIIscM) in any of the 8 cell types analysed in the lupus case study, with the number of cell types that detected the gene as differentially expressed for each model

	W1	W2	W3
B	3.6E-07	7.2E-13	1.6E-10
NK	7.4E-06	3.0E-12	7.0E-10
T4	6.5E-08	1.4E-12	4.6E-10
T8	1.8E-07	3.8E-13	8.1E-07
cDC	4.0E-07	2.6E-11	6.5E-10
cM	3.4E-07	6.2E-13	9.1E-07
ncM	1.4E-04	8.9E-13	1.9E-06
pDC	1.2E-08	1.5E-05	5.4E-01

Table S4: Kruskal-Wallis p-values when testing the association between the unwanted factors of RUVIII PBPS in the case study and the processing cohort

	UQ	Batch	RUVIIIpbps
PML	5	4	0
RBCK1	5	0	0
TMEM140	0	0	0
AIF1	2	0	0
IFNGR1	0	0	0
IFNGR2	0	0	0
CCL5	2	0	0
CCL4	1	0	0
FITM1	0	0	0
SOCS1	2	0	0
GADD45B	0	0	0
UNC93B1	1	0	0
IFNGR1.1	0	0	0
IRF8	2	0	0
PARP10	1	0	1
IFNAR2	0	0	0
IFNAR1	0	0	0
IP6K2	2	0	0
REC8	2	0	0

Table S5: ISGs not detected by at least one model (UQ, Batch, RUVIIIpbps) in any of the 8 cell types analysed in the lupus case study, with the number of cell types that detected the gene as differentially expressed for each model

I Supplementary figures

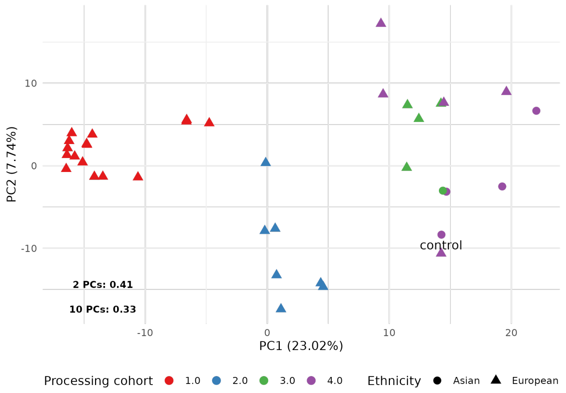

(a)

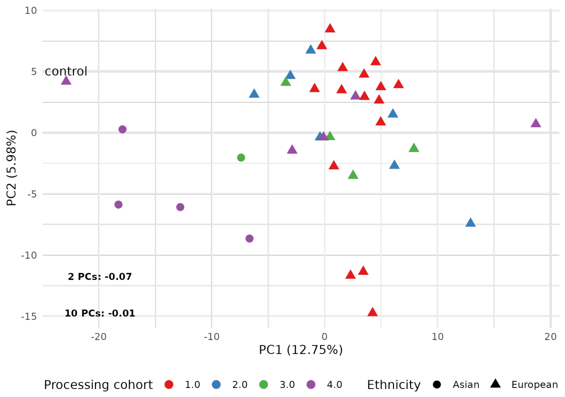

(b)

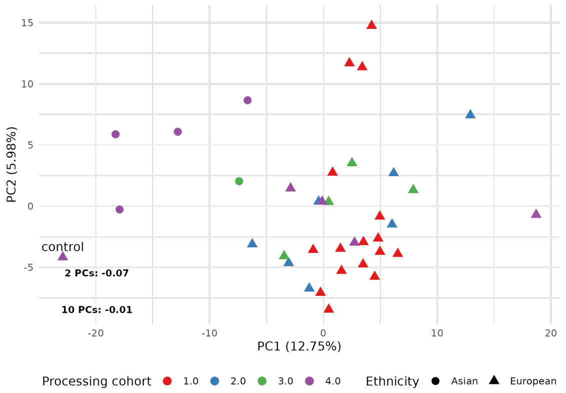

(c)

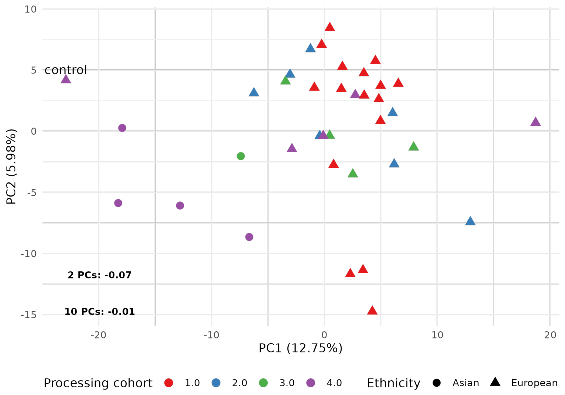

(d)

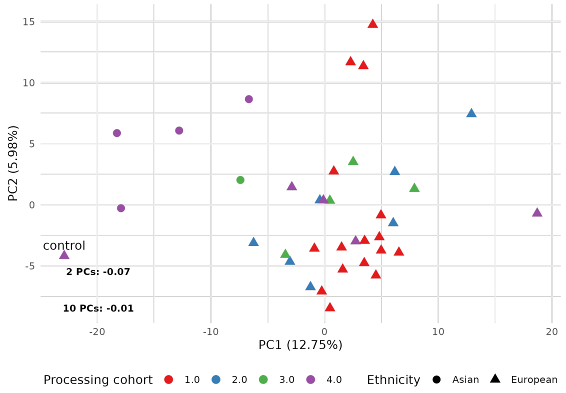

(e)

Figure S1: PCA plots and ASW of pseudobulk samples from CD4+ T cells in the control subset. **(a)** After UQ normalisation. **(b)** After UQ and RUV2 normalisation following Trail 1. **(c)** After UQ and RUV2 normalisation following Trail 2. **(d)** After UQ and RUV2 normalisation following Trail 3. **(e)** After UQ normalisation and processing cohort effect substraction.

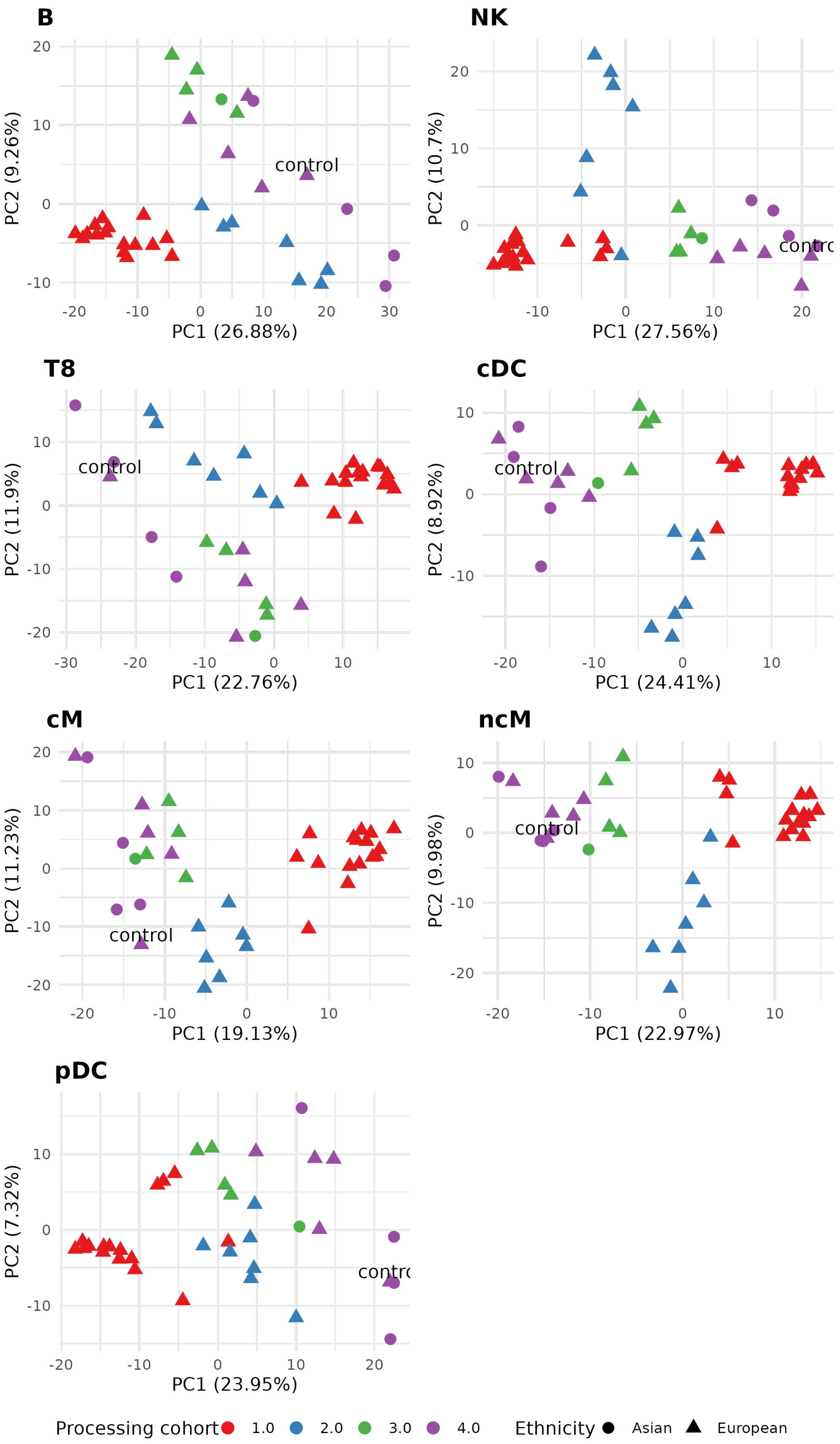

Figure S2: PCA plots of pseudobulk samples in the control subset after UQ normalisation, organised by cell type and processing cohort

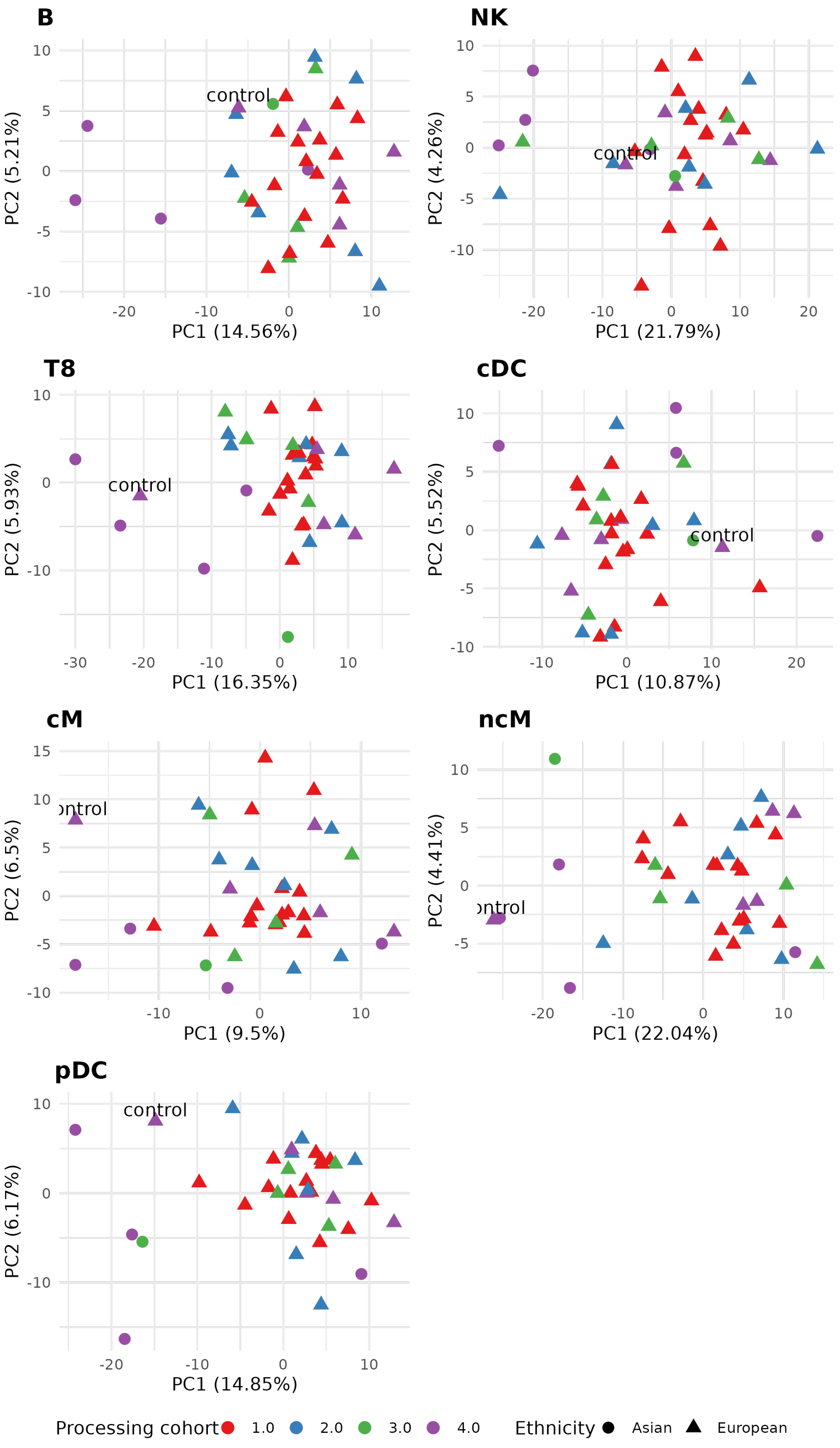

Figure S3: PCA plots of pseudobulk samples in the control subset after UQ normalisation and processing cohort effect substraction, organised by cell type and processing cohort

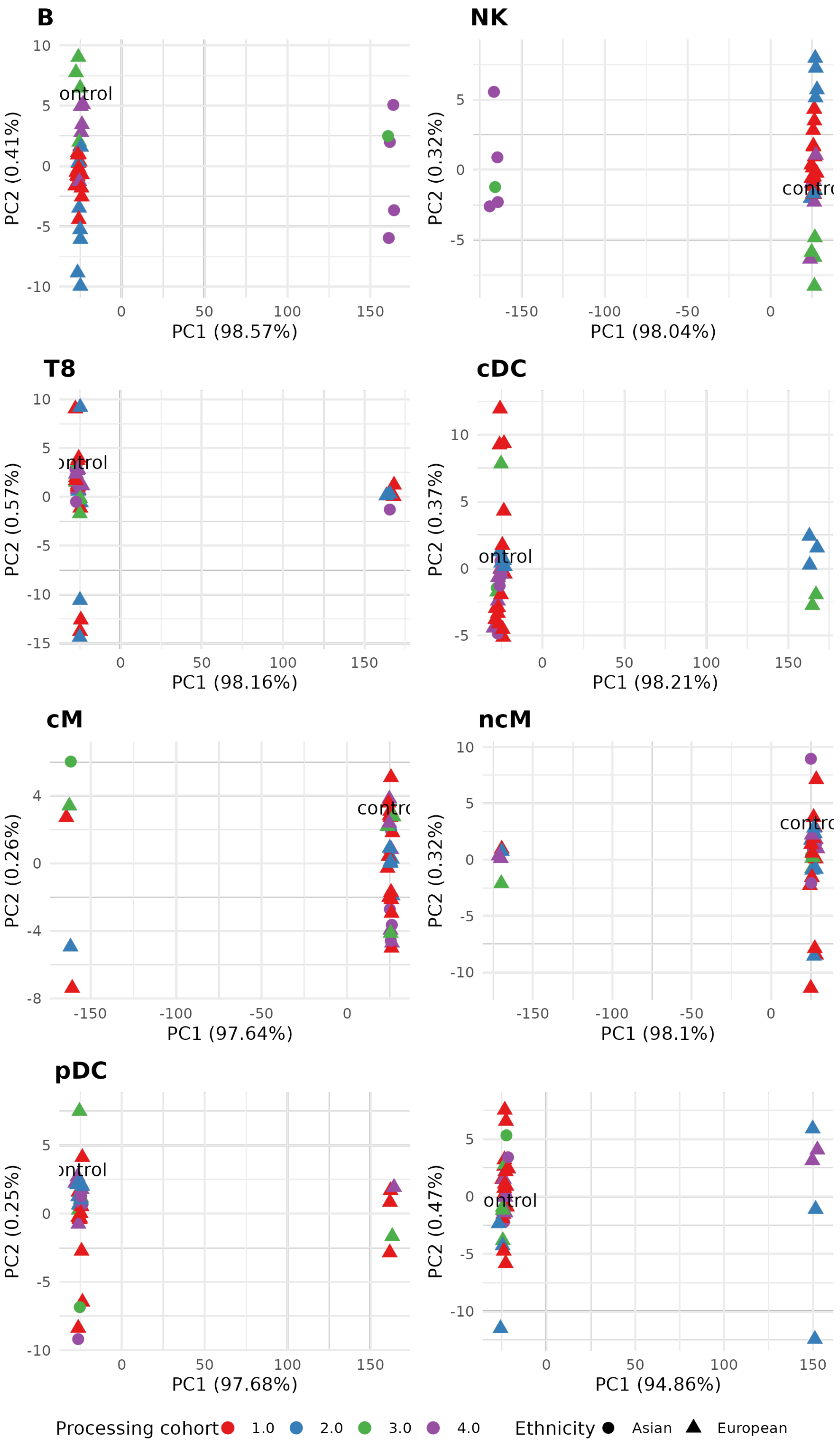

Figure S4: PCA plots of pseudobulk samples in the control subset after UQ and RUV2 normalisation following Trail 1, organised by cell type and processing cohort

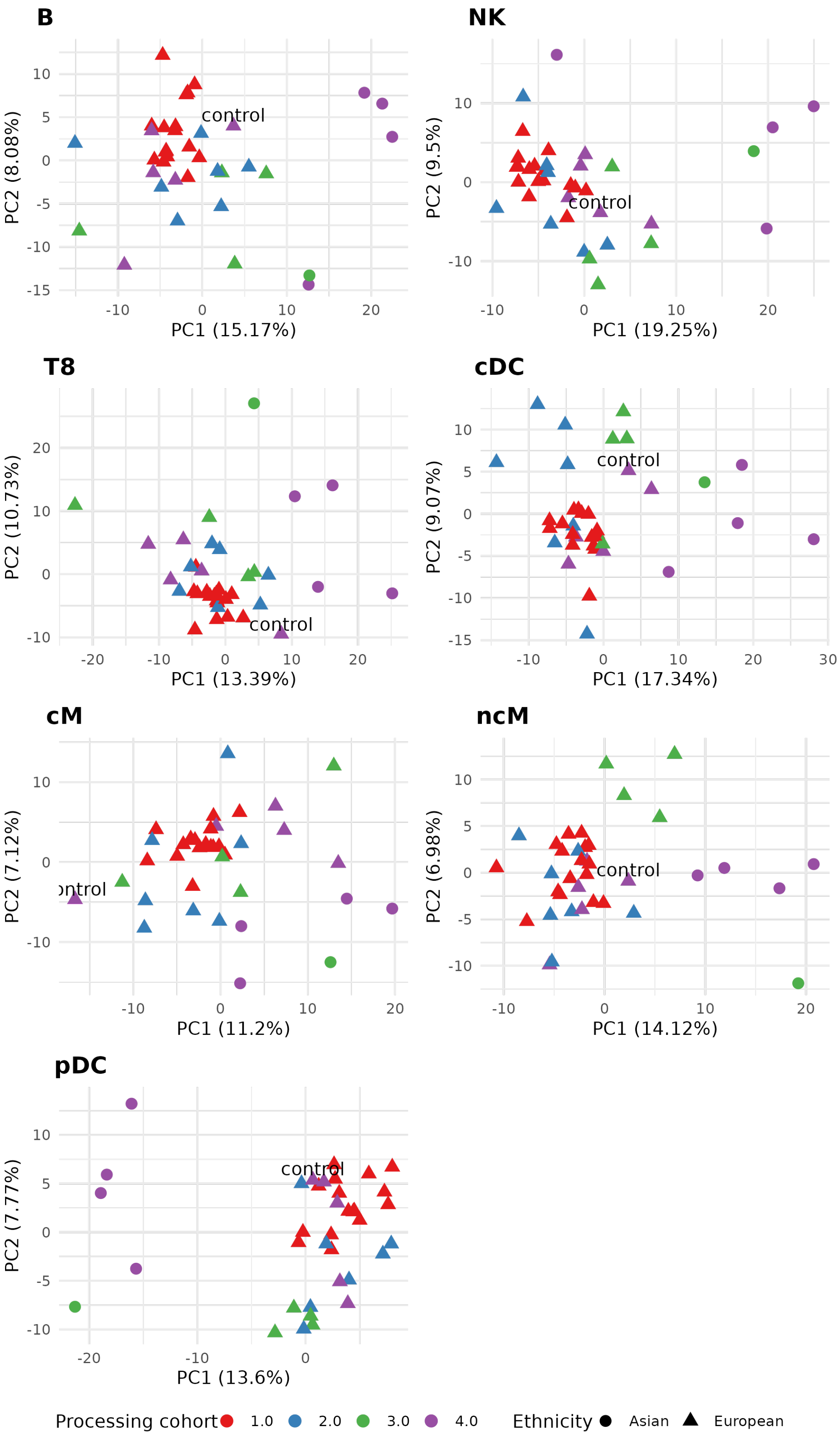

Figure S5: PCA plots of pseudobulk samples in the control subset after UQ and RUV2 normalisation following Trail 2, organised by cell type and processing cohort

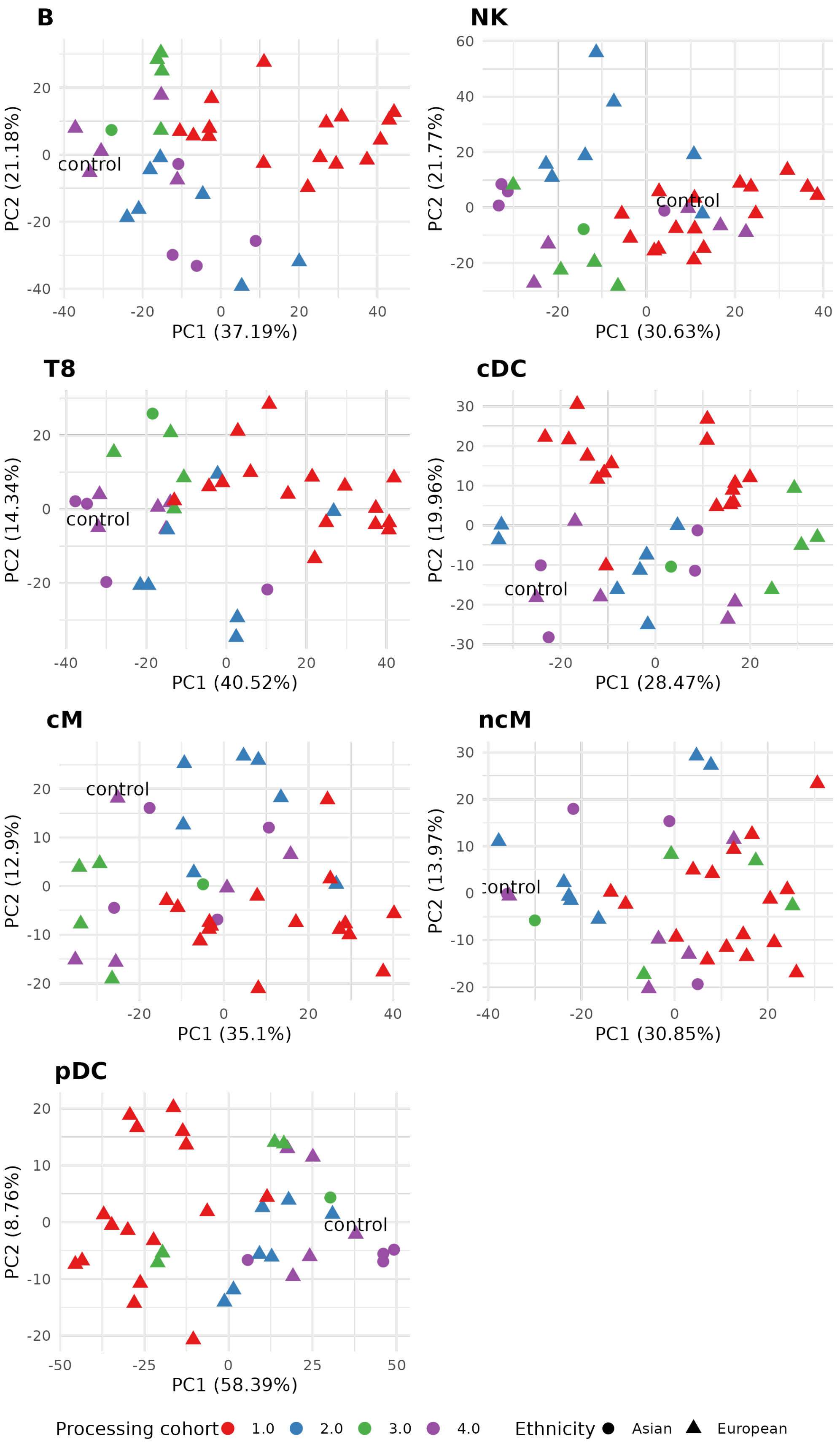

Figure S6: PCA plots of pseudobulk samples in the control subset after UQ and RUV2 normalisation following Trail 3, organised by cell type and processing cohort

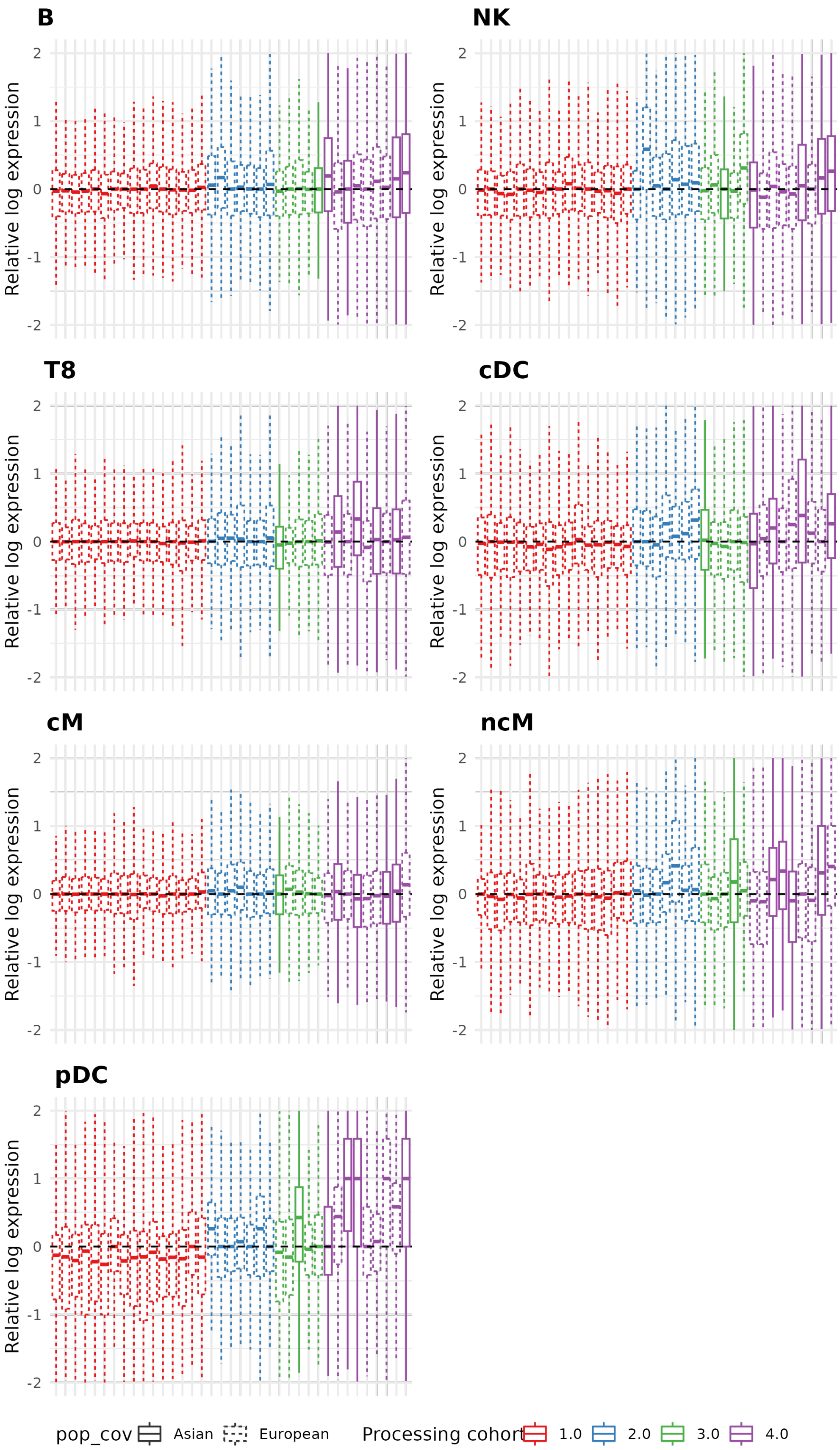

Figure S7: RLE plots of pseudobulk samples in the control subset organised by cell type and processing cohort

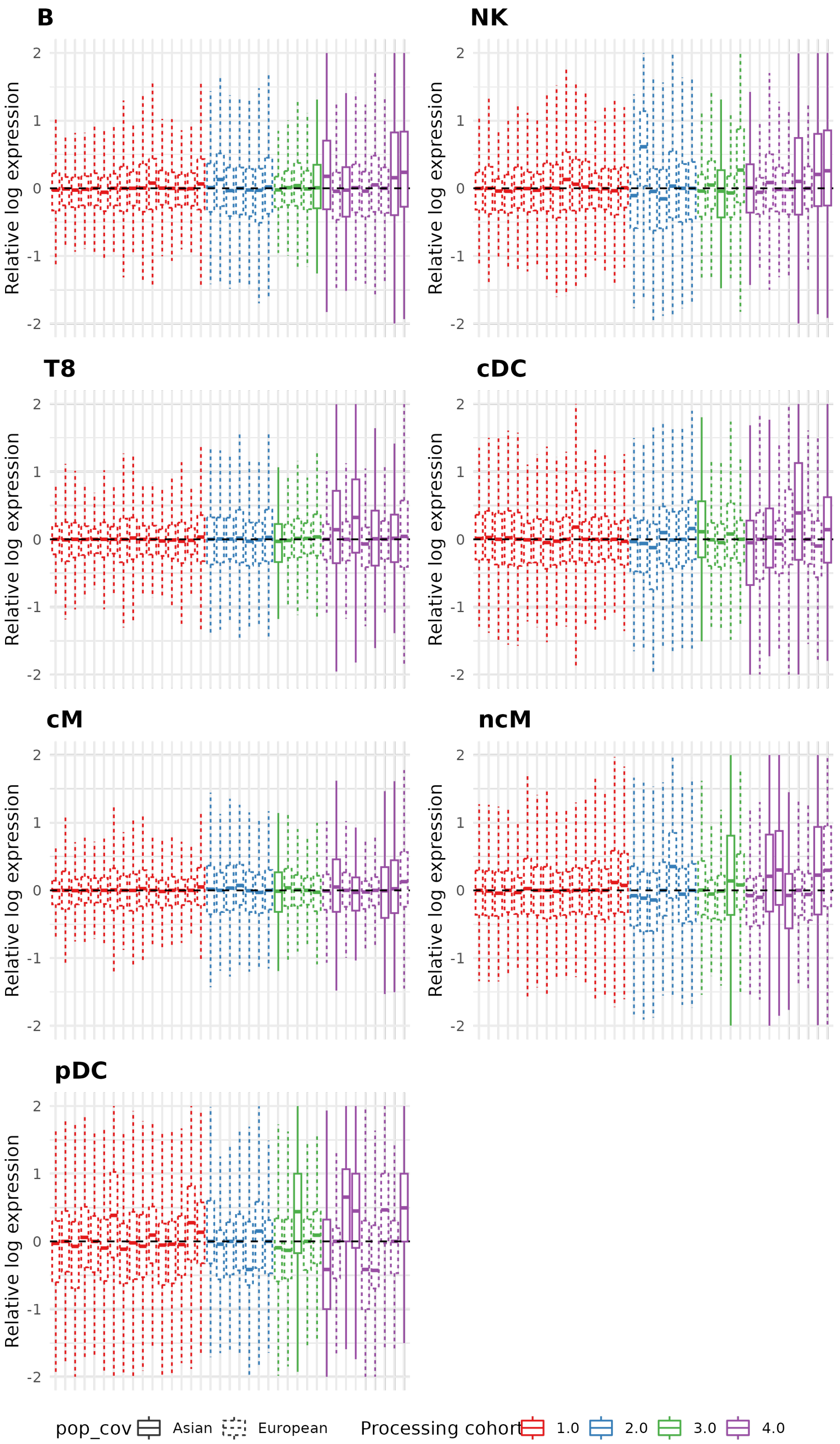

Figure S8: RLE plots of pseudobulk samples in the control subset after UQ normalisation and processing cohort effect substraction, organised by cell type and processing cohort

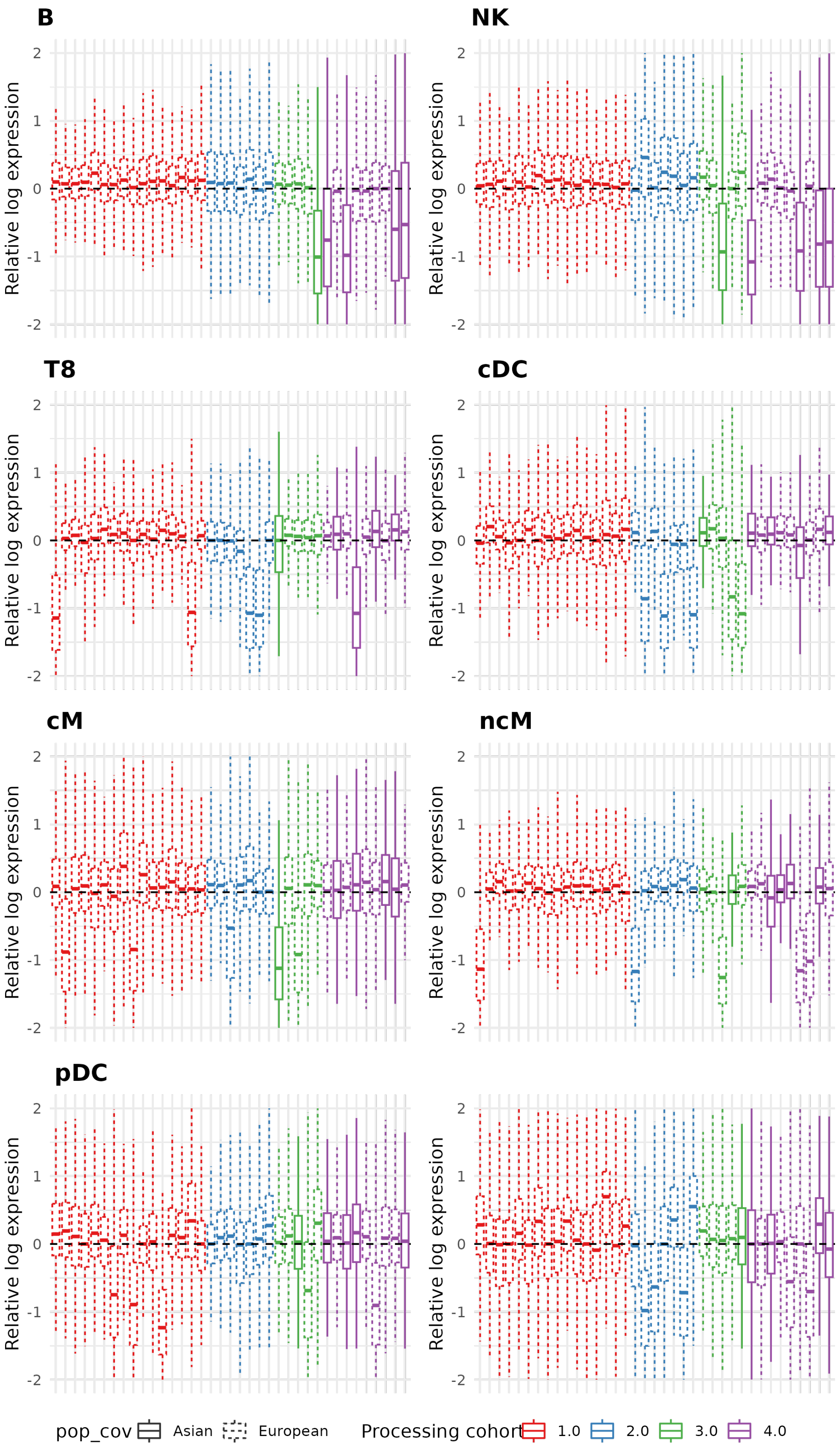

Figure S9: RLE plots of pseudobulk samples in the control subset after UQ and RUV2 normalisation following Trail 1, organised by cell type and processing cohort

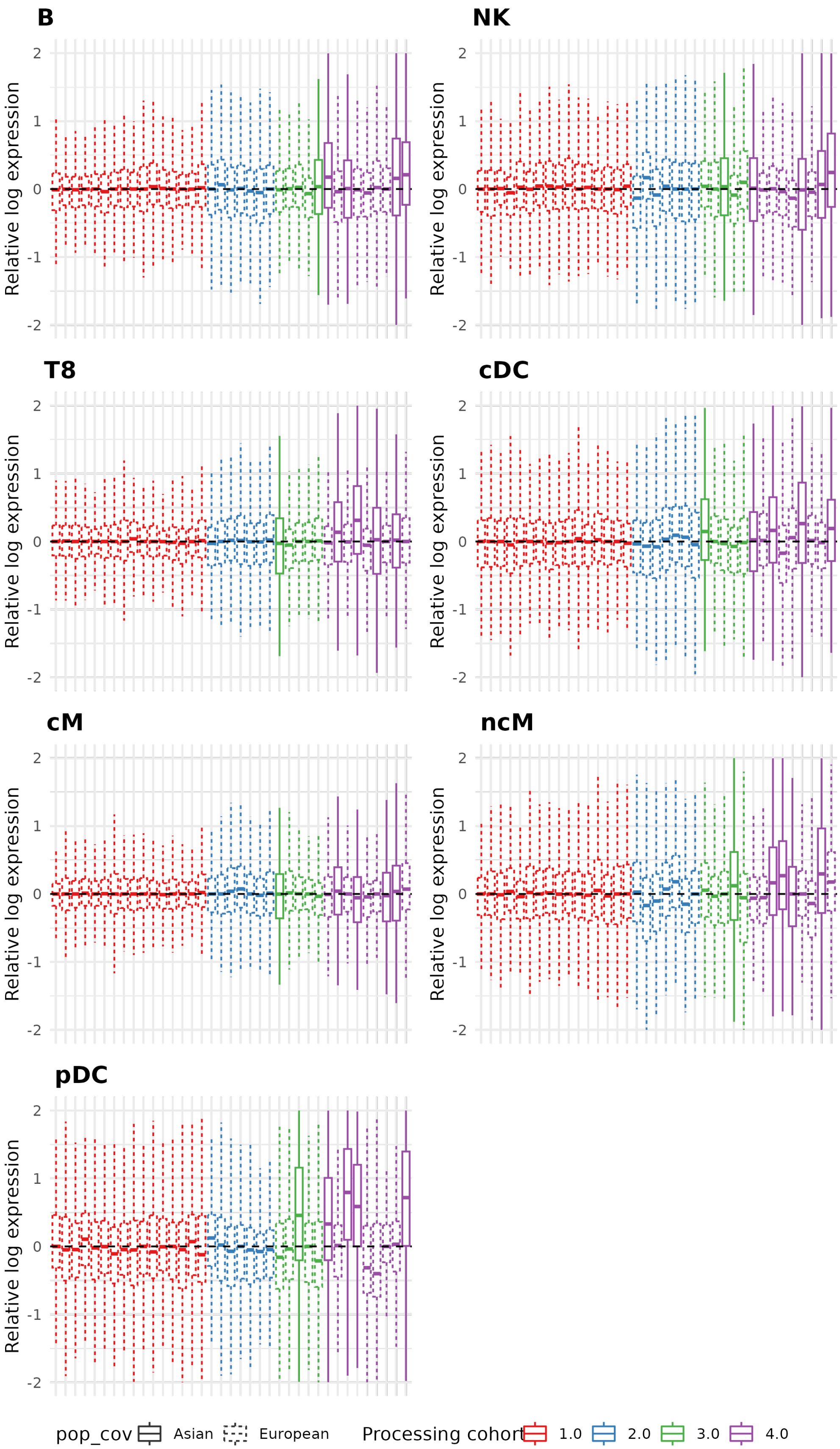

Figure S10: RLE plots of pseudobulk samples in the control subset after UQ and RUV2 normalisation following Trail 2, organised by cell type and processing cohort

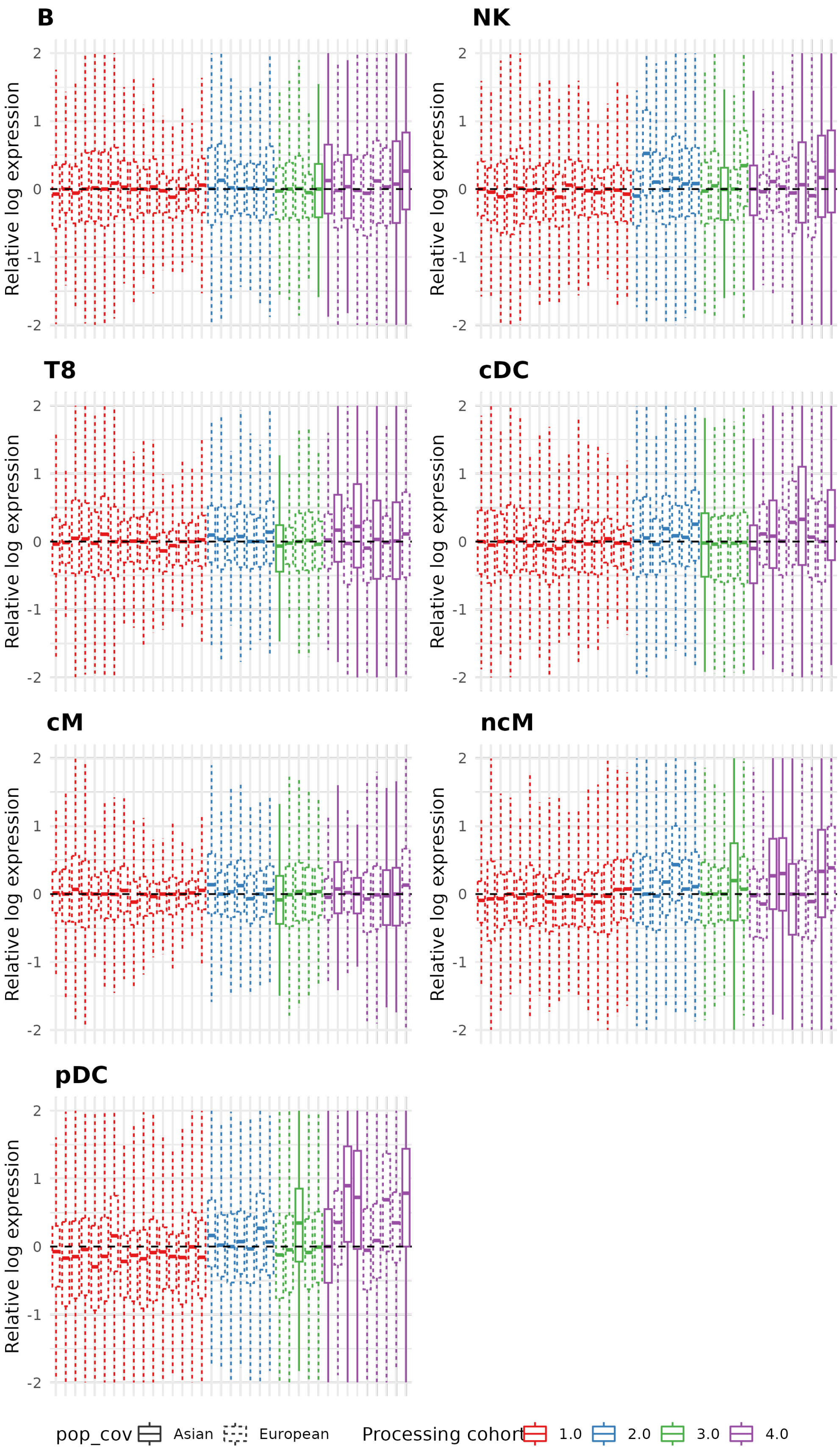

Figure S11: RLE plots of pseudobulk samples in the control subset after UQ and RUV2 normalisation following Trail 3, organised by cell type and processing cohort

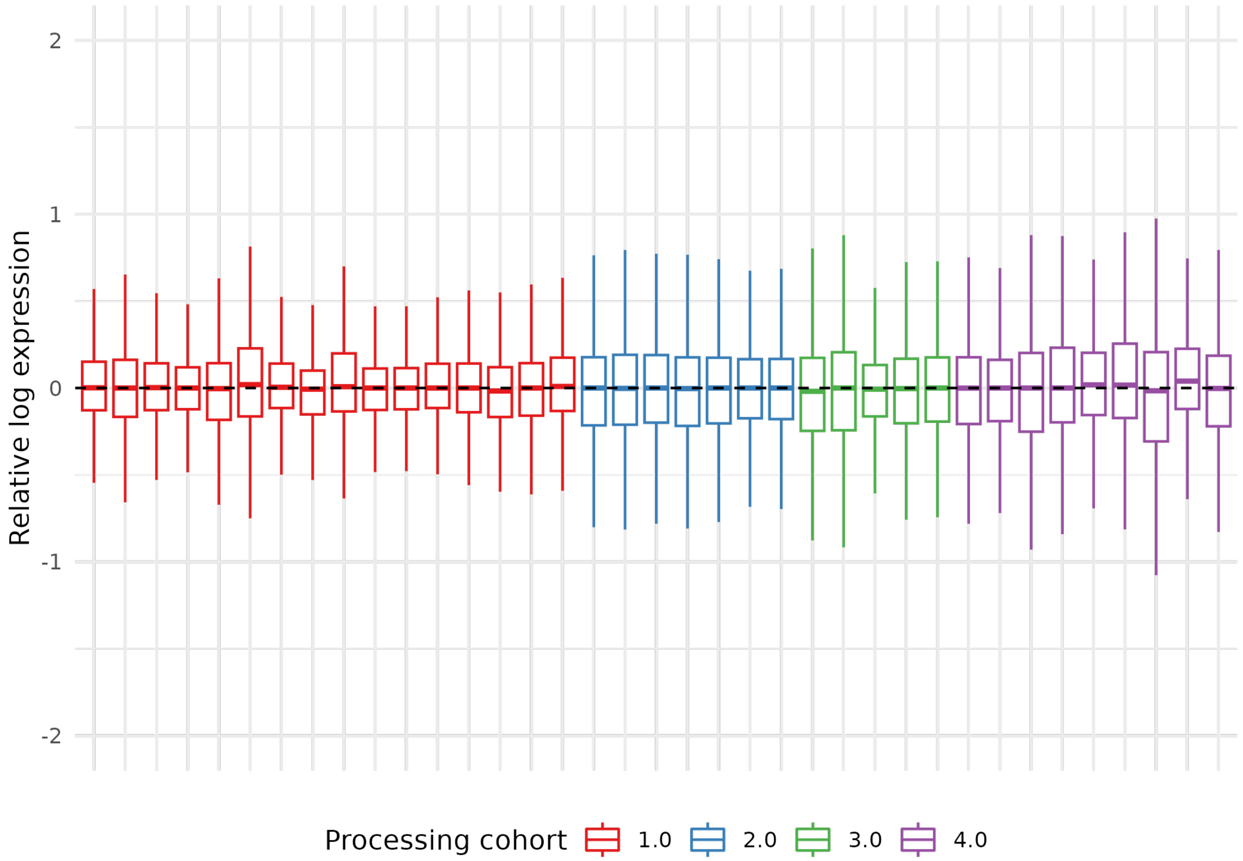

Figure S12: RLE plot of samples from the control subset aggregated by single-cell sample. Boxplots organised by processing cohort.

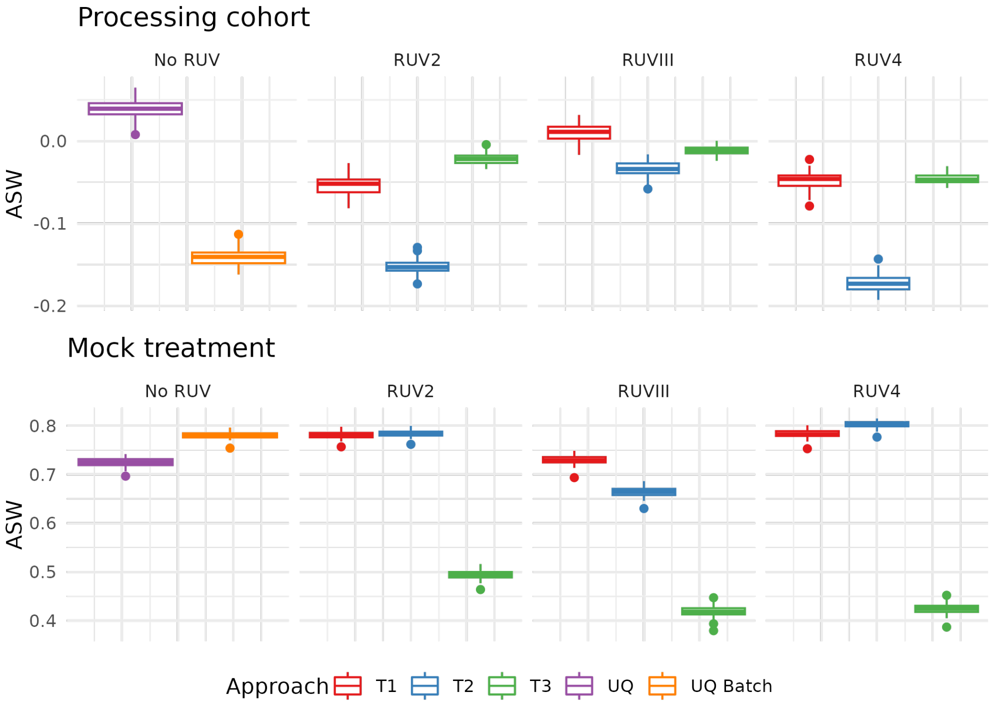

Figure S13: Boxplot of ASW scores computed on the first 10 PCs of 100 datasets with simulated differential expression across CD4+ T cells pseudobulk samples from the control subset. The processing cohort and the mock treatment were used as clustering labels.

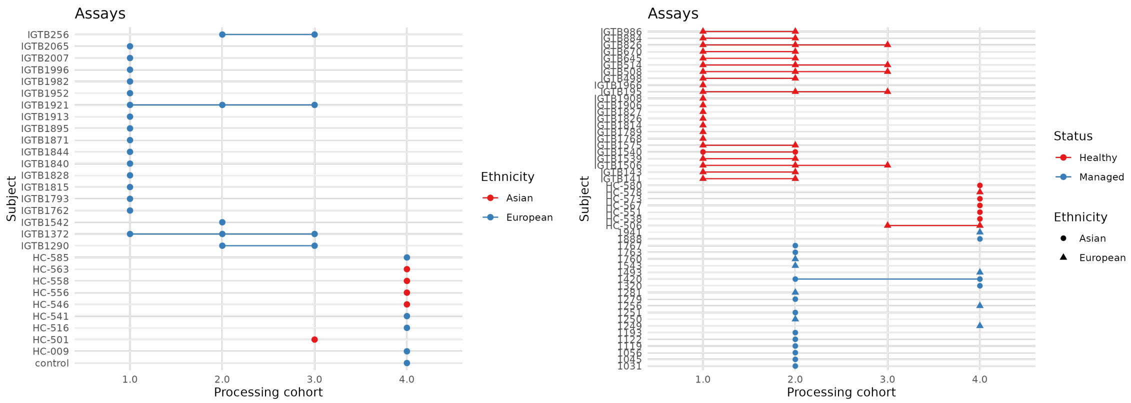

(a) (b)

Figure S14: Subsets drawn from the Lupus dataset. **(a)** Set of control samples organised by subject, processing cohort, and ethnicity. Used in the simulation of differential expression. **(b)** Set of controls and patients, organised by subject, processing cohort and disease status. Used in the case study

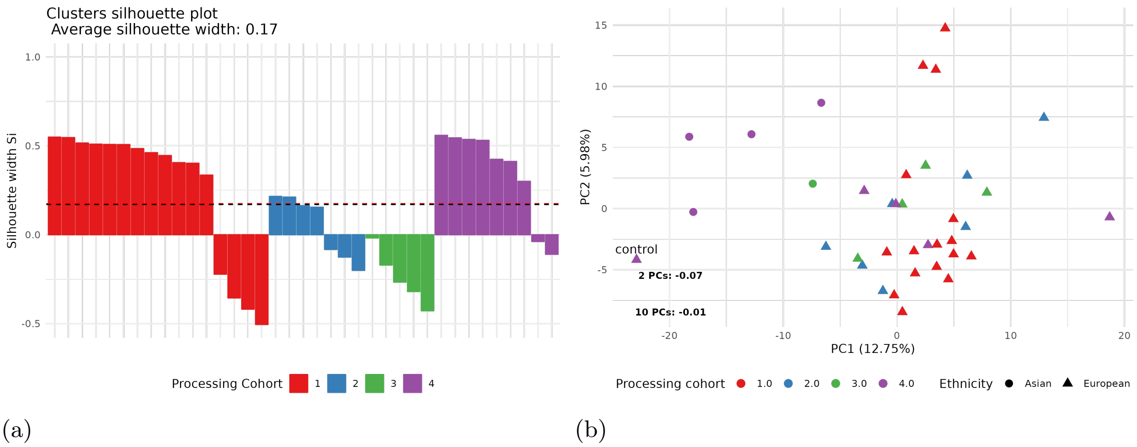

Figure S15: Processing cohort batch effects from CD4+ T cells samples after RUVIII normalisation. **(a)** Silhouette scores using two PCs and labelling by processing cohort. **(b)** PCA plot and ASW scores using 2 and 10 principal components.

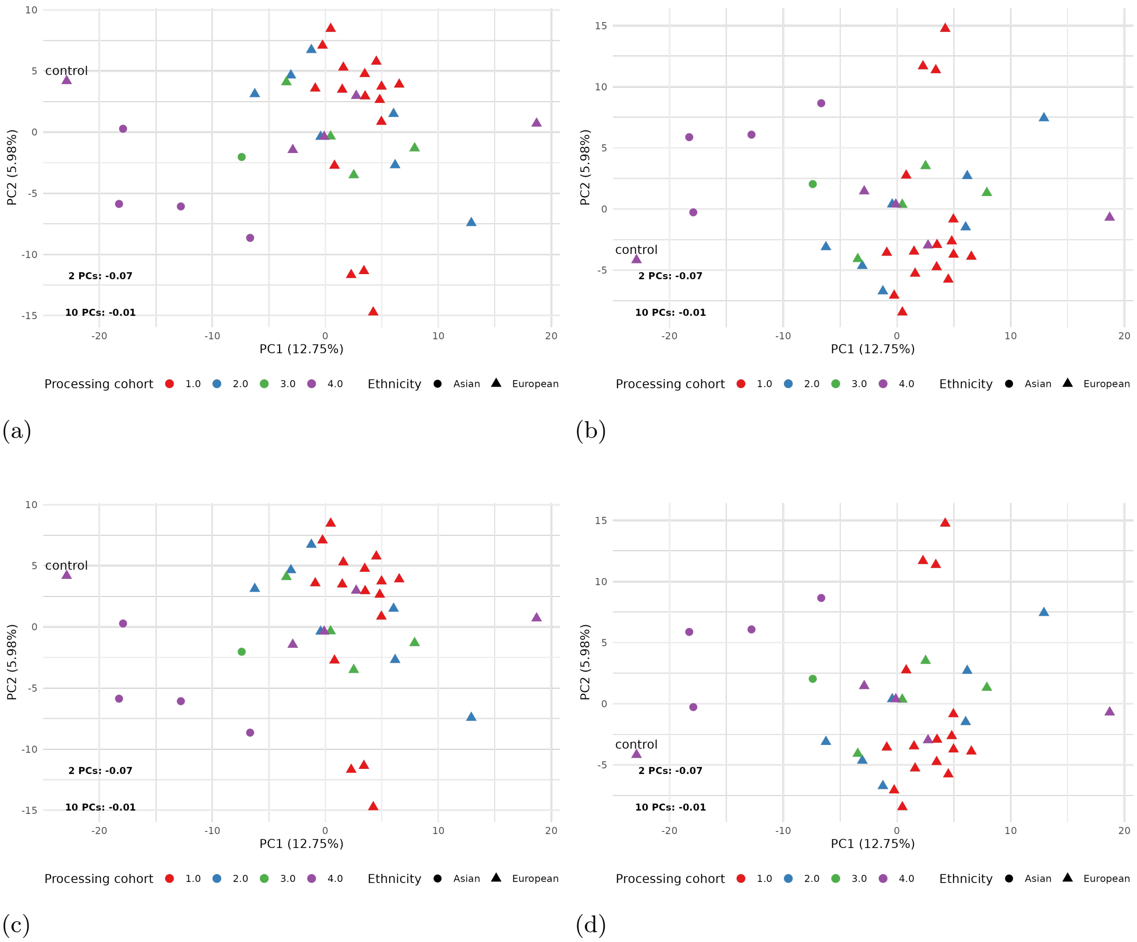

Figure S16: PCA plots and ASW of pseudobulk samples from CD4+ T cells in the control subset. **(a)** After UQ and RUVIII PBPS normalisation following Trail 2. **(b)** After UQ and RUVIII normalisation following Trail 2. **(c)** After UQ normalisation, and RUVIII normalisation following Trail 2 and processing cohort effect substraction.**(d)** After UQ normalisation and processing cohort effect substraction.

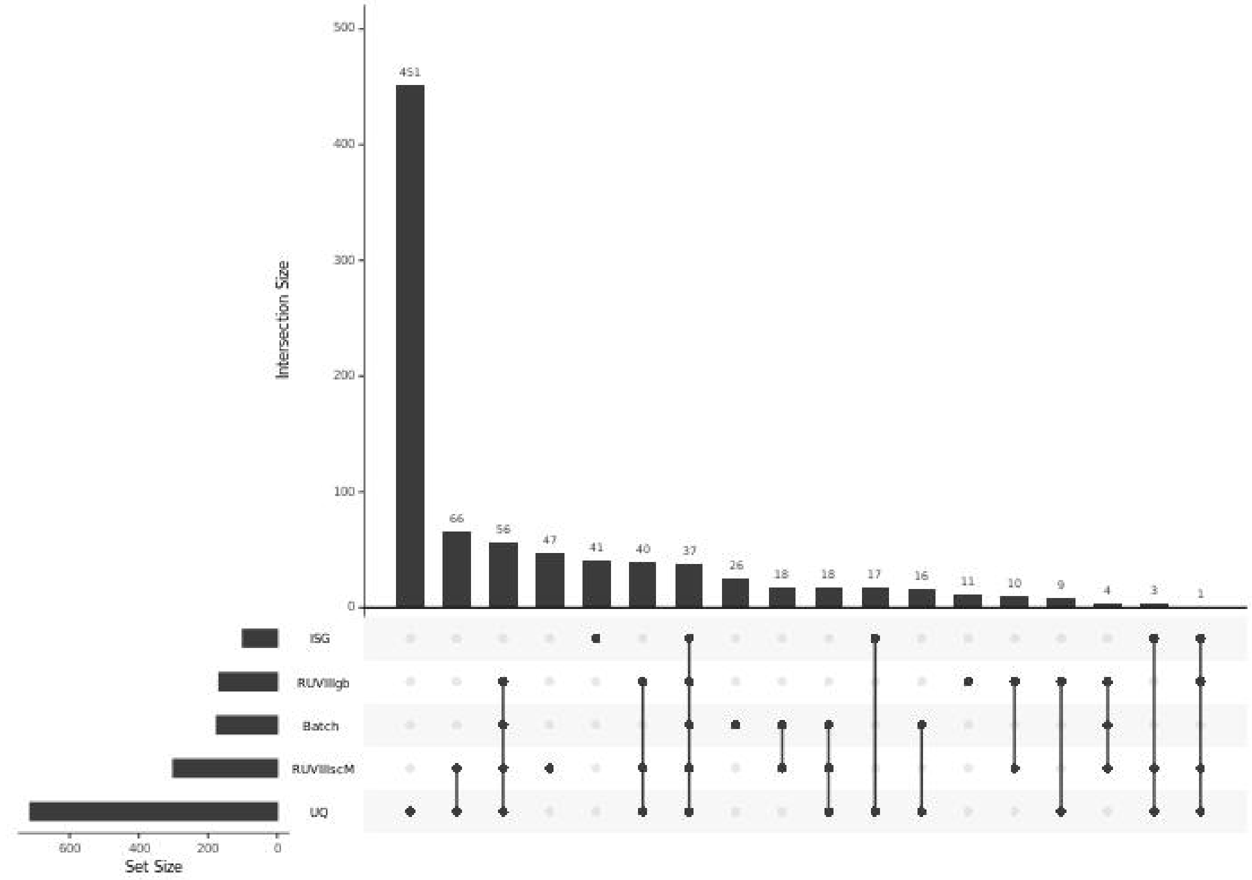

Figure S17: UpSet plot of DEGs in B cell samples from the Lupus case study, comparing the UQ, UQ Batch, and RUVIII PBPS models with the ISG signature

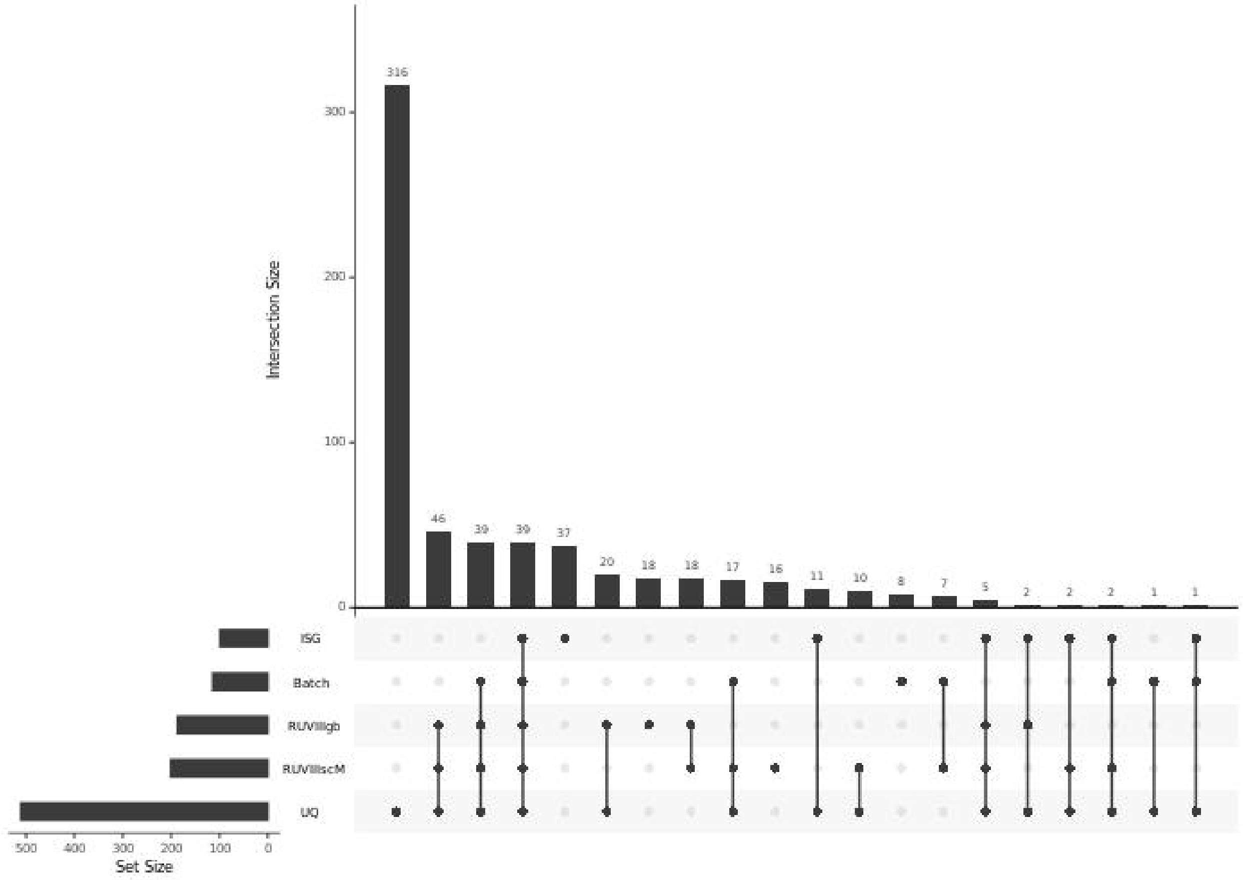

Figure S18: UpSet plot of DEGs in natural killer cell samples from the Lupus case study, comparing the UQ, UQ Batch, and RUVIII PBPS models with the ISG signature

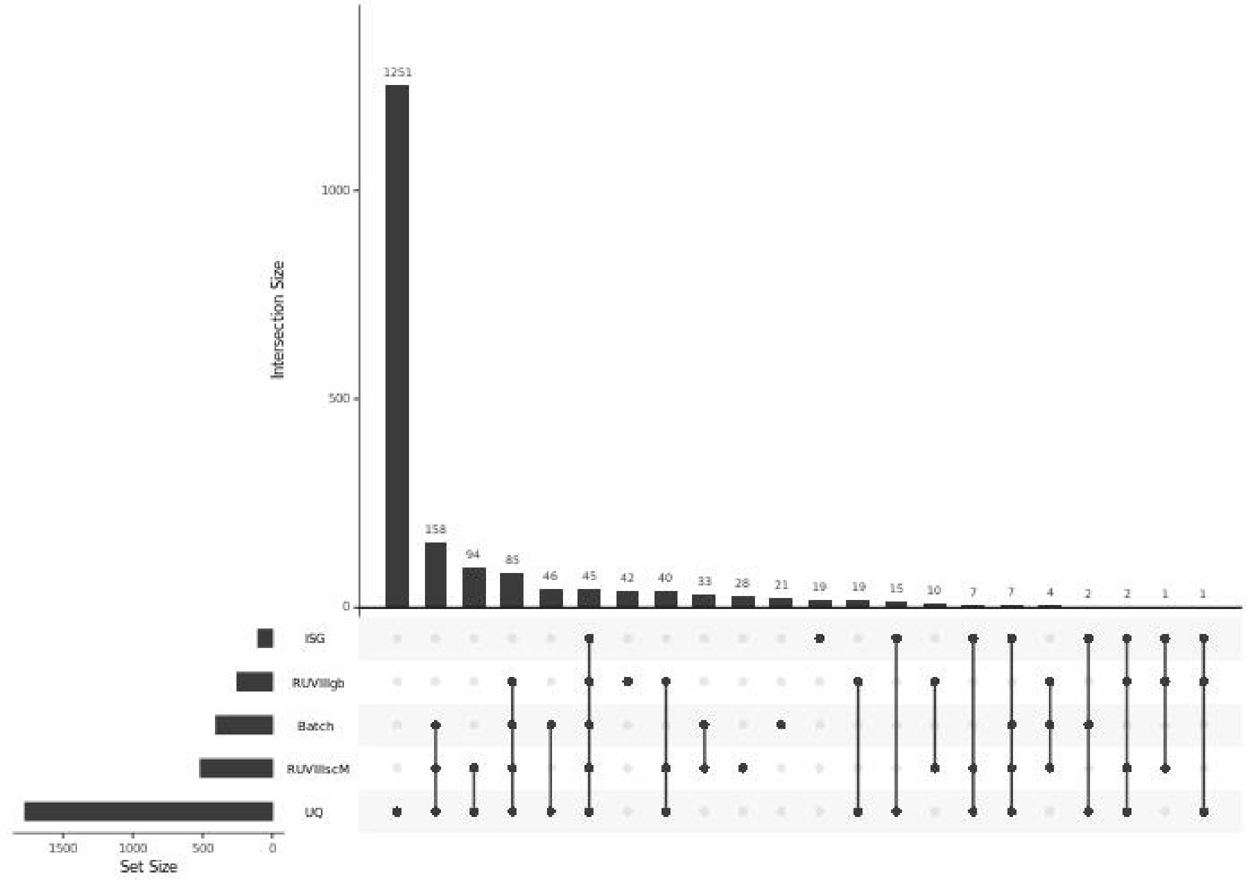

Figure S19: UpSet plot of DEGs in CD8+ T cell samples from the Lupus case study, comparing the UQ, UQ Batch, and RUVIII PBPS models with the ISG signature

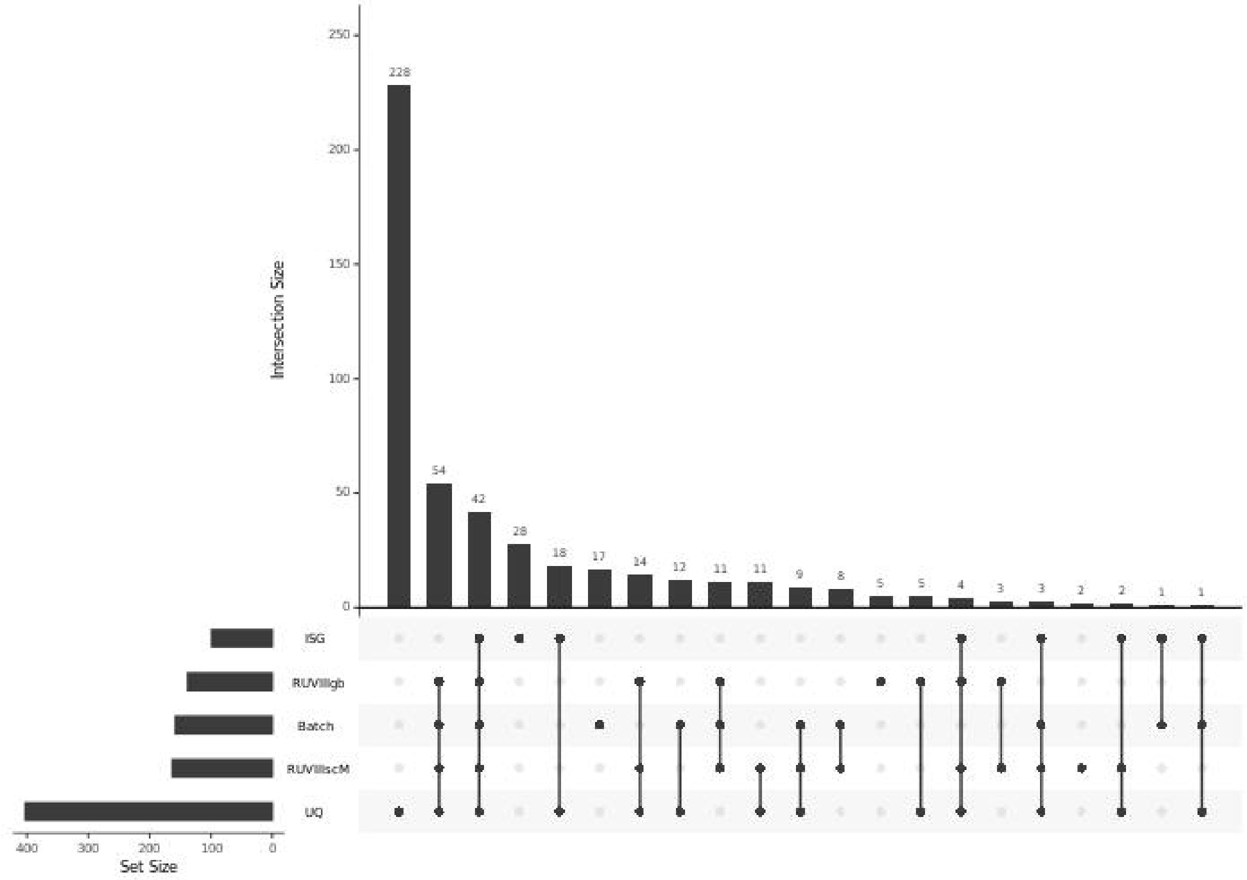

Figure S20: UpSet plot of DEGs in conventional dendritic cell samples from the Lupus case study, comparing the UQ, UQ Batch, and RUVIII PBPS models with the ISG signature

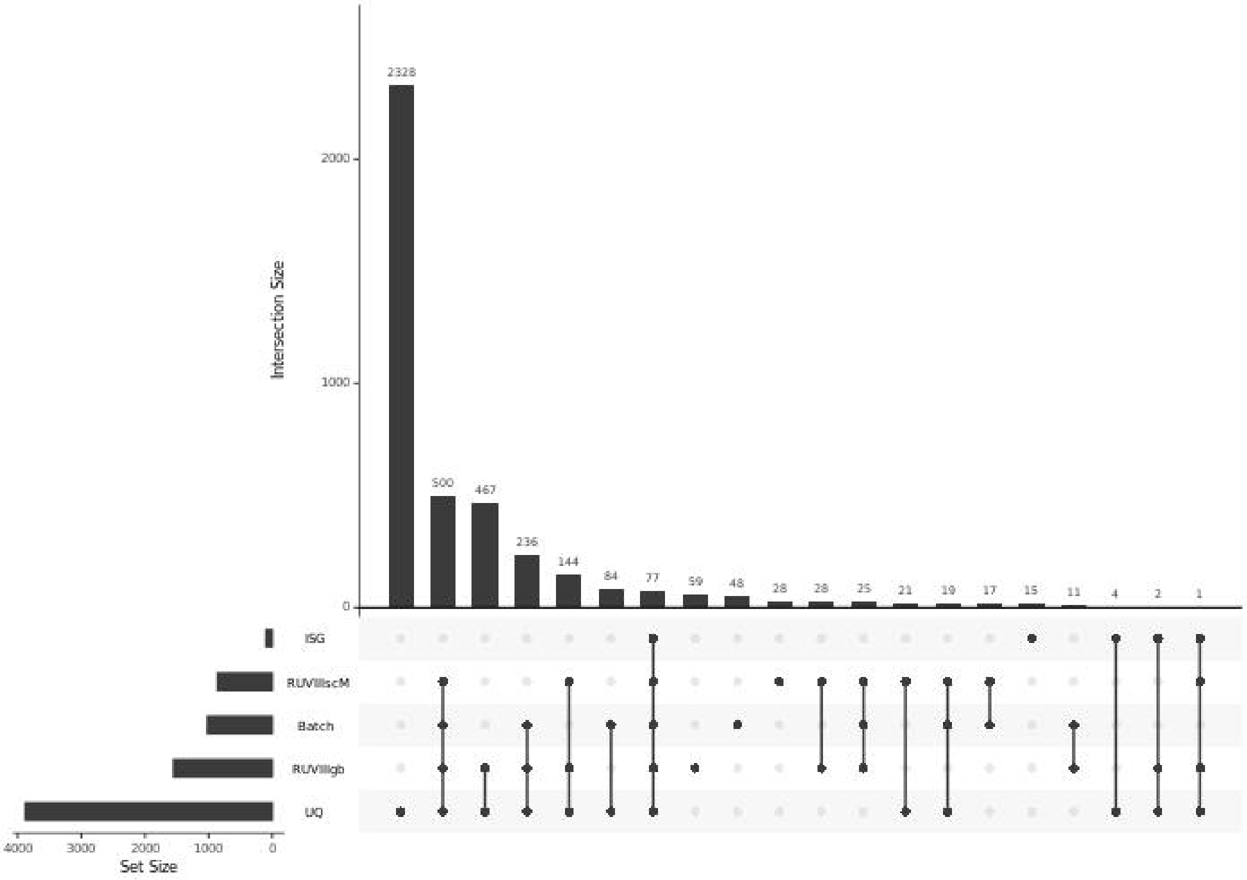

Figure S21: UpSet plot of DEGs in classical monocyte cell samples from the Lupus case study, comparing the UQ, UQ Batch, and RUVIII PBPS models with the ISG signature

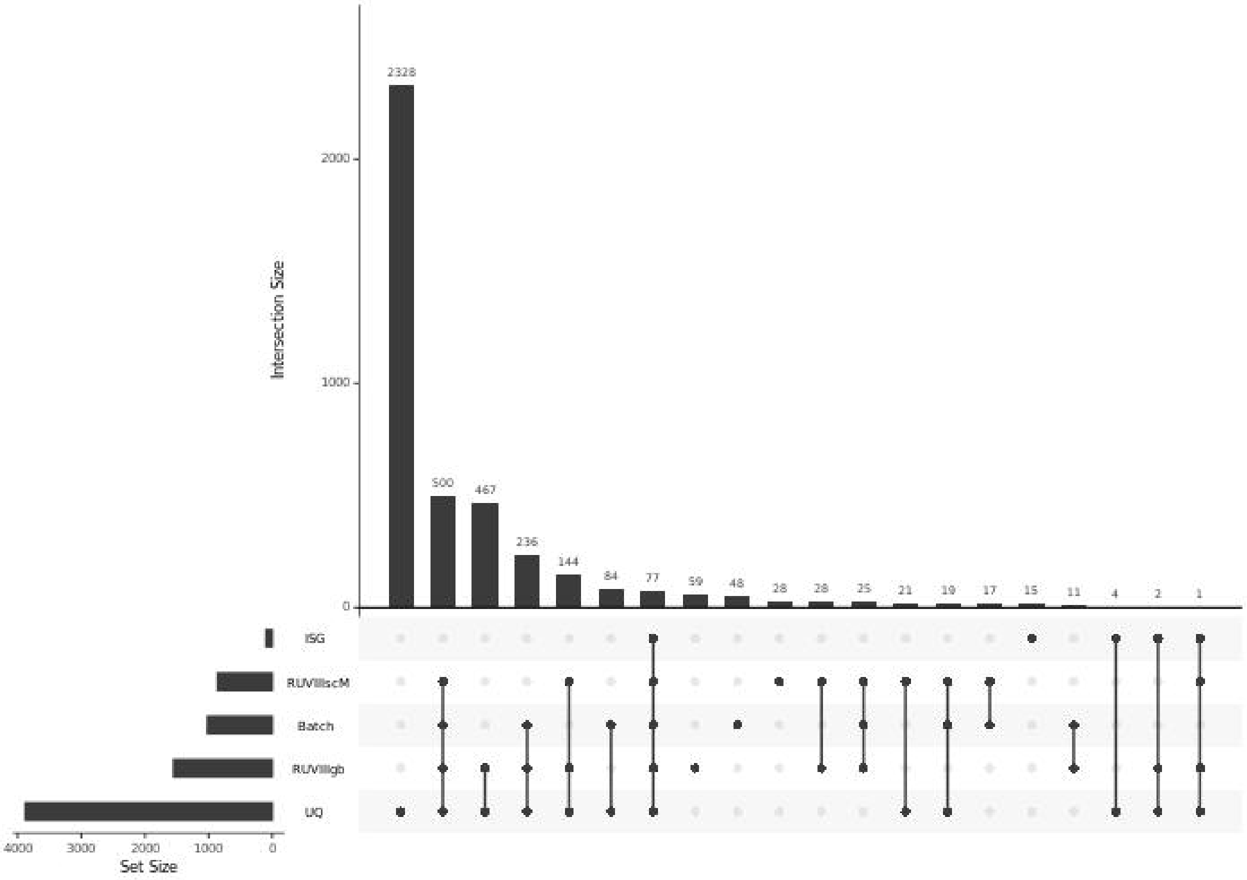

Figure S22: UpSet plot of DEGs in non-classical monocyte cell samples from the Lupus case study, comparing the UQ, UQ Batch, and RUVIII PBPS models with the ISG signature

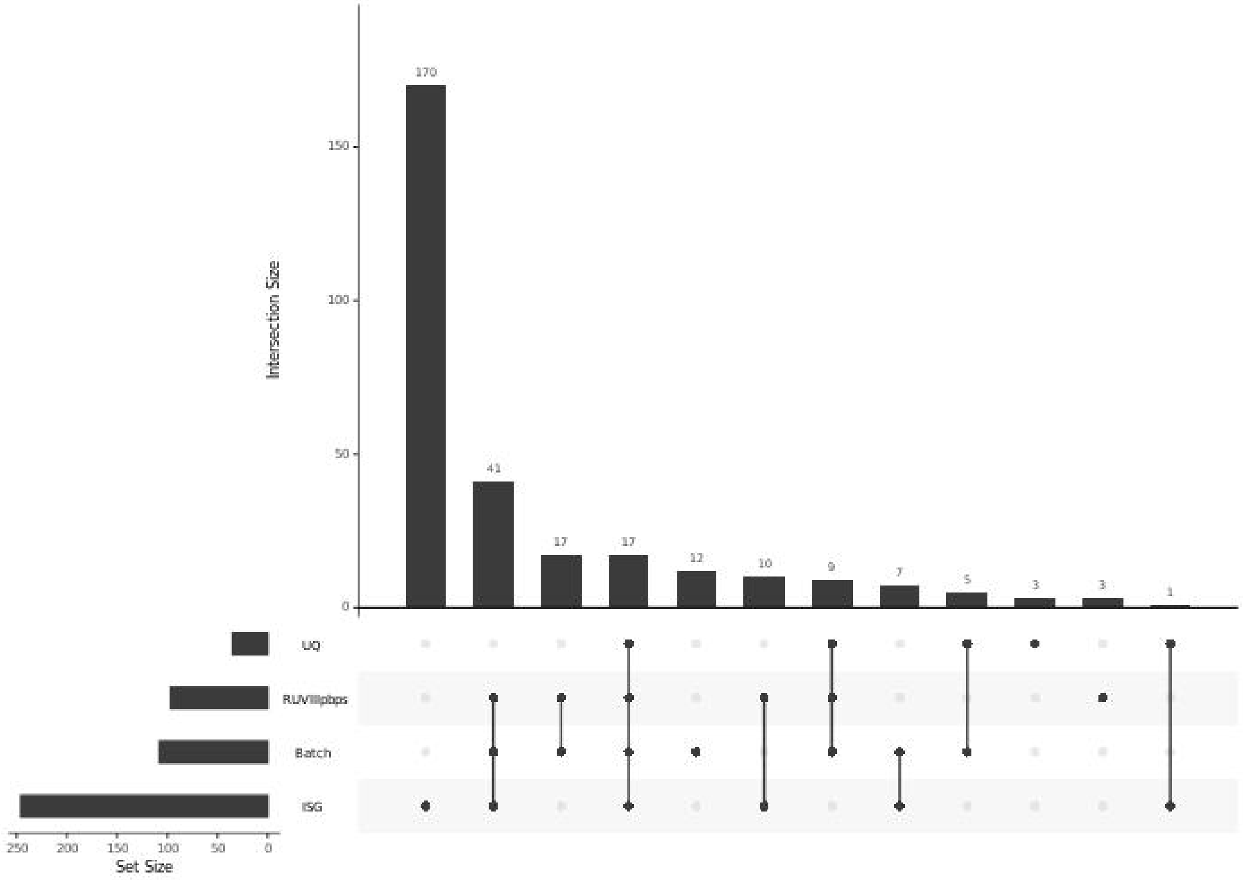

Figure S23: UpSet plot of enriched gene sets in CD4+ T samples from the Lupus case study using the UQ, UQ Batch, RUVIIIgb, RUVIIIscM models and the ISG signature

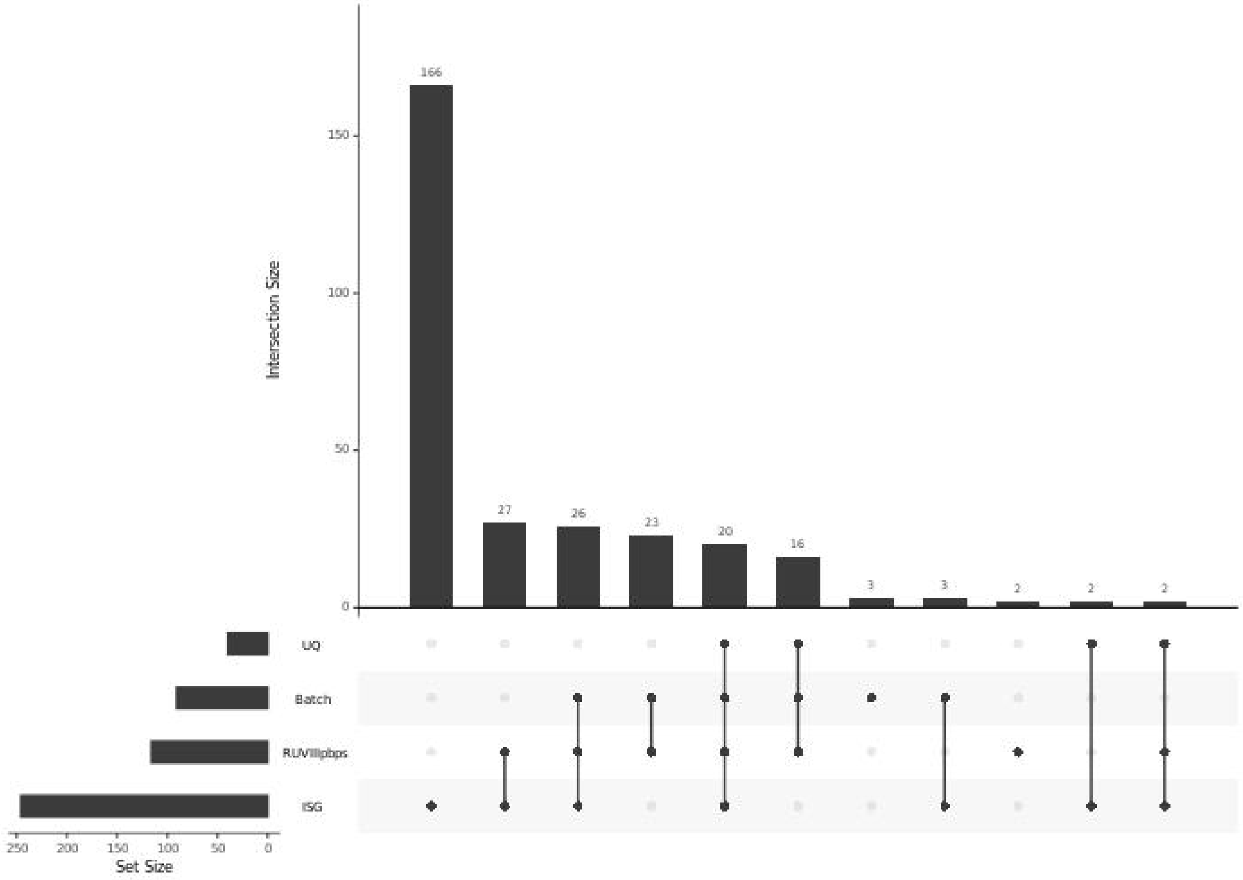

Figure S24: UpSet plot of enriched gene sets in CD8+ T samples from the Lupus case study using the UQ, UQ Batch, RUVIIIgb, RUVIIIscM models and the ISG signature

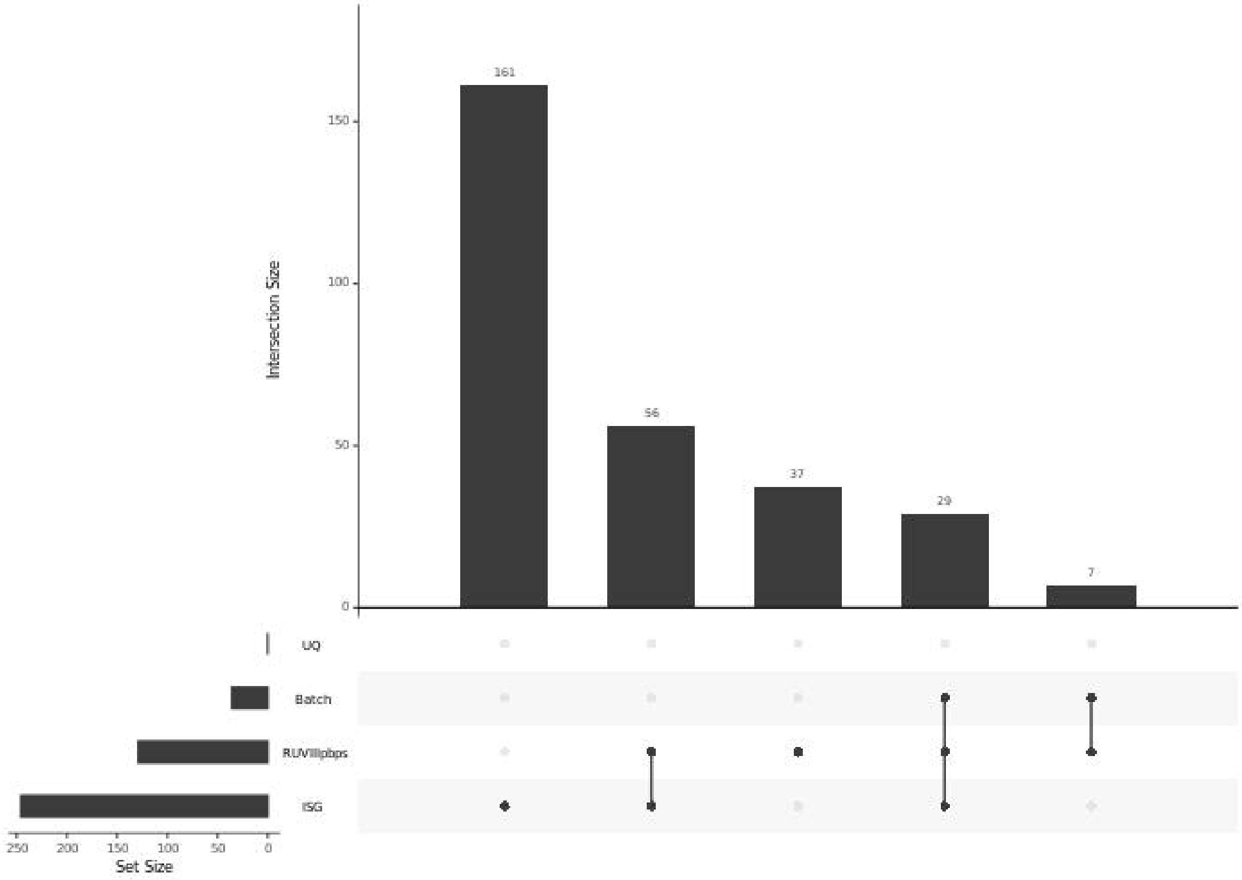

Figure S25: UpSet plot of enriched gene sets in classical monocyte samples from the Lupus case study using the UQ, UQ Batch, RUVIIIgb, RUVIIIscM models and the ISG signature

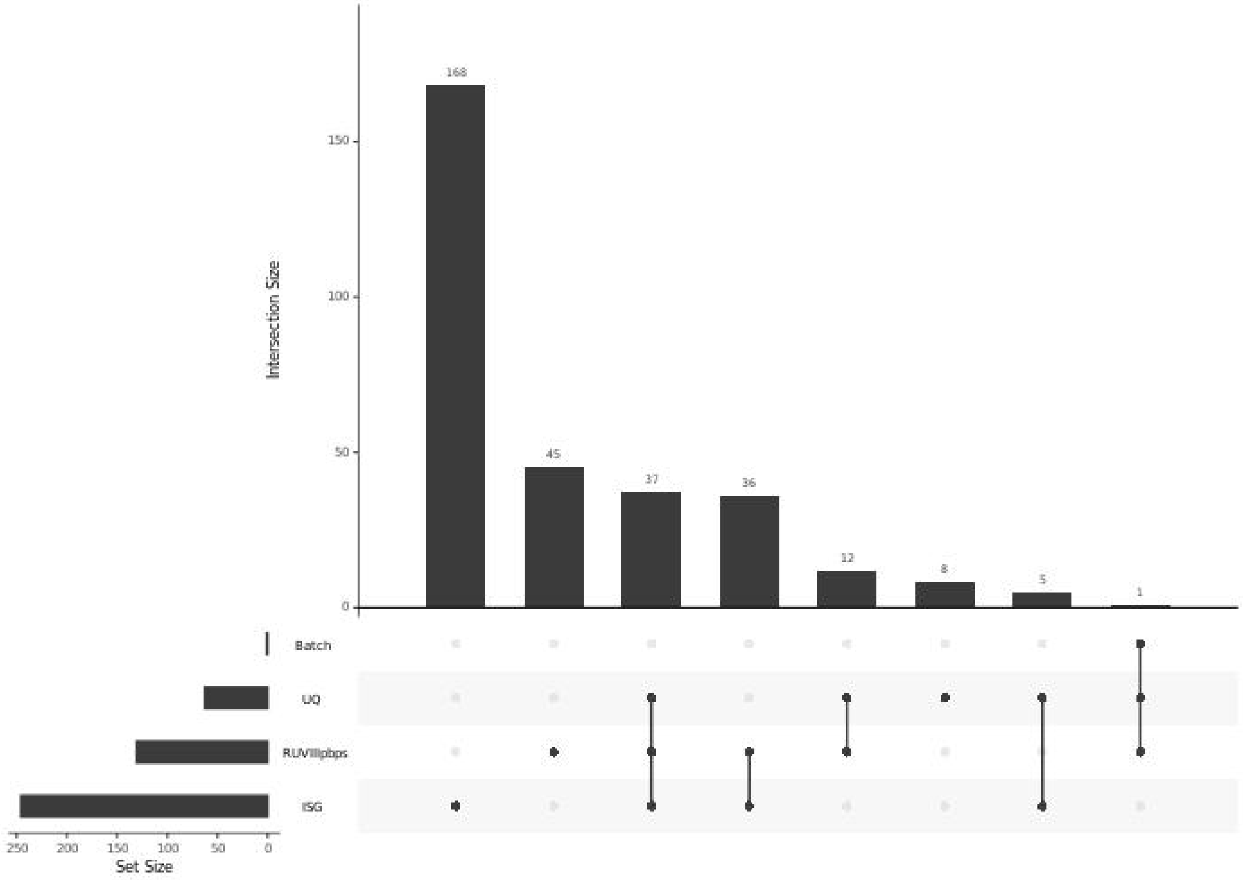

Figure S26: UpSet plot of enriched gene sets in non-classical monocyte samples from the Lupus case study using the UQ, UQ Batch, RUVIIIgb, RUVIIIscM models and the ISG signature

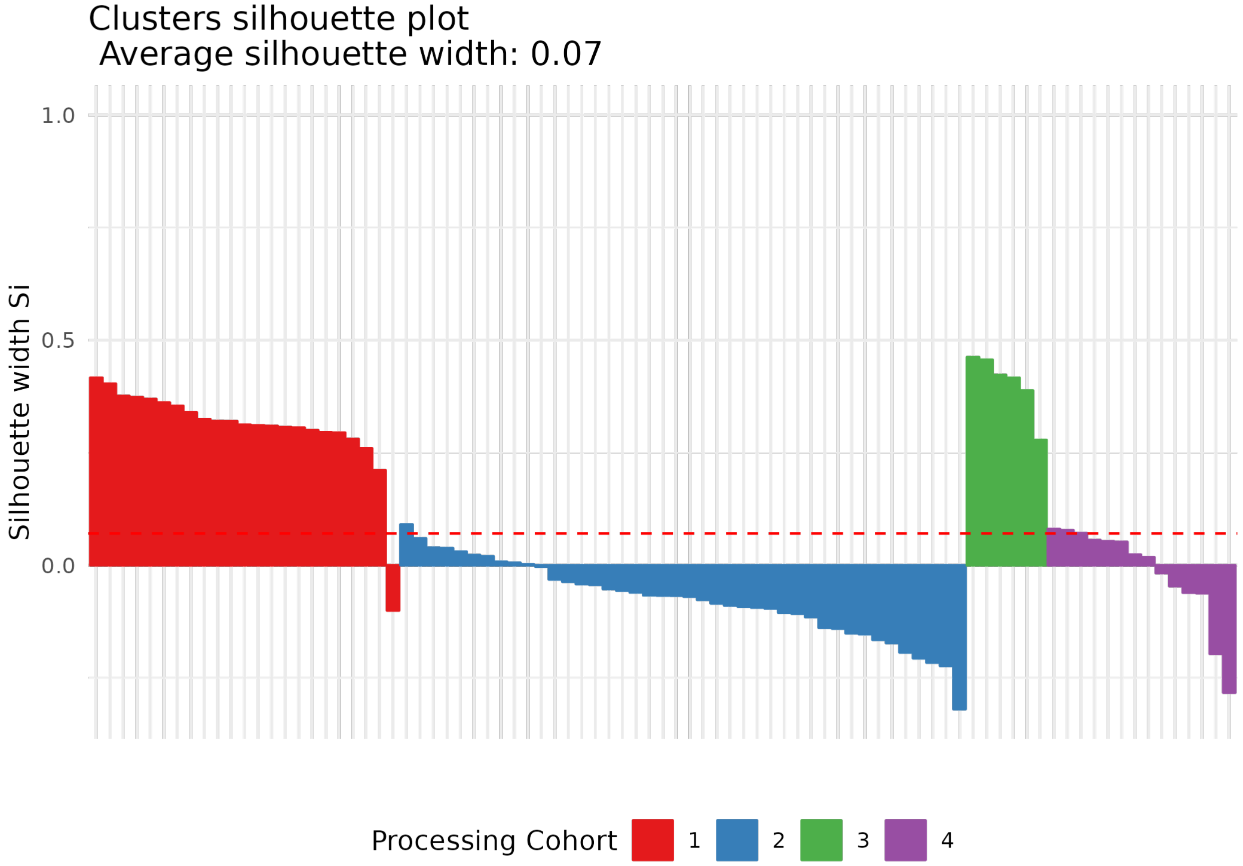

Figure S27: Processing cohort silhouette width scores of the RUVIII PBPS normalised B cell samples in the Lupus case study.

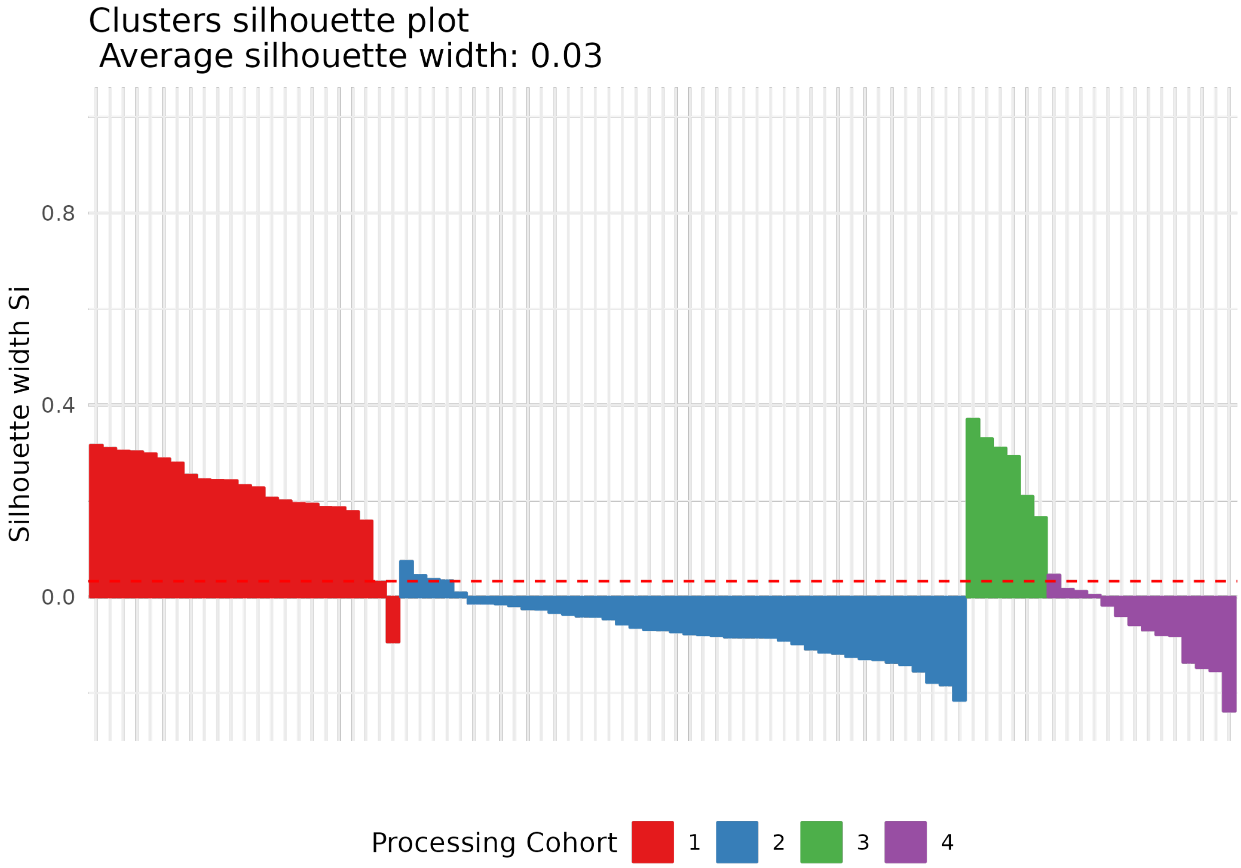

Figure S28: Processing cohort silhouette width scores of the RUVIII PBPS normalised natural killer cell samples in the Lupus case study.

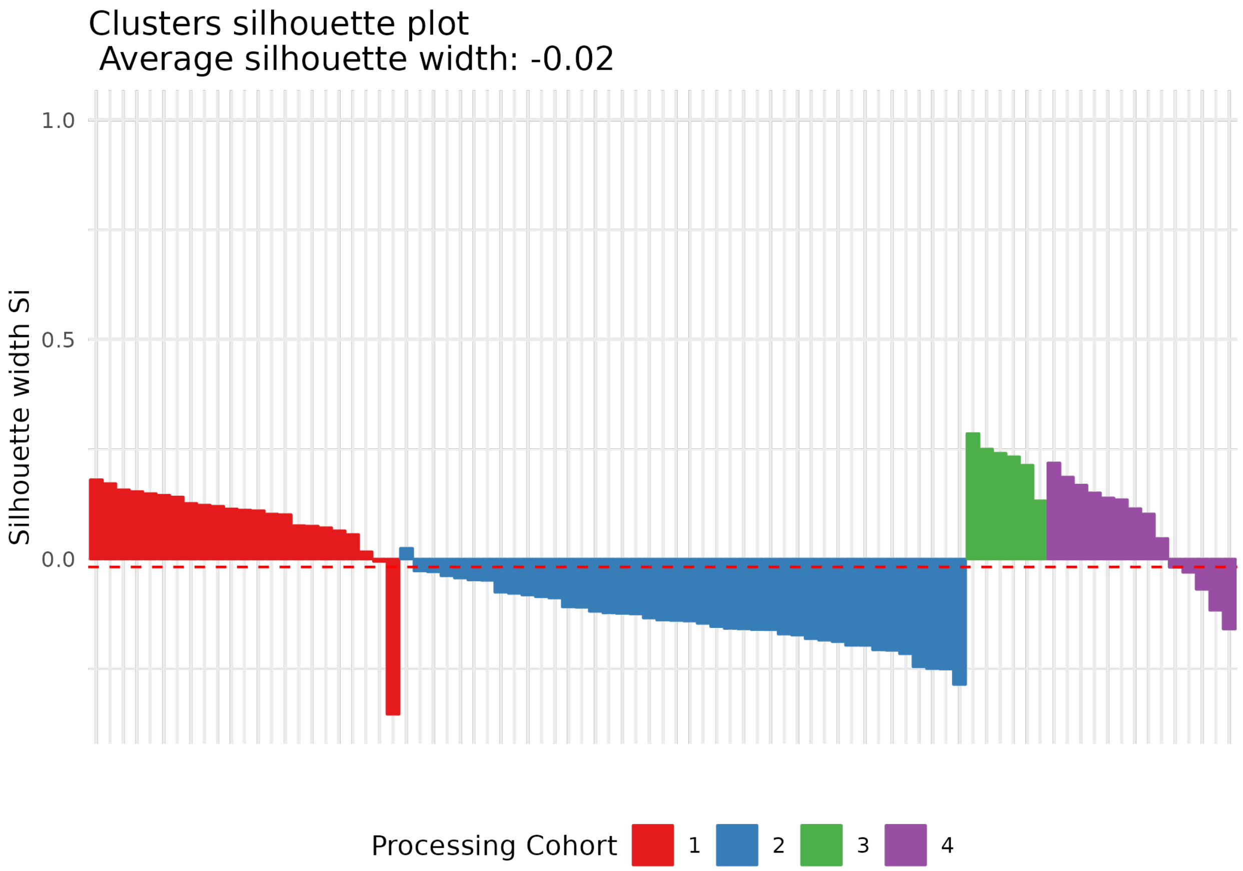

Figure S29: Processing cohort silhouette width scores of the RUVIII PBPS normalised CD8+ T cell samples in the Lupus case study.

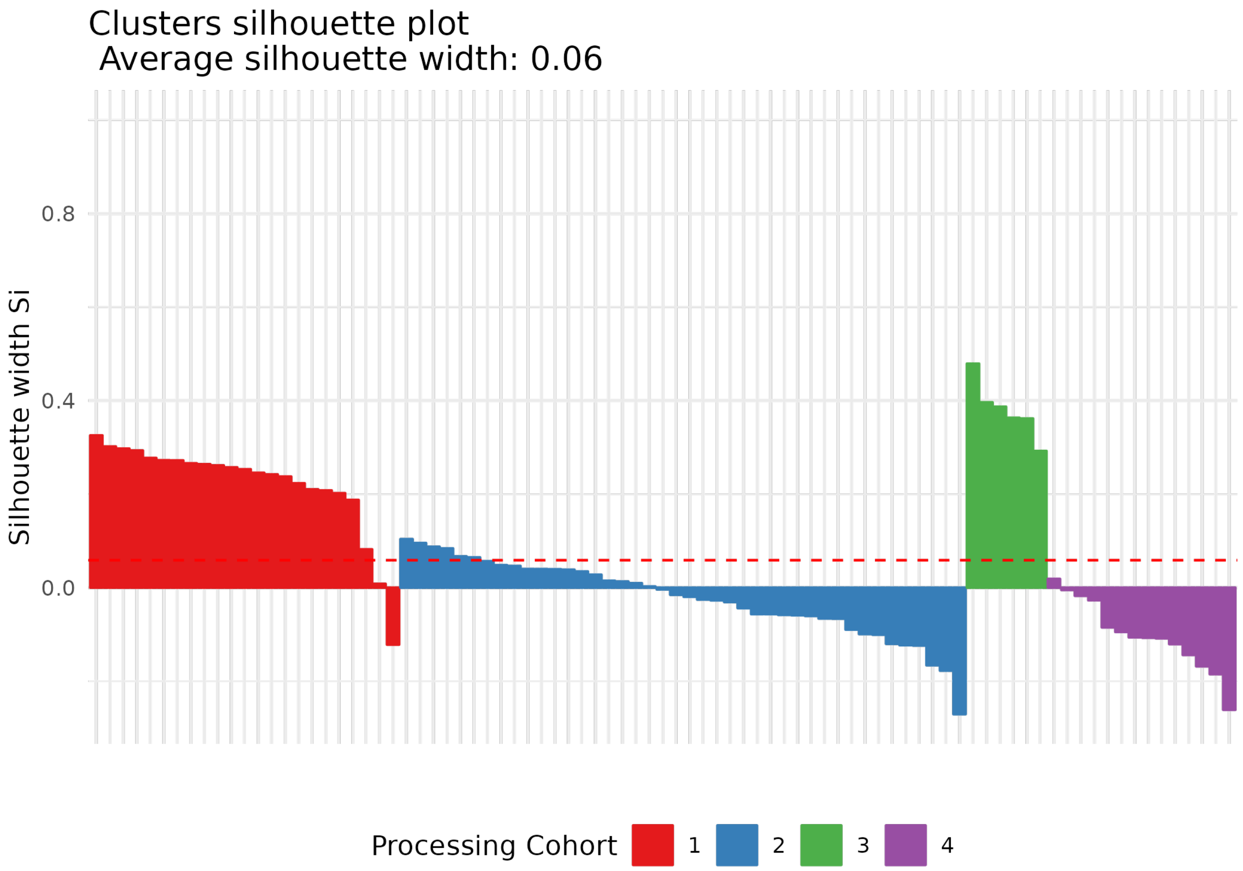

Figure S30: Processing cohort silhouette width scores of the RUVIII PBPS normalised conventional dendritic cell samples in the Lupus case study.

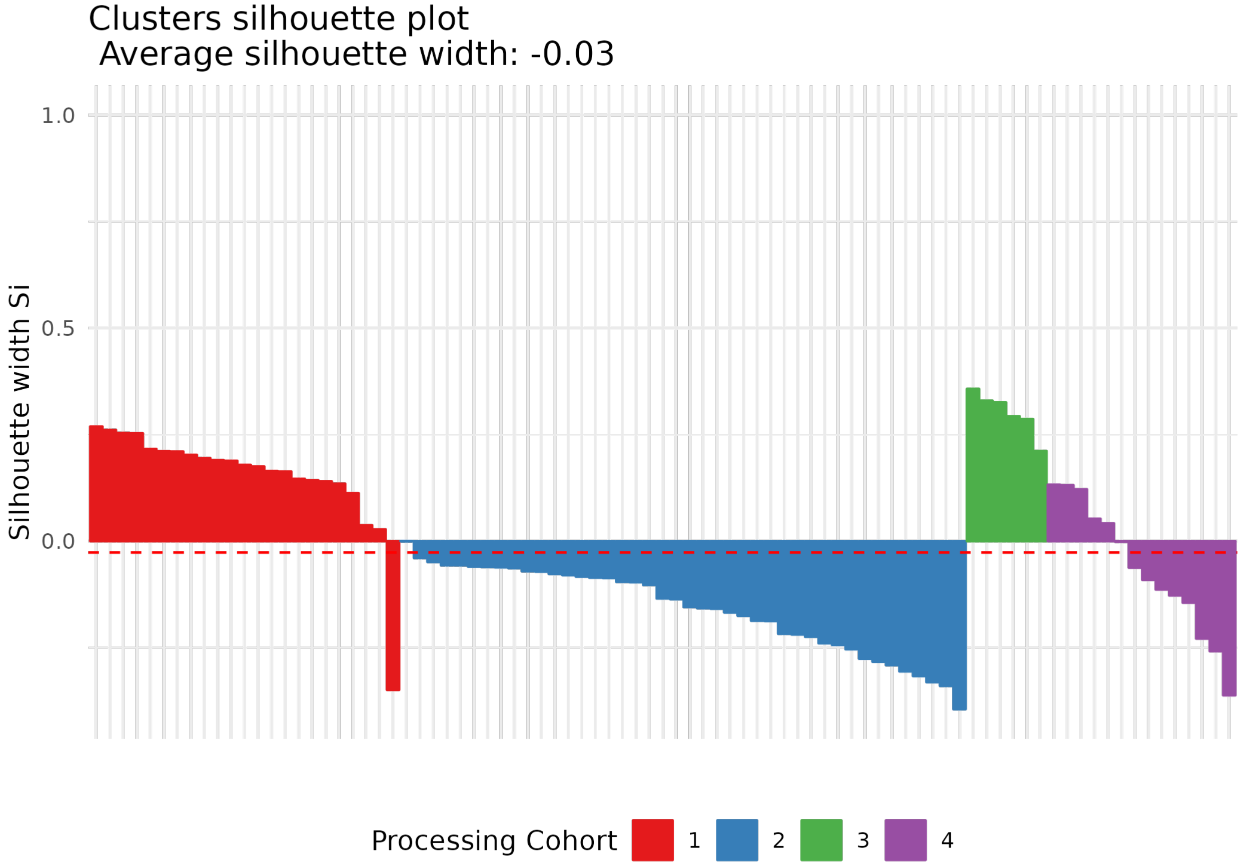

Figure S31: Processing cohort silhouette width scores of the RUVIII PBPS normalised classical monocyte samples in the Lupus case study.

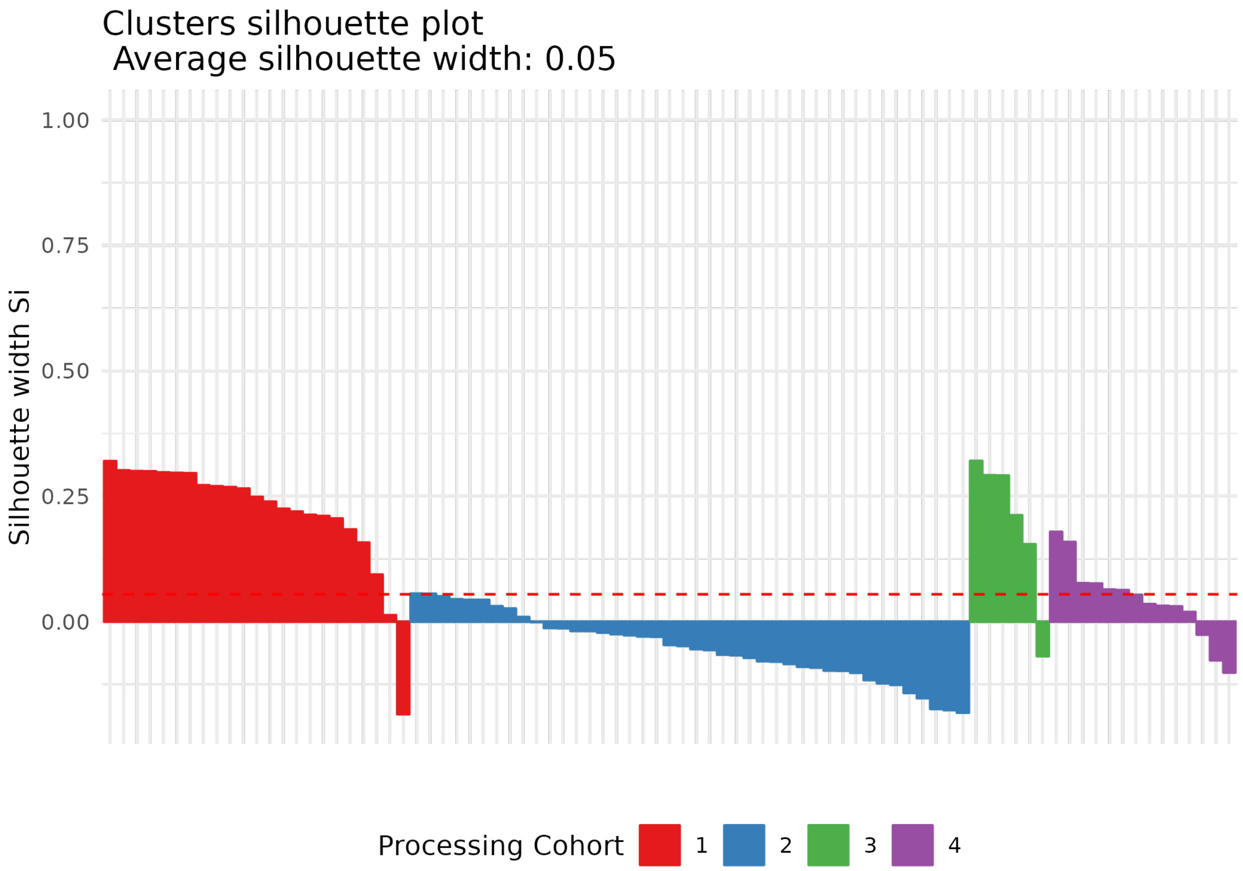

Figure S32: Processing cohort silhouette width scores of the RUVIII PBPS normalised non-classical monocyte cell samples in the Lupus case study.

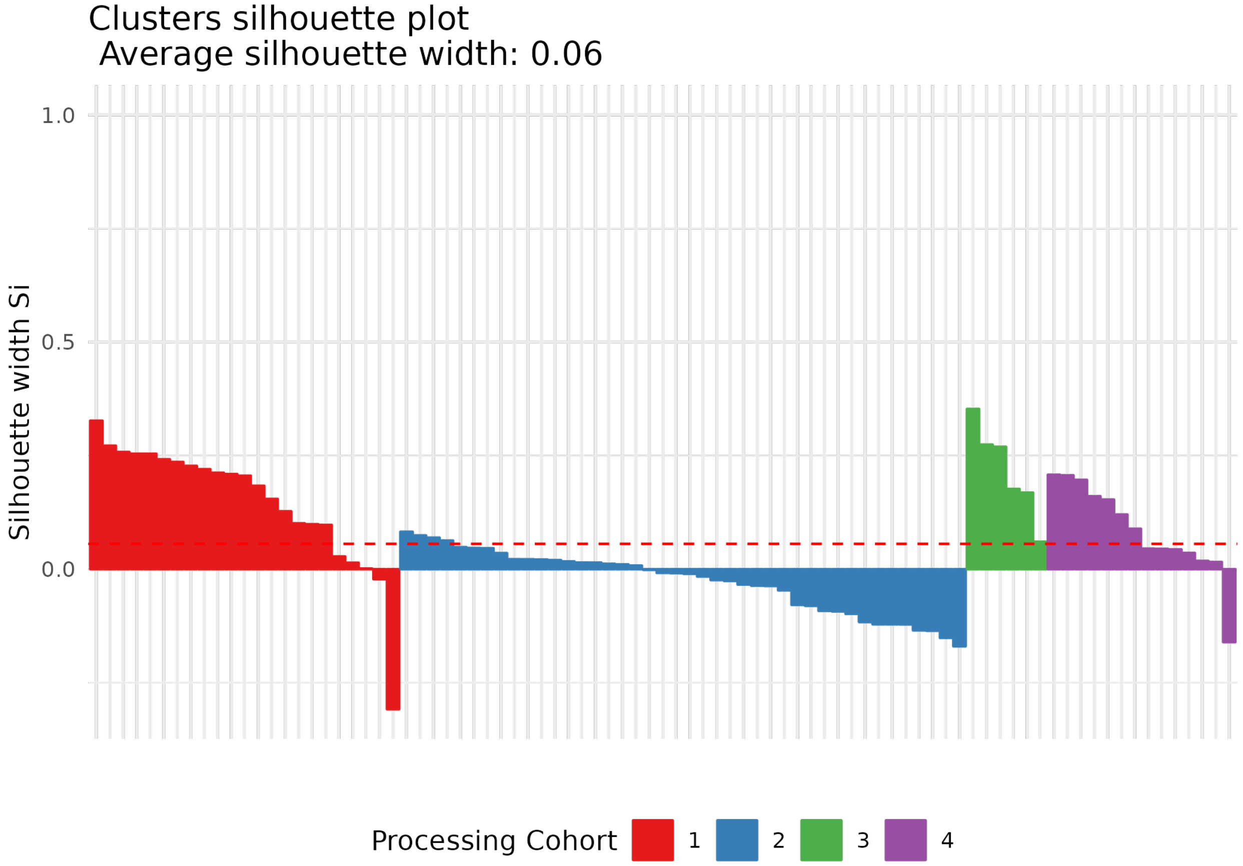

Figure S33: Processing cohort silhouette width scores of the RUVIII PBPS normalised plasmacytoid dendritic cell samples in the Lupus case study.

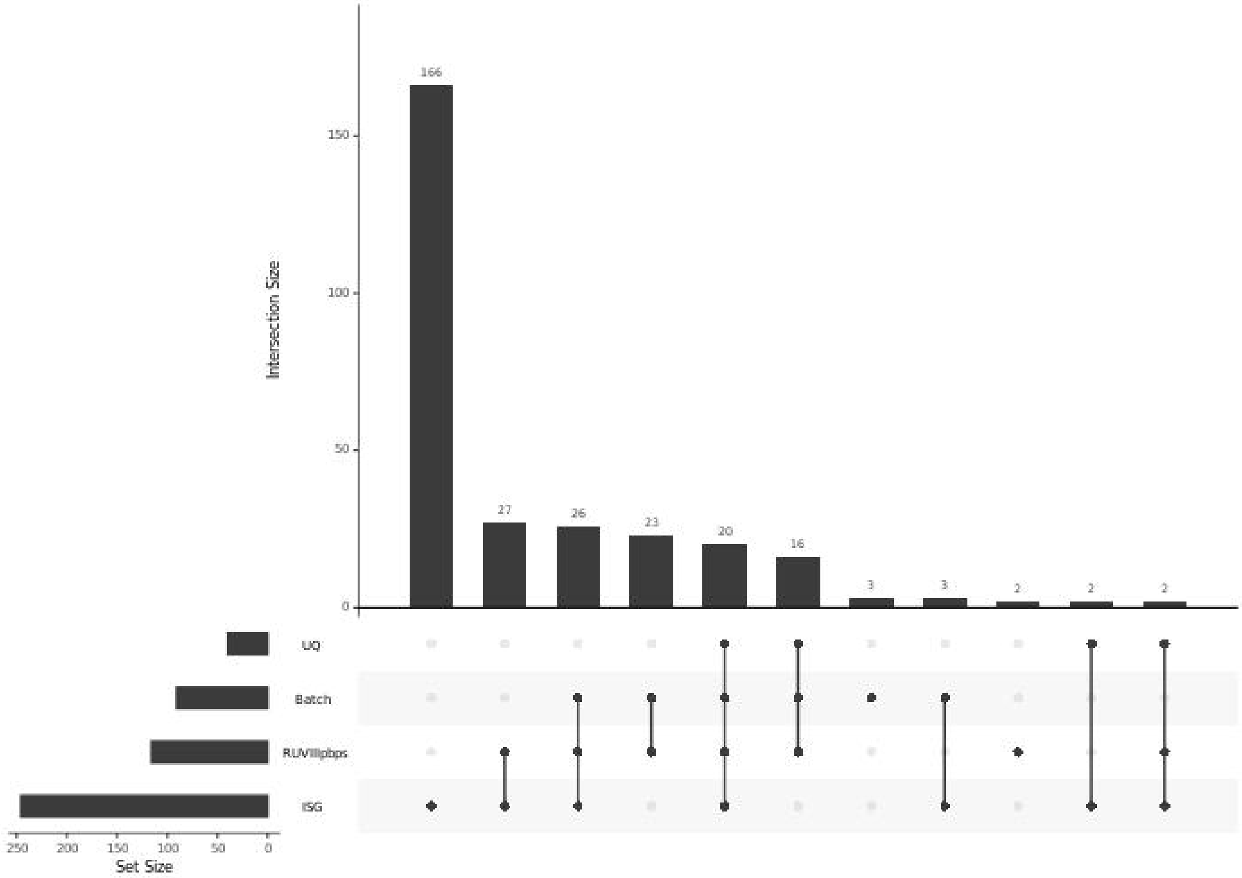

Figure S34: UpSet plot of enriched gene sets in CD8+ T samples from the Lupus case study using the UQ, UQ Batch, RUVIII PBPS models and the ISG signature.

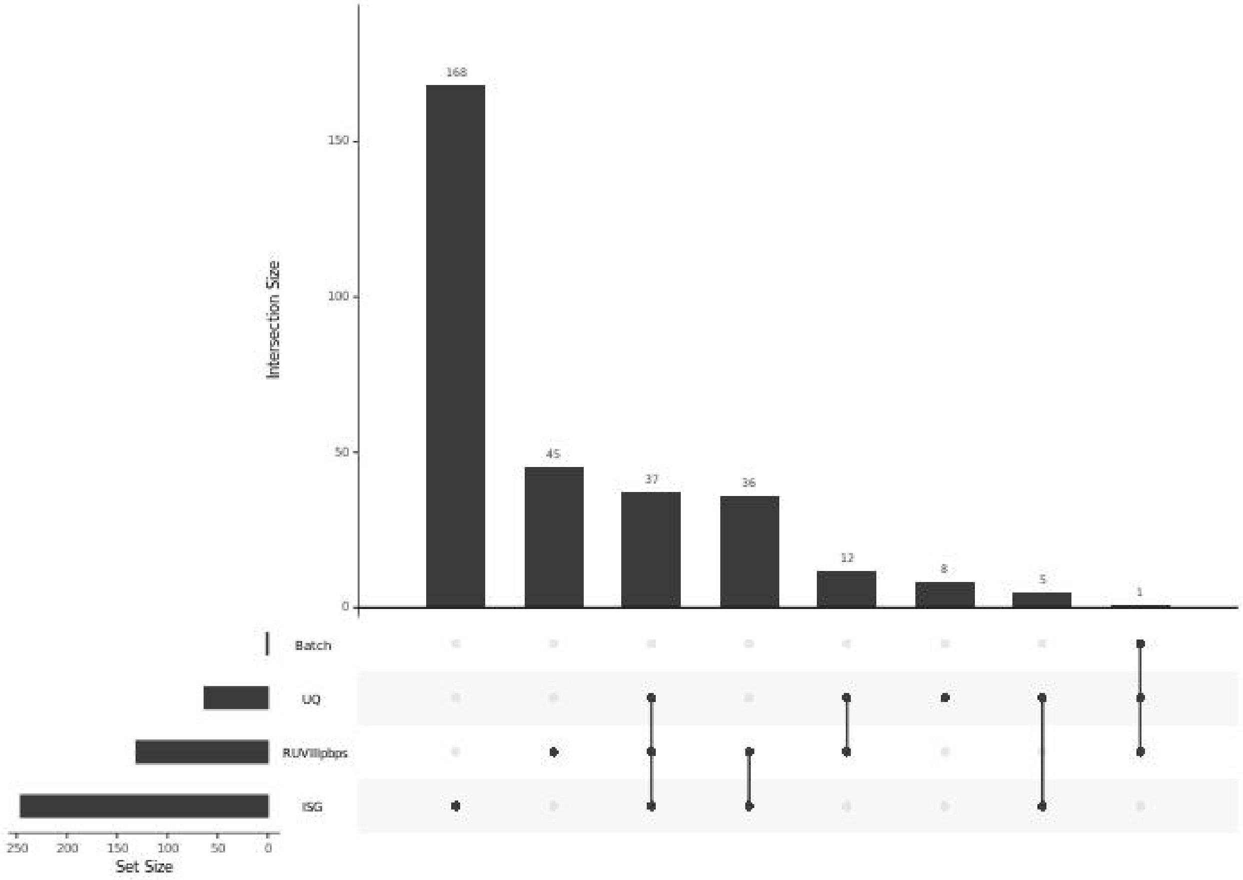

Figure S35: UpSet plot of enriched gene sets in non-classical monocyte samples from the Lupus case study using the UQ, UQ Batch, RUVIII PBPS models and the ISG signature.
